# Community analysis of pigment patterns from 37 microalgae strains reveals new carotenoids and porphyrins characteristic of distinct strains and taxonomic groups

**DOI:** 10.1371/journal.pone.0171872

**Published:** 2017-02-23

**Authors:** Benoît Serive, Elodie Nicolau, Jean-Baptiste Bérard, Raymond Kaas, Virginie Pasquet, Laurent Picot, Jean-Paul Cadoret

**Affiliations:** 1 Laboratoire de Physiologie et Biotechnologie des Algues, IFREMER, BP, Nantes, France; 2 UMRi CNRS 7266 LIENSs, Université de la Rochelle, La Rochelle, France; Stazione Zoologica Anton Dohrn, ITALY

## Abstract

Phytoplankton, with an estimated 30 000 to 1 000 000 species clustered in 12 phyla, presents a high taxonomic and ecophysiological diversity, reflected by the complex distribution of pigments among the different algal classes. High performance liquid chromatography is the gold standard method for qualitative and quantitative analysis of phytoplankton pigments in seawater and culture samples, but only a few pigments can be used as robust chemotaxonomic markers. A major challenge is thus to identify new ones, characteristic of a strain, species, class or taxon that cannot be currently identified on the basis of its pigment signature. Using an optimized extraction process coupled to a HPLC de-replication strategy, we examined the pigment composition of 37 microalgae strains, representative of the broad taxonomic diversity of marine and freshwater species (excluding cyanobacteria). For each species, the major pigments already described were unambiguously identified. We also observed the presence of several minor unidentified pigments in each chromatogram. The global analysis of pigment compositions revealed a total of 124 pigments, including 98 pigments or derivatives unidentified using the standards. Absorption spectra indicated that 35 corresponded to chlorophyll/porphyrin derivatives, 57 to carotenoids and six to derivatives having both spectral signatures. Sixty-one of these unidentified or new carotenoids and porphyrin derivatives were characteristic of particular strains or species, indicating their possible use as highly specific chemotaxonomic markers capable of identifying one strain out of the 37 selected. We developed a graphical analysis using Gephi software to give a clear representation of pigment communities among the various phytoplankton strains, and to reveal strain-characteristic and shared pigments. This made it possible to reconstruct the taxonomic evolution of microalgae classes, on the basis of the conservation, loss, and/or appearance of pigments.

## Introduction

Photosynthetic microorganisms have evolved a wide range of photoprotective and photosynthetic pigments capable of collectively harvesting most of the wavelengths of visible light available in underwater marine habitats [1]. This chemodiversity reflects the diverse molecular adaptations to the multiple photic conditions met over the evolution of microalgae taxa in marine ecosystems. In spite of their lability, complex distribution among phytoplankton classes, and variable expression, pigments are of great interest as chemotaxonomic markers to identify species or taxa, and assess their abundance, productivity and biodiversity in seawater samples containing different phytoplankton communities [2–4] The identification and dosage of pigments and derivatives in sediments also provides a useful way to assess the ocean productivity, model the spatial and seasonal sedimentation and hydrodynamic processes, and demonstrate local or global marine ecosystem changes [5–7]. In recent decades, HPLC has emerged as the gold standard analytical tool for qualitative and quantitative analysis of phytoplankton pigments in seawater and culture samples because of its ease of use, rapidity, sensitivity, resolution, and potential for development on research vessels [2,8–12]. HPLC is the technique of choice for the standardized quantification of chlorophyll *a* and identification and quantification of minor pigments. It is also used as the reference method for the validation of other chlorophyll *a* measurement techniques, including remote sensing dosage. Since 1980, methodological recommendations and optimized protocols have been proposed by the SCOR/UNESCO research group and NASA for reliable phytoplankton pigment analysis and inter-calibration studies. Among others, the Van Heukelem & Thomas (2001) [13] method is currently one of the most efficient and recommended to analyse microalgal organic pigments. Methodological optimization of HPLC performance demonstrated that, in addition to the major pigments easily identified by their absorbance spectrum, band ratio and polarity, several minor unidentified chlorophyll and carotenoids derivatives are usually present in extracts from environmental samples or cultivated species. Most of the time, because of their very low abundance and fastidious purification, these minor pigments are not identified, despite their possible interest as chemotaxonomic markers or for biotechnological or biomedical applications. They can correspond to molecules effectively present in living algal cells, to biosynthetic precursors and intermediates, or to artefacts or natural derivatives/molecules produced by the alteration of chlorophylls or carotenoids in environmental conditions or during extraction and/or purification. One of the major challenges faced by scientists involved in phytoplankton research is to identify among these molecules those that unambiguously signal the presence of species, genera or classes, and can be used as robust chemotaxonomic markers for the molecular fingerprinting of phytoplankton. Another challenge is to develop standardized protocols allowing the rapid identification and dosage of these biomarkers for their convenient use in routine analysis of phytoplankton samples.

We recently developed and optimized an efficient phytoplankton pigment extraction method that limits pigment degradation and enhances extraction yields and access to pigments strongly linked to the thylakoid membranes [14]. By coupling this optimized extraction protocol to standardized Van Heukelem & Thomas HPLC analysis [13], we examined the pigment composition of 37 microalgae strains (excluding prokaryotes), representative of the broadest possible taxonomic diversity of marine and freshwater species. The objectives were (i) to provide a comprehensive analysis of the pigment chemodiversity of eukaryote photosynthetic microorganisms; (ii) to propose new potential chemotaxonomic markers to improve algal assemblage determination in open waters; (iii) to pinpoint ancillary carotenoid or porphyrin derivatives with original spectral properties for chemotaxonomy.

## Methods

### Phytoplankton strains

Thirty-seven phytoplankton strains belonging to 34 different species and 26 different genera were selected to provide as representative selection as possible of the taxonomic diversity of eukaryotic marine and freshwater species (Table 1). Strains were selected to include 15 major classes of microalgae, among the species the most studied in labs worldwide, and their taxonomic status was determined according to the international Algae Base taxonomic classification http://www.algaebase.org (accessed on 2016-03-27). All strains used in this study are referenced in international algae culture collections, including SAG, CCAP, CCMP, UTEX and IFREMER collections.

**Table 1:** Microalgae strain origin and culture condition.

Phylum	Class	Family	Strain code	Short name	Origin of the strain	Culture condition
**Eukaroyte Plantae**						
**Rhodophyta**	Porphyridiophyceae	Porphyridiaceae	*Porphyridium purpureum* **SAG 1380-1d**	Pp	SAG	SW—Walne’s medium
	Rhodellophyceae	Rhodellaceae	*Rhodella violacea* **SAG 115.79**	Rv	SAG	SW—Walne’s medium
	Cyanidiophyceae	Galdieriaceae	*Galdieria sulphuraria*	Gs	Ifremer	FW—Walne’s medium—SA
**Glaucophyta**	Glaucophyceae	Glaucocystaceae	*Cyanophora paradoxa* **SAG 29.80**	Cp	SAG	FW—Walne’s medium
**Charophyta**	Conjugatophyceae	Closteriaceae	*Closterium baillyanum* **SAG 50.89**	Cb	SAG	FW—Walne’s medium—CaCO_3_
**Chlorophyta**	Chlorophyceae	Scenedesmaceae	*Scenedesmus acutus f*. *alternans* **UTEX 72**	Saa	UTEX	FW—Walne’s medium
			*Scenedesmus obliquus* **UTEX 1450**	So	UTEX	FW—Walne’s medium
		Haematococcaceae	*Haematococcus pluvialis* **CCAP 34/7**	Hp	CCAP	FW—Walne’s medium
		Dunaliellaceae	*Dunaliella* sp. **CCAP 19/19**	Dsp	CCAP	SW—Walne’s medium
			*Dunaliella salina* **SAG 19–3**	Ds	SAG	SW—Walne’s medium
	Trebouxiophyceae	Chlorellaceae	*Chlorella autotrophica* **CCMP 243**	Ca	CCMP	SW—Walne’s medium
			*Chorella vulgaris* **SAG 2.80**	Cv	SAG	FW—Walne’s medium
	Mamiellophyceae	Bathycoccaceae	*Ostreococcus tauri* **H95**	Ot	Ifremer	SW—Walne’s medium
	Chlorodendrophyceae	Chlorodendraceae	*Tetraselmis suecica* **CCMP 904**	Ts	CCMP	SW—Walne’s medium
**Eukaryote Chromista**						
**Cercozoa**	Chlorarachniophyceae	Chlorarachniaceae	*Chlorarachnion reptans* **SAG 26.97**	Cr	SAG	SW—Walne’s medium—Si—SE
			*Bigelowiella natans* **CCMP 621**	Bn	CCMP	SW—F/2—Si
**Haptophyta**	Coccolithophyceae (Prymnesiophyceae)	Isochrysidaceae	*Isochrysis galbana* **SAG 13.92**	Ig	CCAP	SW—Walne’s medium
			*Tisochrysis lutea* **CCAP 927/14**	Tl	CCAP	SW—Walne’s medium
		Noelaerhabdaceae	*Emiliania huxleyi* **SAG 33.90**	Eh	SAG	SW—Walne’s medium—CaCO_3_
**Bacillariophyta**	Mediophyceae	Thalassiosiraceae	*Thalassiosira pseudonana* **CCMP 1335**	Tp	CCMP	SW—Walne’s medium- Si
		Skeletonemataceae	*Skeletonema grethae* **CCAP 1077/4**	Sg	CCAP	SW—Walne’s medium- Si
		Eupodiscacea	*Odontella aurita* **123**	Oa1	Ifremer	SW—Walne’s medium- Si
			*Odontella aurita* **122**	Oa2	Ifremer	SW—Walne’s medium- Si
		Chaetocerotaceae	*Chaetoceros mulleri*	Cmu	Ifremer	SW—Walne’s medium- Si
			*Chaetoceros minus*	Cmi	Ifremer	SW—Walne’s medium- Si
			*Chaetoceros* sp. *Tenuissimus like*	Ctl	Ifremer	SW—Walne’s medium- Si
			*Chaetoceros calcitrans f*. *pumillum* **CCAP 1010/11**	Ccp	CCAP	SW—Walne’s medium- Si
			*Chaetoceros calcitrans* **CCMP 1315**	Cc	CCMP	SW—Walne’s medium- Si
			*Chaetoceros gracilis* **UTEX LB 2658**	Cg	UTEX	SW—Walne’s medium- Si
	Bacillariophyceae	Bacillariaceae	*Nitzschia* sp. **CCMP 2526**	Nsp	CCMP	SW—Walne’s medium- Si
	Bacillariophyceae incertae sedis	Phaeodactylaceae	*Phaeodactylum tricornutum* **CCMP 632**	Pt	CCMP	SW—Walne’s medium—Si
**Cryptophyta**	Cryptophyceae	Pyrenomonadaceae	*Rhodomonas salina* **CCAP 978/27**	Rs	CCAP	SW—Walne’s medium
**Eukaryote protozoa**						
**Miozoa**	Dinophyceae	Goniodomataceae	*Alexandrium tamarense* **MOG 835** (Toxic)	At1	Ifremer	SW—ESP
			*Alexandrium tamarense* **PLY 497A** (non toxic)	At2	Ifremer	SW—ESP
			*Alexandrium minutum* **CM1002** (Non toxic)	Am1	Marine Institute	SW—F/2
			*Alexandrium minutum* **AM89BM** (Toxic)	Am2	Ifremer	SW—L1
**Euglenophyta**	Euglenophyceae	Euglenaceae	*Euglena proxima* **SAG 1224-11a**	Ep	SAG	FW—Walne’s medium

Classification was established according to Guiry, M.D. & Guiry, G.M. 2016. *AlgaeBase*. Worldwide electronic publication, National University of Ireland, Galway. http://www.algaebase.org; searched on 27^th^ March 2016. SW: Seawater; FW: Freshwater; Si: Silicates (0,4g.L^-1^); CaCO_3_: Calcium carbonate (0,25 mg.L^-1^); SE: Soil extract (0,5 mL.L^-1^); SA: Sulfuric acid (final medium pH = 2); ESP: Bold’s basal medium + proteose peptone + soil extract + vitamins.; F/2: Guillard medium; L1: Modified F/2 medium. Incertae sedis classification is displayed where phenotypic characteristics are not supported by genomic data. Colours distinguish the taxonomic lineages and outsider phyla in terms of pigmentation.

### Culture and collection

Microalgae were grown in sterile 100 mL flasks under continuous light (120 μmol.m^-2^.s^-1^) provided by fluorescent lamps (Philips TLD 58W 865), at 20°C in normalized growth medium for each strain (Table 1). Standardization of the culture parameters allowed the quantitative interspecific comparison of pigment production yields (Chromatograms in Supporting Information S2 Fig). Cells were harvested by centrifugation in early stationary growth phase since in the open environment, phytoplankton populations are a mix of new cells and newly senescent cells.

### Pigment extraction

Pigments were extracted according to a new original [13] extraction protocol, which allowed an efficient, environment-friendly, non-damaging extraction of pigments, even those strongly linked to the thylakoid membranes [14]. Briefly, a pellet of 50.10^6^ algae cells was dispersed in 500 μL of ethanol (EtOH) and mixed with 0.728 g of 500 μm glass beads for 30 min at 30 Hz frequency in a mixer-mill (Retsch MM-400). The beads were then removed, the pigment extract centrifuged for 5 min at 4500 *g* at 4°C and the supernatant filtered on a 0.2 μM PTFE filter before immediate HPLC analysis.

### HPLC analysis

Pigment extracts were analysed using the SCOR/UNESCO reference method described by Van Heukelem et al. (2001) [13], with a slight modification. Pigments were separated on an Eclipse XDB-C8 (4.6 × 150 mm, 3.5 μm particle size, Agilent) column, using an Agilent HPLC-UV-DAD series 1200. The elution gradient consisted in a 30 min gradient from 95% to 5% solvent A (70:30 methanol, 28 mM aqueous TBAA pH 6.5). At the end of the run, an isocratic flow of 5% solvent A was maintained for 10 min. The flow rate remained at 1 mL/min during the run. Pigment elution was acquired at 436, 450, 405 and 665 nm. Wavelength 436 nm is used to look for all pigments, 450 nm is specific to carotenoids, 405 nm allow the visualization of chlorophyll degradation products and 665 nm is specific to chlorophyll *a* and its derivatives. All peak spectra were acquired from 210 to 800 nm (Agilent Diode Array Detector G1315D) and the ChemStation software (Agilent Technologies) was used to create a spectral library.

### Pigment identification

A chromatographic and spectral library was created from standard pigments provided by DHI lab products (Denmark). The pigments identified with a pure standard were: chlorophyll *b* (Chl *b*), chlorophyll *a* (Chl *a*), pheophorbide *a* methyl ester (Pheide *a* methyl ester), chlorophyllide *a* (Chlide *a*), chlorophyll *c*_*2*_ (Chl *c*_*2*_), chlorophyll *c*_*3*_ (Chl *c*_*3*_), pheophytin *a* (Phe *a*), astaxanthin (Asta), antheraxanthin (Anthe), lutein (Lut), 19’-hexanoylfucoxanthin (Hex-fuco), fucoxanthin (Fuco), 19’-but-fucoxanthin (19’-but-Fuco), 19’-hex-fucoxanthin (19’-hex-Fuco), violaxanthin (Viola), 9’-*cis*-neoxanthin (*c*-Neo), zeaxanthin (Zea), diadinoxanthin (Diadino), diatoxanthin (Diato), **β**,**ε**-carotene (**β**,**ε**-Car) and **β**,**β**-carotene (**ββ**-Car). Canthaxanthin (Cantha), dinoxanthin (Dino), chlorophyll *a* and *b* allomers (Chl *a* or *b* allo), peridinin (Peri), alloxanthin (Allo), peridinin isomer (Peri-iso), crocoxanthin (Croco) and prasinoxanthin (Pras) were identified according to DHI mix-108 and DHI mix-111 (DHI, Denmark). Standards were evaporated under nitrogen and solubilized in ethanol allowing comparison of spectral signatures, as samples were analysed in the same solvent. Pigments in microalgae extracts were identified by comparison of chromatographic and spectral data recorded with standard pigments, using ChemStation software (Agilent). Unidentified pigments were also stored in the spectral library. Identification of pigments was realised according to software retention time and match factor result. The software compared retention times and aligned absorption spectra (from 210 to 800 nm), to calculate a match factor comprised between 0 and 1000, representing the degree of similarity between spectra. The comparison of two spectra gives the match factor, which is defined as:

$$Match\;Factor=\frac{10^{3}\;\times {\left\{{\sum^{\;}}^{\;}x\;\times \text{ y }-\;\left(\frac{{\sum^{\;}}^{\;}x\;\times \;{\sum^{\;}}^{\;}y}{n}\right)\right\}}^{2}}{\left\{{\sum^{\;}}^{\;}x^{2}-\;\left(\frac{{\sum^{\;}}^{\;}x\;\times \;{\sum^{\;}}^{\;}x}{n}\right)\right\}\;\times \;\left\{{\sum^{\;}}^{\;}y^{2}\;-\;\left(\frac{{\sum^{\;}}^{\;}y\;\times \;{\sum^{\;}}^{\;}y}{n}\right)\right\}}$$

The *x* and *y* values are measured absorbance from the first and second spectrum respectively, at the same wavelength; *n* is the number of data points and ∑ the sum of the data. At the extremes, a match factor of 0 indicates no match whilst 1000 indicates identical spectra. In our case, values greater than 999 indicate that the spectra are similar. If the score value was between 995 and 999, a stringent alignment of spectra in the visible area was then checked (from 350 to 800 nm) and, if the match factor was higher than 999, the pigment was considered as similar and marked with an asterisk (*). All values below 995 indicate that the spectra are different. For very low abundance pigments, the match factor could be influenced by spectral noise level. In case of strong abundance of a well-known pigment, even if the score was slightly below 999, the pigment was identified according to the occurrence of the well-known pigment described in the species and marked with a cross (^†^). For each unidentified pigment which did not match a score >995, the spectrum was registered in the spectral library and annotated according to their specific retention time and spectral properties (*i*.*e*. carotenoid or porphyrin spectral signature).

All unidentified pigments were named according to their spectral signature and their elution order: Car(x) for carotenoid-like pigments, P(x) for porphyrin-like pigments and PCar(x) for pigments with both spectral properties and with a Red-Soret ratio higher than 5%.

Carotenoids and porphyrin band ratio were calculated according to [8]. Details for band ratio calculation are presented in supporting information S1 file for carotenoids (S1A Fig) and for porphyrins (S1B Fig).

### Determination of pigment communities among phytoplankton strains

The inter-strain comparison of pigment composition was studied and graphically represented using the open source Gephi communities’ network software (http://gephi.github.io/) as recommended in [15]. All strains and pigments (whether identified or not) were entered in the software and pigments were associated with their respective strains only by a qualitative link (presence of the pigment in the strain). The software grouped the strains together in communities sharing common pigments. Circles with a high diameter represent strains, while circles with a small diameter represent pigments. The validation of the communities was illustrated by the central circles of beta-carotene and chlorophyll *a* (including Chl *a* epimer), which were common to all strains. To make the results easier to read, microalgae belonging to the red, green and brown lineages are presented using their respective colours.

## Results

### Pigment composition of the 37 phytoplankton strains

The pigment chromatograms recorded at 436 nm are presented in Supporting Information S2 Fig. Chlorophyll *a* (and epimer) and **β,β**-carotene were common to all strains. In addition, each strain contained approximately 10 major and minor pigments, identified using the standard pigment database. All pigments detected among the biodiversity of the 37 strains were classified according to their HPLC retention times (Table 2). This analysis revealed that, in addition to the 26 standard pigments, 98 molecules corresponding to unidentified pigments or standards derivatives were present in the chromatograms. According to their spectral properties (presence or absence of absorption bands I, II and III), 57 pigments were classified as carotenoid derivatives (Car), 35 as porphyrin derivatives (P) and six pigments with spectral properties of both were named PCar. These unidentified pigments were named according to their elution order in the HPLC system. Table 2 summarizes the retention time, maximal absorption wavelengths, band ratio and occurrence in the strains for each pigment detected in the chromatograms. Pigment standards are written in italic characters and pigments characteristic of one strain or one species are written in bold characters. Pigments identified on the basis of their polarity and spectral absorption are indicated in brackets. The number of unidentified pigments may be in fact slightly inferior to 98, as the real number of chlorophyll allomers is probably inferior to those discriminated by our analysis, according to the slight variability of retention times and spectrum comparison. A high diversity of pigment spectra was observed and rules of identification and differentiation were created, allowing the creation of Table 2. Some pigments have very close retention times and band ratios but were not a sufficiently good match to be merged. We determined band ratio for chlorophyll and carotenoid. These results could be used to make comparisons among our results and with descriptions in the literature.

**Table 2:** Elution order of all pigments identified in microalgae strains. Maximum absorbance spectra in the eluent were detailed for porphyrins (P) and carotenoids (Car).

Peak N°	Short name	Retention time [min]	Porphyrin			Carotenoid				Occurrence in strains
			Soret [blue] I [nm]	Red II [nm]	Soret:Red	I [nm]	II [nm]	III [nm]	%III/II	
1	P1	6.6	432	622	9.67					Nsp
2	***Chl c***_***3***_	6.7	456	589	8.79					Eh*
3	Car1	6.8					467			At1; At2*; Am1; Am2
4	P2	7.3	446	631	8.33					Cmu; Ccp; Ctl; Oa1*; Ig*; Cg*; Pt; At1; At2
5	**P3**	7.7	441	630	6.73					Nsp
6	**P4**	8.1	434	628	5.83					Gs
7	**P5**	8.1	458	636	9.00					At1
8	**P6**	8.2	436	651	4.40					Cr
9	**P7**	8.2	434	629	6.13					Sg
10	***Chlide a***	8.4	432	666	0.98					Cb
11	**P8**	8.6	436	656	5.20					Cr
12	**P9**	8.8	440	631	6.56					Ot
13	*Chl c*_*2*_	8.9	447	633	7.90					Rs; Ctl; Cmu; Oa2; Eh; Tl; At1^†^; At2; Am2;
14	P10 (Chl *c*_*2*_ like)	8.9	447	634	7.53					Tp; Cg; Ig; Oa1; Cmi; Pt*
15	**P11**	8.9	445	666	6.01					Ccp
16	**P12**	8.9	440	666	2.06					Pt
17	**P13**	8.9	435	666	1.31					So
18	**P14**	8.9	436	666	1.30					Sg
19	**P15**	8.9	445	666	3.06					Cc
20	P16 (Chl c_1_ like)	9.2	444	633	6.48					Tp; Pt^†^; Cg; Ccp; Ctl; Cmu; Oa1; Oa2; Tl; Ig
21	**P17**	9.2	443	634	5.53					Sg
22	**P18**	9.2	445	633	9.2					Cc
23	P19	11.0	432	667	1.00					Pt; Sg
24	Car2	11.9					453			Ig; Tl
25	*Pheide a*	12.1	409	666	2.24					Gs
26	*Peri*	12.3					476			At1; At2; Am1; Am2
27	**P20**	12.8	410	665	1.97					Cr
28	*Peri-iso*	12.8					477			At1; At2; Am1; Am2
29	P21	13.8	431	665	1.00					Cb; Pt; Sg; Gs; So
30	Car3	14.1					470			Ctl; Nsp
31	**Car4**	14.1					468			At1
32	**PCar1**	14.2	422	666	9.12		470			Am2
33	**Car5**	14.6					449			Oa1
34	**Car6**	15.0					454	474	218	Ot
35	Car7	15.5					462			At1; At2; Am1; Am2
36	Car 8	15.5					440	467	48	Bn; Ts
37	**Car9**	15.5					442	468	81	Cr
38	Car 10	15.9					468			At1; At2; Am1; Am2
39	**Car 11**	16.3				418	441	470	103	Cr
40	Car12 (t-neo-like)	16.3				417	441	470	89	Bn; Ep; Ts; Ot; Cv; Ca; Dsp; Ds; Hp; So; Saa
41	**Car13**	16.7					484			Am1
42	*Fuco*	16.9					453			Tp; Pt; Cc; Cg; Ccp; Ctl; Cmi; Cmu; Nsp; Oa1; Oa2; Sg; Eh; Tl; Ig
43	Car14	17.5					479			At2; Am1; Am2
44	Car15 (Loro like)	17.5					445	472	41	Bn; Cr; Ts; So; Saa
45	*9-cis Neo*	17.6				413	436	465	86	Bn; Ep; Cr; Ts; Ot; Cv; Ca; Dsp; Ds; Hp; Cb; So; Saa
46	Car16	17.7					475			At1*; At2; Am1; Am2
47	Car17	17.9				425	447	476	62	At1; At2; Am1; Am2
48	***19-hex k Fuco***	17.9					448	469	85	Eh
49	***Pras***	18.4					455			Ot
50	*Viola*	18.8				416	439	469	89	Bn; Cr; Ig; Ts; Ot; Cv; Ca; Dsp; Ds; Hp; Cb; So; Saa
51	*Asta*	19.4					479			Cr; Saa
52	**Car18**	19.8					459			Ot
53	**19’-Hex-Fuco**	19.2				447	462	468	9	Eh
54	Car19 (*cis*-Fuco like)	20.2						443		Tp; Pt; Cc; Cg; Ccp; Ctl; Cmi; Cmu; Nsp; Sg; Tl; Ig; Oa1; Oa2
55	Car20	20.4				403	428	455	64	Am1; Am2; At1; At2
56	*Diadino*	20.6				422	446	475	54	Ep; Tp^†^; Pt; Cc; Cg; Ccp; Ctl; Cmi^†^; Cmu; Nsp; Oa1*; Oa2; Sg; Eh; Tl; Ig; Am1; Am2; At1; At2
57	Car21	20.8				411	434	462	68	Bn; Ts; Dsp*
58	**Car22**	20.9					458			Ep
59	**Car23**	21.5					440	466	55	Cr
60	**Car24**	21.5					442	467	42	Ts
61	Car25	21.5					443	469	22	Ot; Ca; Ds
62	**Car26**	21.5				415	437	464	82	Cv
63	Car27	21.5				417	438	465	70	Saa; Hp
64	*Anthe*	21.7					445	473	60	Dsp; Bn; Cb
65	**Car28**	21.9				419	439	468	37	Ep
66	**Car29**	22.1					471			Cr
67	*Allo*	22.1					452	479	32	Rs; Am1
68	**Car30**	22.4				421	441	470	67	Ep
69	**Car31**	22.4					452			At2
70	**Car32**	22.6				420	441	470	58	Ep
71	***Car33***	23.1				445	462	475	58	Rs
72	*Diato*	23.2					469	478	32	Tp; Cc; Cg; Ccp; Ctl; Cmi; Cmu*; Nsp; Oa1; Oa2; Sg; Eh; Tl; Ig; Ep*; Am1; Am2*; At1
73	**P22**	23.6	429	659	1.00					Gs
74	*Zea*	23.9					451	477	25	Bn; Cr; Cb; Cp; Ts*; Ot; Cv; Ca; Dsp; Ds; Hp; So; Saa; Rv; Pp; Gs; Cg*
75	*Lut*	24.2					445	472	60	Bn; Cr; Ts; Cv; Ca; Ds; Dsp; Hp; Cb; So; Saa
76	**Car34**	24.6					427	453	59	Ot
77	**P23**	24.8	438	688	5.01					Ts
78	Car35	24.8				420	438	465	41	Dsp; Ds; Hp; Cb; So; Saa; Cv
79	**PCar 2**	24.8	438	688	19.3	416	438	465	9	Ca
80	Car36	25.0					444	469	10	Cp; Gs; Rv; Pp*
81	*Cantha*	25.5					476			No presence in analyzed strains
82	Car37	25.8				419	439	467	61	Ts*; Hp*; So; Saa
83	Car38	25.9				419	440	467	59	Cv; Ca; Ds; Dsp;
84	Car39	26.0					445	471	31	Gs; Rv; Pp
85	**Car40**	26.5					461			Ep
86	Car41	27.6					446	473	37	Ts; Bn
87	P24	27.6	420	655	1.84					Cg*; Cmu; Nsp
88	P25	27.6	420	655	1.88					Ccp; Ctl
89	P26	27.7	456	635	1.84					Hp; Saa
90	**P27**	28.6	436	661	1.23					Bn
91	Car42	29.0					446	473	38	Bn; Cr; Ts
92	*Chl b*	29.7	468	651	2.80					Bn; Ep; Cr; Ts; Ot; Cv; Ca; Dsp; Ds; Hp; So; Saa; Cb
93	**Car43**	29.9					468			Tl
94	**Car44** (Croco like)	30.2					446	472	50	Rs
95	Chl *b*-epi	30.3	468	651	2.80					Bn; Ep; Ts; Ot; Cv; Ca; Dsp; Ds; So; Hp
96	**Car45**	31.0					445	471	25	Rv
97	P28 (chl *a* allomer)	31.1	420	655	1.78					Tp; Pt; Cc; Cg; Ccp; Ctl; Cmi; Cmu; Nsp; Oa1; Hp; Saa; At1; At2
98	**Car46**	31.2					446	472	50	Bn
99	Car47 (Cryp)	31.2					451	477	24	Gs; Rv; Pp
100	P29 (chl *a* allomer)	31.3	419	662	1.63					Pt*; Cc*; Ccp; Ot
101	**P30** (chl *a* allomer)	31.5	430	664	1.11					Ts
102	**P31** (chl *a* allomer)	31.5	432	665	1.10					Cv
103	P32 (chl *a* allomer)	31.5	422	664	1.20					Pt; Cc*; Ccp; Ctl; Ot
104	P33 (chl *a* allomer)	31.5	429	665	1.11					Nsp*; Oa1*; Cmi; Cmu*; Ca; Hp*; Saa; At1; At2
105	**Car48**	31.8					474			Tl
106	*Chl a*	32.3	431	665	1.00					All strains
107	P34	32.6	422	666	3.53					Nsp; Ccp
108	Chl *a* -epi	32.8	431	665	1.00					All strains with At1*; At2*; [excepted Eh]
109	**Car49**	33.1					472			Ep
110	**Car50**	33.4					425	451	44	Gs
111	**P35**	33.7	438	655	4.95					Cr
112	**PCar3**	34.5	445	666	6.67	416	445	474	56	At1
113	**PCar4**	34.5	415	666	5.16	415	444	474	37	Am1
114	**PCar5**	34.5	413	666	3.95	412	445	473	20	Am2
115	**Car51**	34.6					442	469	55	Ot
116	**Car52**	35.0				432	453	482	31	Dsp
117	**PCar6**	35.2	468	653	8.66	441	468	498	9	Ts
118	**Car53**	35.6				416	439	465	33	Cv
119	Car54	35.7				438	459	488	47	Ts; Ds; Dsp
120	Car55	35.8					444	467	9	Bn; Pt*; Cmu; Cb*; Cp; Gs; Ot; Cv; Ca*; Rv; Pp; Hp; Sg*; Ccp*; Ctl*; Nsp*; Oa1*; Oa2*
121	**βε***-Car*	36.4					444	472	56	Rs; Ts; Cv; So; Cb; Cr
122	**ββ***-Car*	36.5					450	475	15	All strains
123	Car56	37.1					440	466	28	Cp; Dsp; So; Cb; Ts; Bn
124	**Car57**	37.1					442	466	11	Gs

Pigment standards are written in italic characters. Pigment characteristics of one strain or several strains within a particular species are written in bold characters. Strains marked with an asterisk cross (^†^) indicates that the pigment is abundant and fully characterized in literature. Strains marked with an asterisk (*) have a match factor of 999 in visible spectra.

Among the uncharacterized pigments, six could be identified on the basis of their polarity (retention time), spectral properties and by descriptions in the literature of ancillary pigments in strains. They corresponded to Chl *c*_*1*_ like (P16) [16,17], *t*-neoxanthin (Car12) [18] loroxanthin like (Car15) [19,20], crocoxanthin like (Car44) [21], **β**-cryptoxanthin like (Car47) [22,23] and *cis*-fucoxanthin like (Car19) [17,24]. P28 to P33 were identified as Chl *a* allomers, as they corresponded to minor pigments with polarity and maximal absorption wavelengths very close to Chl *a*, but eluting a few seconds before Chl *a* [25]. This is also the case for P26, which corresponds to a Chl *b* allomer. All pigments identified were checked against literature descriptions with the same analytical method [13,26]. Without validation by mass spectrometry analysis, unidentified Car or P designating the pigment were instead used for putative identification.

### Detection of photoprotective pigments

As expected, the carotenoids involved in the xanthophyll cycles, ensuring thermic dissipation of high irradiance energy, were different between the red, green and brown lineages. Accumulation of zeaxanthin and absence of antheraxanthin and violaxanthin were observed in the Rhodophyceae (*Pp*, *Rv* and *Gs*), Glaucophyceae (*Cp*) and Charophyceae (*Cb*). Diadinoxanthin and diatoxanthin were detected in the Bacillariophyceae (*Sg*, *Cc*, *Cg*, *Ccp*, *Ctl*, *Cmi*, *Cmu*, *Nsp*, *Oa1*, *Oa2*, *Pt* and *Tp*) and Haptophyta (*Tl*, *Ig* and *Eh*). In some cases, diadinoxanthin was detected in the absence of diatoxanthin, indicating the total conversion of diatoxanthin into diadinoxanthin in our relatively low light culture conditions. Conversely, it is important to remember that diatoxanthin increases as cells become less healthy. Zeaxanthin/antheraxanthin/violaxanthin were detected in the chlorophytes *Cb* and *Dsp* and in the Chlorarachniophyceae *Bn*. Zeaxanthin/violaxanthin in absence of antheraxanthin were also detected in the other chlorophytes analysed, and in the Chlorarachniophyceae *Cr*. The presence of zeaxanthin and violaxanthin indicated the possibility for these strains to synthesize antheraxanthin, and its absence indicated a total conversion into zeaxanthin or violaxanthin in our culture conditions. Isochrysidaceae (*Ig*) and Skeletonemale (*Cg*) combined a diatoxanthin / diadinoxanthin photoprotective system with the ability to synthesize zeaxanthin, antheraxanthin and/or violaxanthin because of the conservation of an ancestral gene [27].

### Presence of pigments currently used as chemotaxonomic markers

Among the 124 HPLC peaks analysed in all phytoplankton strains, 63 were strain-characteristic and six were species-characteristic (shared by several strains in one considered species, in our case dinoflagellates). We thus detected 63 pigments (Table 2. bold characters) that could be used as stringent specific chemotaxonomic markers to discern a strain or species over the 37 studied. Six of these 63 pigments corresponded to standard pigments. Chl *c*_*3*_, 19’-hexanoyloxy fucoxanthin and 19-hexanoyl-k-fucoxanthin pigments indicated the presence of *Emiliania huxleyi* SAG33.90, prasinoxanthin indicated the presence of the Prasinophyte *Ostreococcus tauri* H95 and pheophorbide *a* occurred in *Galdieria sulphuraria*. Pheide *a* usually occurs in senescent algae, while in our case all the cultures were in early stationary phase of growth but cultivated in acidic condition, at pH 2, which could explain the loss of Mg and the phytyl-chain of Chl *a*. Moreover, the very low detection of pheophorbide *a* (Pheide *a*) among the 37 strains studied confirmed the quality of our fresh pigment extraction process, that limited Chl *a* damage. Chlorophyllide (Chlide *a*) is present in *Cb* but cannot be used as a chemotaxonomic marker because this molecule participates in the turnover of chlorophyll *a*. Chlide *a* is potentially distributed universally in microalgae.

S1 Table lists the pigments currently used as chemotaxonomic markers for phytoplankton strains, species or classes, according to the classification proposed by Wright and co-workers [28]. The detection of a chemotaxonomic marker unambiguously signals the presence of a strain in a sample, but its absence should not be interpreted as an absolute demonstration of the absence of a strain, as light and nutrient conditions can silence pigment expression.

Canthaxanthin is described as a significant chemotaxonomic marker but not always present for the Cyanophyceae CYANO-1 class and may be present in Eustigmatophyceae EUSTIG-1 and Dinoflagellate Chloroplast type-1 classes. As we did not detect canthaxanthin, we concluded that no strain was able to synthesize canthaxanthin. The light conditions in our experiments were not favourable to its biosynthesis, as canthaxanthin is a secondary carotenoid, involved in algal photoprotection. Astaxanthin is described as a chemotaxonomic marker for the Chlorophyceae (CHLORO-1) and Trebouxiophyceae classes. Accordingly, we identified it in *Scenendesmus acutus f*. *alternans* UTEX72, and in the Chlorarachniophyceae *Chlorarachnion reptans* SAG26.87. No astaxanthin was detected in other Chlorophyta strains, suggesting that the light irradiance was too low to trigger astaxanthin biosynthesis in these strains [29]. Antheraxanthin is used as a chemotaxonomic marker for the Chrysophyceae CHRYSO-1 and Eustigmatophyceae EUSTIG-1 classes and belongs to the zeaxanthin/antheraxanthin/violaxanthin photoprotection system. None of the strains studied here belong to these classes but some belong to the Chlorophyta species with the xanthophyll cycle photoprotection system. Thus, we identified this pigment (as minority or trace pigment) in *Dunaliella* sp. CCAP19/19. We also detected Antheraxanthin in *Closterium baillyanum* SAG50.89 (Closteriaceae) and *Bigelowiella natans* CCMP243 (Chlorarachniophyceae). Pigment patterns in Dinophyta were described by Zapata *et al*. [30]. Here, we analysed four strains with a chloroplast known as dinoflagellate chloroplast type-1. These four strains contained common carotenoids Car1, Car7 and Car10 with lambda max respectively at 467, 462 and 468 nm in our system. Peridininol and dinoxanthin are described as markers of dinoflagellate chloroplast type-1. Car1 could be identified as Peridininol but not enough clues are available to confirm it. Eight haptophyte pigment types were defined by Zapata [31]. *Emiliania huxleyi* SAG33.90 belongs to haptophyte pigment type-6, its characteristic pigments are Chl *c*_*3*_, 19-hex-k fucoxanthin and 19’hex-fucoxanthin. Other haptophytes analysed, *Ig* and *Tl*, did not exhibit such stringent markers.

### Analysis of phytoplankton pigment communities and detection of characteristic pigments

**Fig 1:**
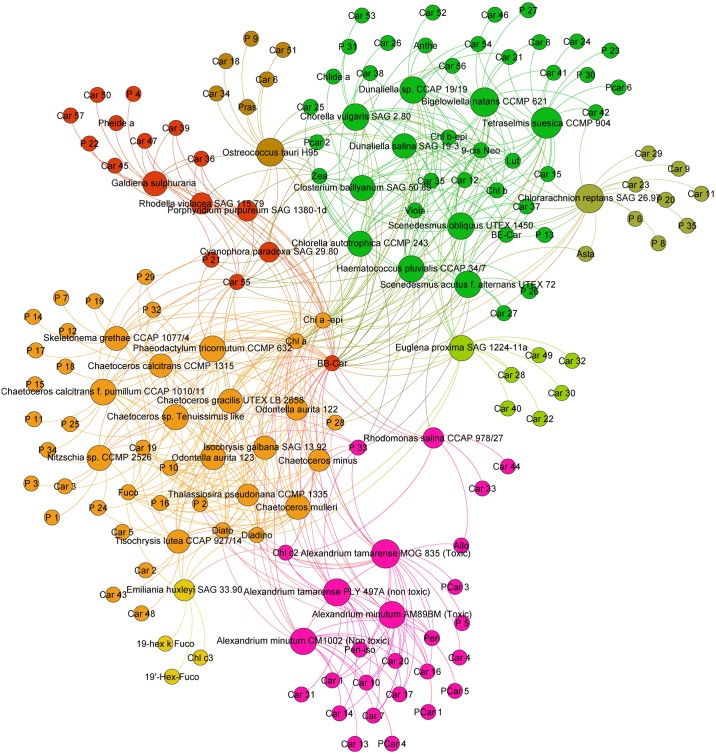
Pigment communities among the 37 microalgae strains studied.

We performed a community analysis to highlight the taxonomic clusters defined by the commonly shared pigments in the diverse phytoplankton strains. Fig 1 presents the pigment communities obtained for all strains. Strains clustered together into four major communities that correspond to the green, red, brown lineages and Dinophyceae focus. The cluster only shows a link between algae and pigment without taking into account the quantity of pigment. This result confirmed that the common sharing of photoprotective pigments, respectively zeaxanthin in the red lineage, violaxanthin/antheraxanthin/zeaxanthin in the green lineages and diadinoxanthin/diatoxanthin in brown strains, is a major relevant taxonomic key to classify the strains on the basis of their pigments. The graphical analysis also confirmed that in addition to the common photosynthetic and photoprotective pigments, most strains contain characteristic pigments. The graphical representation allows a fast identification of these pigments, as they cluster in the most external sides of the Gephi figure, and are connected to their productive strain by a unique link. Using the Gephi representation, the number of pigment characteristics of one strain was also very easy to determine visually. For example, *Ostreococcus tauri* H95 contains five characteristic carotenoids (Car6, Car18, Car34, Car51 and Pras) and one characteristic porphyrin (P9), while *Emiliania huxleyi* SAG33.90 contains two characteristic carotenoids (19-hex-k-fuco, 19’-hex-fuco) and one characteristic chlorophyll (Chl *c*_*3*_).

The Gephi analysis reveals that strains cluster together into four major communities corresponding to the green, red, brown lineages and a Dinophyceae clustering. Strain-characteristic pigments cluster at the edges of Fig 1, allowing a fast count and determination.

In the middle of the figure, key photosynthetic and photoprotective pigments are represented *i*.*e*., Chl *a*, its epimer and **ββ**-Car. In the yellow-orange group, Diato, Diadino and Fuco groups belong to the brown-yellow algae lineage. The green lineage is close to Chl *b* and its epimer, Viola, 9-*cis*-Neo and Lut. Zeaxanthin, because of its accumulation in red algae, is at the frontier between red lineage and green lineage. Dinoflagellates form a group with the presence of peridinin and its epimer.

As a second step, we separated the clusters, according to xanthophyll cycle, in order to perform a limited Gephi analysis. The aim is to obtain a better resolution for strain-characteristic pigments and a fast pigment determination shared by a short number of strains within one or several species.

#### Red lineage

**Fig 2:**
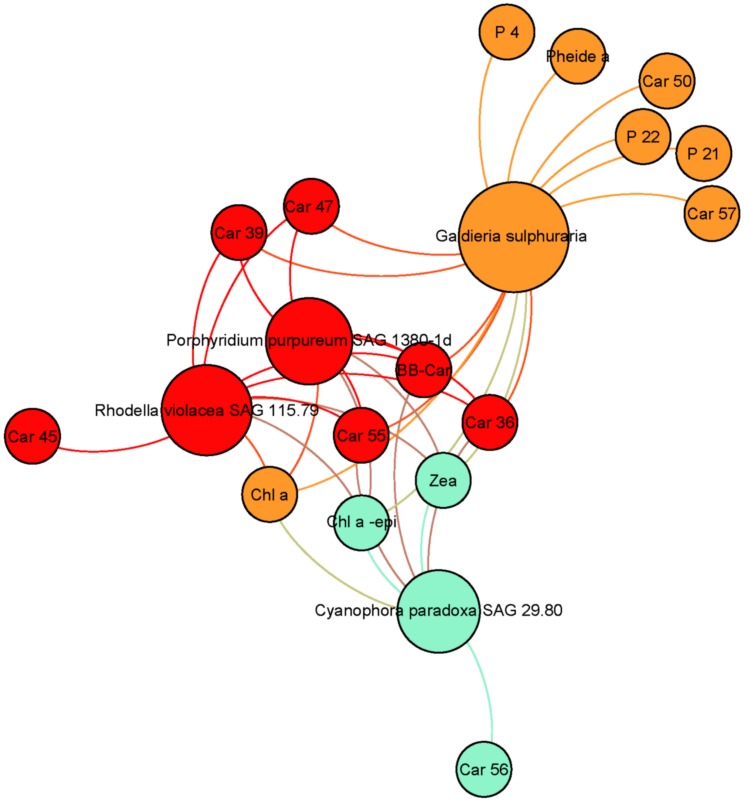
Pigment communities among red strains accumulating zeaxanthin for photoprotection (Rhodophyta, Glaucophyta). The core of Fig 2 groups together photosynthetic pigments, with Chl *a* and its epimer, **ββ**-Car and Zea. Two other pigments included are Car55, which has an elution very close to **ββ**-Car, and Car36. Car36 is a key ancillary pigment binding Rhodophyta and Glaucophyta. Presence of these pigments in oceanographic analyses can indicate the presence of the corresponding phyla.

Fig 2 presents the pigment communities and contents among the red lineage. Rhodophytes (*Pp*, *Rv*, *Gs*) and glaucophytes (*Cp*) are characterized by the common synthesis of Zea, Chl *a* epimer, Car36 and Car39. It should also be noted that rhodophytes and glaucophytes strains synthesize phycobiliproteins, which are not presented in this study. Car47 was identified as **β**-cryptoxanthin and the presence of this pigment in three strains accumulating zeaxanthin was coherent because **β**-cryptoxanthin is the biosynthetic intermediate between **β**-carotene and zeaxanthin. Its presence in *Porphyridium purpureum* SAG1380-1d was also independently confirmed by a high-resolution mass spectrometric analysis [32]. In addition, *Galdieria sulphuraria* contained 1 characteristic carotenoid (Car50), and the three characteristic porphyrins P4, P21 and P22. Pheide *a* is only present in *Gs* in the red lineage but it cannot be considered as a marker because this pigment is a degradation pigment. This fact cannot be explained by the extraction process (gentle method) but by the acid culture conditions of this strain, which catabolises this kind of chlorophyll degradation product. Car45 was characteristic of *Rv*.

Synthesis of astaxanthin and lutein was previously reported in *Gs* in autotrophic conditions [33] but no traces of either of these pigments were detected in our study. Absence of these two pigments indicates that the relatively low light conditions in our experiments were not favourable to their biosynthesis in this strain. Nevertheless, *Gs* has a porphyrin (P21) in common with green lineage algae, this is also the case of a carotenoid Car57 in *Cp*.

#### Green lineage

**Fig 3:**
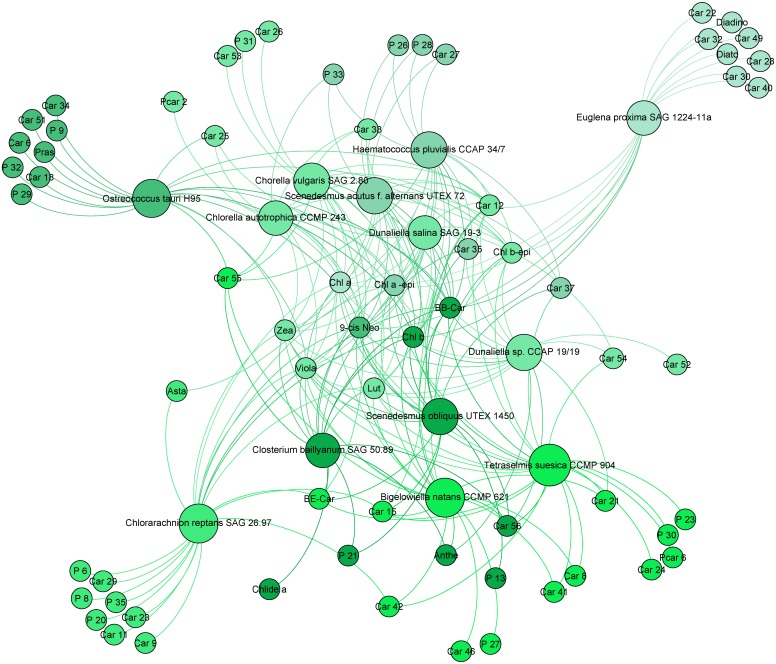
Pigment communities among green strains, using the zeaxanthin/antheraxanthin/violaxanthin cycle for photoprotection (Charophyta, Chlorophyta and Cercozoa).

Fig 3 presents the pigment composition of strains containing a Viola/Anthera/Zea cycle. *Ep* was studied with the green lineage as its endosymbiont contains Chl *b*. All green strains contained Chl *b*, Chl *b* epimer and Neo. Lutein was also present in all strains except *Ot* and *Ep*. **βε**-Car is a lutein precursor, its occurrence in some strains is explained by the pathway suggested by Takaichi [18].

Car12, identified as *t*-neoxanthin-like, was common to nearly all chlorophytes and the chlorarachniophyte *Bn*, but excluded *Cr*. This pigment is a close metabolic derivative from neoxanthin and 9-*cis*-violaxanthin. Car12, which is present in our chlorophyte strains, is quite a good marker for this phylum. Moreover, most strains contain one to eight characteristic pigments: Car52 in *Dsp*; Car46 and P27 in *Bn*; Car24, PCar6, P23 and P30 in *Ts*; Car26, Car53 and P31 in *Cv*; P13 in *So*; Car9, Car11, Car23, Car29, P6, P8, P20 and P35 in *Cr*; and PCar 2 in *Ca*. In our case, PCar 2 seems to be a co-elution of a porphyrin and a carotenoid. The carotenoid has the same lambda max as Car35 present in *Cv* and other green algae analysed.

Strain *Cr* contained characteristic porphyrin derivatives (P6, P8, P20 and P35) and four characteristic carotenoids (Car9, Car11, Car23 and Car29). They may belong to the species *Nitzschia curvilineata* SAG48.91, whose addition to the culture medium was required to feed *Cr*. Its presence in the *Cr* culture was minor but necessary for the normal growth of this strain. However, no fucoxanthin, diatoxanthin or diadinoxanthin (major markers from diatoms such as *Nitzschia curvilineata*) were detected in the analysis of the culture of *Cr*. As major markers from diatoms could not be detected, we assume that no minor pigments from *Nitzschia curvilineata* could be detected in the sample *Cr*. Then, a taxonomic curiosity with *Cr* analysis occurred. This phylum (Cercozoa) belongs to the green lineage (photoprotection cycle Zea/Anthe/Viola). Pigments common with Chlorophyta strains were detected but none from the photoprotection cycle (Diato/Diadino) were detected in the strains *Cr* and *Bn*. However, this phylum is still classified in the Chromista kingdom. By taking into account only pigment criteria, we think that Chlorarachniophyceae should belong to the Plantae kingdom. *Closterium baillyanum* is the only representative of the Charophyta phylum in this study. Chlide *a*, P21, 9-*cis*-Neo, Viola/Zea, Anthe, Lut, **βε**-Car and Chl *b* were detected in this strain. These pigments confirm that this phylum is in the correct position in the chemotaxonomic tree. Astaxanthin is not present in most chlorophytes (except *Saa* and *Cr*). Asta is considered as a marker but it cannot be used in all conditions. Our culture conditions were not favourable to biosynthesis of this marker. This photoprotective pigment is only highly expressed in stress conditions, as is the case for astaxanthin esters in *Hp*.

If we refer to the Zea/Anthe/Viola xanthophyll cycle in green algae, the low light culture conditions allow the presence of these three pigments. Nevertheless, only *Dsp*, *Bn* and *Cb* contain Anthe with a retention time of 21.5 min. Other algae from the green lineage contain pigments having a 21.5 min retention time but their spectral properties did not properly match with Anthe identification. This is the case for Car23 in *Cr*; Car24 in *Ts*; Car25 in *Ot*, *Ca* and *Ds*; Car26 in *Cv*; and Car27 in *Saa* and *Hp*. The low abundance of these peaks may explain the pigment diversity described, which could belong to Anthe derivatives. Anthe was not detected in *Ep* which contained six characteristic carotenoids Car22, Car28, Car30, Car32, Car40 and Car49 with the presence of Diato-Diadino which highlights the specificity of the Euglena lineage. The latter are also represented in the following figures. According to their retention time and spectra, Car30 and Car32 could be identified as vaucheriaxanthin esters.

#### Brown lineage and Dinophyceae clade

**Fig 4:**
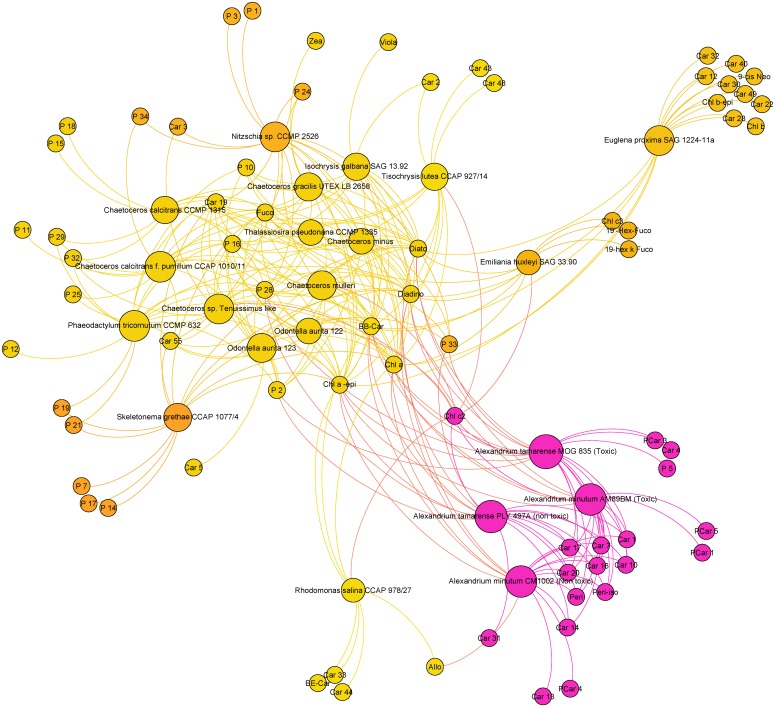
Pigment communities among strains containing a Diato/Diadino cycle (Haptophyta, Ochrophyta, Dinophyta and Euglenozophyta).

**Fig 5:**
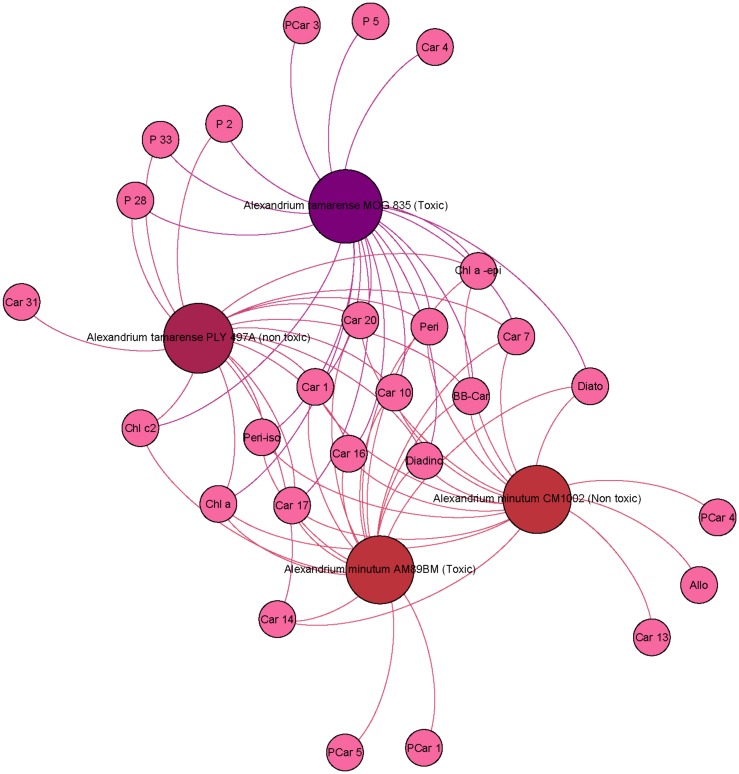
Pigment communities among *Alexandrium* strains.

Figs 4 and 5 present the pigment communities and content among the strains containing a Diato/Diadino cycle.

All strains are grouped near Chl *a* and its epimer, **ββ**-Car, Diato and Diadino. Diatoms and Haptophyta are grouped near Fuco and Car19 (9-c*is*-fucoxanthin like). These microalgae have a strong diversity of chlorophyll pigment types (small circles at the periphery of the figure). Specific porphyrin markers are present in *Sg* (P7, P14 and P17), *Nsp* (P1 and P3), *Ccp* (P11), *Cc* (P15 and P18) and *Pt* (P12). P28 is also near the centre of the figure suggesting its closeness with Chl *a* biosynthesis. High chlorophyllide and pheophorbide contents, resulting from the hydrolysis of chlorophylls and pheophytins by active chlorophyllases, are usually described in bacillariophytes [34,35]. Accordingly, some porphyrin derivatives were exclusively found in certain Bacillariophyceae strains among which they were shared. P2 was detected in *Cmu*, *Ccp*, *Ctl*, *Oa1*, *Pt* and *Cg* (6 Bacillariophyceae) in *At1* and *At2* (dinoflagellates) and also in *Ig* (Coccolithophyceae). P24 and P25 have very close properties but it was not possible to merge these pigments using the matching factor. They are present in *Cg*, *Cmu* and *Nsp* for P24, and P25 was characteristic of two *Chaetoceros* strains (*Ccp* and *Ctl*).

A Chl *c*_*2*_ like pigment (P10), exhibiting a retention time very close to that of Chl *c2* and also a close absorption spectrum (score >995) was also detected in several Chromista strains (Fig 4). This spectral variation may be linked with the co-elution with Chl *c1* (P16) at different levels of expression. Other porphyrin derivatives were shared between strains from various classes. P29 and P32 were present in the chlorophyte *Ot*. P33 was also present in *Ca*, *Hp* and *Saa*.

Chl *c*_*2*_ is a key marker in the Chromista, as well as in dinoflagellates and Cryptophyta. Presence of Allo in *Rs* is a known taxonomic marker, but it is was not described in Dino-1 plastid type. Fig 4 reveals that Allo is present in *Am1*, a result not previously observed in literature because it is a marker of Dino plastid type-5 [30]: most photosynthetic dinoflagellates contain a chloroplast with peridinin as the major carotenoid. Chloroplasts from other algal lineages have been reported, suggesting multiple plastid losses and replacements through endosymbiotic events. The pigment composition of 64 dinoflagellate species (122 strains) was analysed by high-performance liquid chromatography. In addition to chlorophyll (chl) *a*, both Chl *c*_*2*_ and divinyl protochlorophyllide occurred in chl c-containing species. Chl *c*_*1*_ co-occurred with Chl *c*_*2*_ in some peridinin-containing (*e*.*g*., *Gambierdiscus* spp.) and fucoxanthin-containing dinoflagellates (*e*.*g*., *Kryptoperidinium foliaceum*). Chl *c*_*3*_ occurred in dinoflagellates whose plastids contained 19’-acyloxyfucoxanthins (*e*.*g*., *Karenia mikimotoi*). Chl *b* was present in green dinoflagellates (*Lepidodinium chlorophorum*). Based on unique combinations of chlorophylls and carotenoids, six pigment-based chloroplast types were defined: Type 1: peridinin/dinoxanthin/chl *c*_*2*_ (*Alexandrium minutum*); Type 2: fucoxanthin/19’-acyloxy fucoxanthins/4-keto-19’-acyloxy-fucoxanthins/gyroxanthin diesters/chl c_2_, c_3_, monogalactosyl-diacylglycerol-chl *c*_*2*_ (*Karenia mikimotoi*); Type 3: fucoxanthin/19’-acyloxyfucoxanthins/gyroxanthin diesters/chl *c*_*2*_ and *c*_*3*_ (*Karlodinium veneficum*); Type 4: fucoxanthin/Chl *c*_*1*_ and *c*_*2*_ (*K*. *foliaceum*); Type 5: alloxanthin/Chl *c*_*2*_/phycobiliproteins (*Dinophysis tripos*); Type 6: neoxanthin/violaxanthin/a major unknown carotenoid/Chl *b* (*Lepidodinium chlorophorum*). While plastids with peridinin, and probably those with Chl *b*, originated from secondary endosymbiosis, the other chloroplast types were obtained through tertiary endosymbiosis. Chloroplast types corresponded with evolutionary lineages within dinoflagellates. Caution must be observed when only peridinin is used for tracking photosynthetic dinoflagellates in field samples. The additional marker pigments offer oceanographers greater power for detecting dinophytes in mixed populations [32].

All Cercozoa, Haptophyta and Ochrophyta strains contained Fuco. Fuco ester derivatives were likely to be present in these strains [36], as confirmed by the detection of 19’-but-fuco and 19’-hex-fuco in *Eh*. It is most likely that other Fuco derivatives, including the 4-keto forms of both Fuco esters, corresponded to unidentified carotenoids detected in the chromatograms. *Tl* contained two characteristic carotenoids, Car43 and Car48. Car2 was common to *Ig* and *Tl*, the two Isochrysidaceae.

#### Dinophyceae

For Fig 5, we selected two toxic strains (*At1* and *Am2*) and two non-toxic strains (*At2* and *Am1*). Detection of Peri and Peri-iso in the four strains confirmed that they belonged to the Dino-1 group defined by [28], but these pigments could not explain the toxicity. The conclusion is the same for Car1, Car7, Car10, Car16, Car17 and Car20. These pigments were present in all *Alexandrium* strains (*At1*, *At2*, *Am1* and *Am2*) and in no others. These pigments are good markers of the genus *Alexandrium*. Phycotoxin analyses (P. Lassus, personal communication) showed the toxins of *At1* are GTX4, C2, NeoSTX and GTX1 (by order of decreasing abundance), while the toxins of *Am2* are GTX3, C2 and GTX2. Pigments can be toxic sometimes [37] but in the case of *At1* and *Am2*, toxicity seems to be held by molecules other than carotenoids. Car4 was present only in toxic *A*. *tamarense* (*At1*), while PCar1 (similar retention time and maximum wavelength) was detected only in toxic *A*. *minutum* (*Am2*). Three strains contained characteristic porphyrin-carotenoid pigments, namely PCar4 in *Am1*, PCar1 and PCar3 in *At1*, PCar4 in *Am1*, PCar5 in *Am2*. It is important to note that PCar1, PCar3, PCar4 and PCar5 are uncommon molecules with spectral properties from both porphyrins and carotenoids.

Table 3 summarizes the pigment combinations that, according to the results of our study, can be used as stringent chemotaxonomic markers to discern one strain or one species over the 37 algae studied. Pigments in bold indicate the strain-characteristic pigments whose detection gives high robustness to the identification of the strain in the sample.

**Table 3:** Key pigments to identify the 37 phytoplankton strains.

Phylum	Class	Family	*Strain code*	Short name	Characteristic pigment combination	Taxonomic group
**Eukaroyte Plantae**						
**Rhodophyta**	Porphyridiophyceae	Porphyridiaceae	*Porphyridium purpureum* **SAG 1380-1d**	Pp	No stringent combination	RHODO-1
	Rhodellophyceae	Rhodellaceae	*Rhodella violacea* **SAG 115.79**	Rv	**Car45**	RHODO-1
	Cyanidiophyceae	Galdieriaceae	*Galdieria sulphuraria*	Gs	**P4**—Pheide a—**P22**—**Car50—Car57**	RHODO-1
**Glaucophyta**	Glaucophyceae	Glaucocystaceae	*Cyanophora paradoxa* **SAG 29.80**	Cp	No stringent combination	GLAUCO-1
**Charophyta**	Conjugatophyceae	Closteriaceae	*Closterium baillyanum* **SAG 50.89**	Cb	No stringent combination	CHLORO-1
**Chlorophyta**	Chlorophyceae	Scenedesmaceae	*Scenedesmus acutus f*. *alternans* **UTEX 72**	Saa	No stringent combination	CHLORO-1
			*Scenedesmus obliquus* **UTEX 1450**	So	**P13**	CHLORO-1
		Haematococcaceae	*Haematococcus pluvialis* **CCAP 34/7**	Hp	No stringent combination	CHLORO-1
		Dunaliellaceae	*Dunaliella* sp. **CCAP 19/19**	Dsp	**Car52**	CHLORO-1
			*Dunaliella salina* **SAG 19–3**	Ds	No stringent combination	CHLORO-1
	Trebouxiophyceae	Chlorellaceae	*Chlorella autotrophica* **CCMP 243**	Ca	**PCar2**	TREBUXIO-1
			*Chorella vulgaris* **SAG 2.80**	Cv	**Car26**—**Car53—P31**	TREBUXIO-1
	Mamiellophyceae	Bathycoccaceae	*Ostreococcus tauri* **H95**	Ot	**P9—Car6—Pras—Car18—Car34**—**Car51**	PRASINO-3A
	Chlorodendrophyceae	Chlorodendraceae	*Tetraselmis suecica* **CCMP 904**	Ts	**Car24—P23—P30**—**PCar6**	PRASINO-2A
**Eukaryote Chromista**						
**Cercozoa**	Chlorarachniophyceae	Chlorarachniaceae	*Chlorarachnion reptans* **SAG 26.97**	Cr	**P6**—**P8**—**P20**—**Car9—Car11**—**Car23**—**Car29**—**P35**	CHLORARAC-1
			*Bigelowiella natans* **CCMP 621**	Bn	**P27—Car46**	CHLORARAC-1
**Haptophyta**	Coccolithophyceae (Prymnesiophyceae)	Isochrysidaceae	*Isochrysis galbana* **SAG 13.92**	Ig	No stringent combination	HAPTO-3
			*Tisochrysis lutea* **CCAP 927/14**	Tl	**Car43—Car48**	HAPTO-3
		Noelaerhabdaceae	*Emiliania huxleyi* **SAG 33.90**	Eh	**Chl *c3*—19-hex-k-Fuco—19’-hex-Fuco**	HAPTO-6
**Bacillariophyta**	Mediophyceae	Thalassiosiraceae	*Thalassiosira pseudonana* **CCMP 1335**	Tp	No stringent combination	DIATOM-1
		Skeletonemataceae	*Skeletonema grethae* **CCAP 1077/4**	Sg	**P7—P14—P17**	DIATOM-1
		Eupodiscacea	*Odontella aurita* **123**	Oa1	**Car5**	DIATOM-1
			*Odontella aurita* **122**	Oa2	No stringent combination	DIATOM-1
		Chaetocerotaceae	*Chaetoceros mulleri*	Cmu	No stringent combination	DIATOM-1
			*Chaetoceros minus*	Cmi	No stringent combination	DIATOM-1
			*Chaetoceros* sp. *Tenuissimus* like	Ctl	No stringent combination	DIATOM-1
			*Chaetoceros calcitrans f*. *pumillum* **CCAP 1010/11**	Ccp	**P11**	DIATOM-1
			*Chaetoceros calcitrans* **CCMP 1315**	Cc	**P18**	DIATOM-1
			*Chaetoceros gracilis* **UTEX LB 2658**	Cg	No stringent combination	DIATOM-1
	Bacillariophyceae	Bacillariaceae	*Nitzschia* sp. **CCMP 2526**	Nsp	**P1—P3**	DIATOM-3
	Bacillariophyceae incertae sedis	Phaeodactylaceae	*Phaeodactylum tricornutum* **CCMP 632**	Pt	**P12**	DIATOM-1
**Cryptophyta**	Cryptophyceae	Pyrenomonadaceae	*Rhodomonas salina* **CCAP 978/27**	Rs	**Car33**—**Car44**	CRYPTO-1
**Eukaryote protozoa**						
**Miozoa**	Dinophyceae	Goniodomataceae	*Alexandrium tamarense* **MOG 835** (Toxic)	At1	**P5—PCar1—PCar3**	DINO-1
			*Alexandrium tamarense* **PLY 497A** (non toxic)	At2	No stringent combination	DINO-1
			*Alexandrium minutum* **CM1002** (Non toxic)	Am1	**Car13—PCar4**	DINO-1
			*Alexandrium minutum* **AM89BM** (Toxic)	Am2	**PCar5**	DINO-1
**Euglenophyta**	Euglenophyceae	Euglenaceae	*Euglena proxima* **SAG 1224-11a**	Ep	**Car22—Car28—Car30—Car32—Car40—Car49**	EUGLENO-1

**Fig 6:**
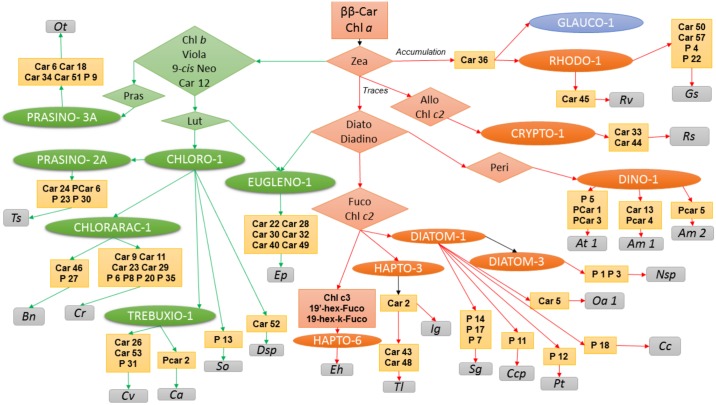
Dichotomous key for microalgae strain identification by pigment composition. This dichotomous key reveals that stringent identification of strains is possible according to pigment composition results presented in this paper.

The resolution of the ancillary pigment in order to identify strain could be solved as a dichotomy key as represented in Fig 6. Colours indicate the different taxonomic lineages and outsider phyla in terms of pigmentation.

All strains contain Chl *a* and **ββ**-Car. Zea is also present in all strains but its quantity depends on the different lineages. Accumulation of Zea, in absence of other photoprotective pigments, is observed in Rhodophyta or Glaucophyta. Traces of Zea could be observed in Chromista. Its presence in the xanthophyll cycle is linked with the green lineage. The amount of Zea is a major decisional element for stringent identification of plastid types. According to Table 3 and Fig 6, 24 algae strains could be identified according to ancillary pigment.

In the red lineage, Car36 is a key pigment allowing the identification of Rhodophyta and Glaucophyta. For a stringent identification *Rv* could be identified via Car45 and *Gs* via Car50, Car58, P4 and P22. The *Rs* (Cryptophyta) pigment composition reveals specific pigments (Car33, Car44) and the brown lineage/Dinophyceae clustering (Chl *c*_*2*_). Allo is a carotenoid previously described to be a marker of Cryptophyta. Moreover, we have to notice Cryptophyceae class produces phycobiliproteins as well as Porphyridiophyceae, Rhodellophyceae, Cyanidiophyceae, Glaucophyceae and cyanobacteria. This phylum is derived from the red lineage, but actually classified in the Chromista kingdom. All other Chromista lost their ability to synthesize Phycobiliproteins in favour of the light harvesting complex with a Diato/Diadino xanthophyll cycle. Bacilliariophyta and Haptophyta have Fuco and Chl *c*_*2*_. Among three analysed haptophytes, *Eh* was the only strain showing Chl *c*_*3*_. *Ig* and *Tl* have Car2 in common. *Tl* could be discriminated with specific Car43 and Car48. In Bacilliariophyta (diatoms), porphyrin biodiversity generates a strong difference between strains. Indeed, *Sg*, *Nsp*, *Ccp*, *Pt* and *Cc* had unique porphyrins. Moreover, Car19 is a common pigment in the Mediophyceae class and strain *Pt* belonging to Bacillariophyceae incertae sedis. This carotenoid could be used a group marker as well as Car2, which is a common pigment for both Isochrysidaceae analysed (*Ig* and *Tl*). The dinoflagellates analysed have specific pigments mixing characteristics of porphyrins and carotenoids (PCar1,3,4, and 5), allowing for easy identification. Peri and Peri-iso are well known to be present in dinoflagellates. In the four Alexandrium strains analysed, Car1, Car7, Car16, Car17, and Car20 are common pigments for this Genus.

Pigment composition of the Euglenophyceae representative (*Ep*) showed pigments in common with the green lineage (Car12, 9-*cis* Neo, Chl *b* and Chl *b*-epi) whilst some other pigments are characteristic of the brown lineage (Diadino and Diato). This typical signature should be compared to the endosymbiosis processes during its evolution. Car22, Car28, Car30, Car32, Car40 and Car49 are all markers of the Euglenophyceae strain (*Ep*).

The green lineage is characterized by Chl *b*, 9-*cis* Neo Car12 and Lut. Among the six strains belonging to CHLORO-1 two could be identified easily: *So* with P13 and *Dsp* with Car52. In TREBUXIO-1 three specific pigments characterize *Cv* and one *Ca*. Chlorarachniophyceae presented two and seven specific markers for *Bn* and *Cr*, respectively. Not all Prasinophyceae chloroplast types contains Pras. This is the case for *Ts* which, moreover, has four specific pigments (Car24, PCar6, P23, P30). *Ot* belongs to the PRASINO-3A plastid type, which contains Pras and five specific pigments (Car6, Car18, Car34, Car51 and P9).

This overview of pigment-based taxonomic clusters allows a fast determination of strain-specific and shared pigments. These findings support the hypothesis that some minor photoprotective and photosynthetic pigments can constitute tools to cluster strains.

## Discussion

The pigment composition of 37 phytoplankton strains from banks or environmental collections was examined using an optimized extraction process coupled to an automated HPLC dereplication method. This study revealed the presence of unidentified chlorophyll derivatives and carotenoids in most species studied, in addition to classical pigments already described as taxonomic markers of particular phyla. Interspecific comparisons revealed that some of these unidentified pigments are characteristic of phyla, classes or orders, and may have high interest as stringent taxonomic markers. The Carotenoids Handbook [38] reveals the occurrence of more than 800 carotenoids from natural sources. The same kind of comprehensive and updated analysis bas not really been performed with chlorophyll derivatives but it seems chlorophylls comprise a group of at least 50 tetrapyrrolic pigments [39]. With respect to the acceptance criteria, it was difficult to systematically match our unknown pigments to the literature, but our results can still be considered as a contribution to microalgae chemotaxonomy. With the high biodiversity of phytoplankton species, their diverse molecular adaptations to the photic conditions throughout the evolution of their lineages, and considering that only a few species have been studied, it is obvious that many pigments remain to be identified. This work gives an overview of the biodiversity of carotenoids and chlorophylls in an array of microalgae strains, shows how they can be used as chemotaxonomic markers, which may lead to some of them finding commercial applications.

### Pigment analysis aspects

Some gold HPLC references for phytoplankton pigment analysis can be found in [2,9,40] and [13]. The described methods based on visible spectral analysis and applications are mainly thought to use pigment identifications and concentrations as chemotaxonomic markers, but none of them really exploit the UV part of their spectrum. These tools are powerful for well characterized pigment identifications. Nevertheless, visible spectral analysis is not sufficient when structural identification of pigments is needed. By coupling spectral properties to mass spectrometry analysis it becomes possible to confirm isomers or ester bonds. Studies with mass spectrometry are very powerful for single pigment identification and characterization. However, results depend on ionization rate. This identification technique generally fails when complex pigment mixes are addressed. It is still difficult to get a better overview than spectral analysis. NMR analyses are reliable for pigment characterization especially with the development in recent years of high magnetic field instruments and cryoprobes. Some authors developed a methodology based on HR MAS ^1^H NMR spectroscopy coupled with multivariate analysis aiming to classify microalgae [41]. However, this method is not currently widespread because of the high cost of such equipment. Thrane and co-workers [42] described a Gauss-peak spectra method to simplify algal pigment mixture spectra but this does not solve the community affiliation. It is therefore still useful to develop new tools for a better, faster and more reliable understanding of algal communities in open waters.

Reliable identification and quantification are necessary to unravel phytoplankton contribution to biological and biogeochemical processes affected by climate change. During the process that leads to algal community determination (*i*.*e*., taxonomic distribution), care should be taken of the production of potential artefact pigments during sample storage/extraction/analysis. Some methods can produce unstable, “non-existent natural products”. Methanol, for example, if it comes into contact with a hot point such as the tip of a sonication probe, is likely to produce methyl esters in presence of natural products. However, xanthophylls can also be linked naturally to the membranes as ester forms [38]. Another historical extraction is based on 90% acetone solvent, which is good to inhibit degradation enzymes, but its intrinsic characteristics favour apolar pigment extraction. Although some solvents provide a broad extraction spectrum (*e*.*g*., N,N’-dimethylformamide), it is probably better to choose another extraction strategy to limit the subsequent detection of artefacts [14]. With the growing interest in improving the extraction processes using GRAS solvents (Generally Recognized As Safe) [43] and taking into account the relatively good pigment extraction yield without significant studies reporting formation of degradation products, ethanol 100% based extraction seems to be a good start to deal with natural compounds in phytoplankton chemotaxonomy [13].

Pigment retention time constitutes another important parameter. Times are generally quite reliable within the same batch analysis (±0.05 min). However, variations can occur at the beginning of the elution when the analytic column has become too old and/or the TBAA salts become too oxidized. In this case, it is important to keep a watchful eye on wavelength maxima and the injection of a standard mix between a couple of samples and/or add a non-natural internal standard to each natural sample. Generally, we can consider that retention time requires a loose tolerance in the first 10 min of the elution with the Van Heukelem and co-authors method [13] and identification should rely more on spectra similarities. Finally, a data exploration can be based on different strategies that are more or less sophisticated and computationally managed [44,45]. For example, results can be managed in clusters [46]. Non-taxonomic interpretations of pigment datasets can be realized by pigment indices and by ecological similarity indices [47]. To compare UV-visible spectra, we worked with the algorithm provided by ChemStation (Agilent Technologies) to provide identification scores based on the shape of the spectrum and on relative intensity of the lambda max. However, X-hitting algorithm developed by [48] might be also adapted to discriminate pigments. In our study, we found that many chlorophylls and carotenoids are very similar. The tough task was to define the right criteria in term of spectrum variation making it possible to merge or to discriminate two unknown pigments. The goal was to be flexible enough to not discriminate two short variations related to the detection but selective enough to discriminate two slight structural variations. To help make the decision, the UV range of each spectrum was included in the intercomparison using the ChemStation algorithm. In our experiment, a score ≥999/1000 was considered high enough to merge two pigments. Between 995 and 999, and if the visible results were perfectly aligned, merging of the two pigments was accepted. If the alignment was not very accurate, merging of two pigments was rejected. A score <995 discriminated two pigments straight away. However, sometimes when spectrum intensity was very low (trace compounds), the UV range was not very helpful in the decision-making process due to noise. Only the analyst’s experience could then give an interpretation of the data. This basis allowed us to build consistent Gephi communities, which display the direct viewable links between pigments and related strains.

Then, Canonical Variates Analysis are efficient to discriminate microalgae subgroups [49] (*i*.*e*., the same goal as our study), but do not make it possible to pinpoint chemotaxonomic markers in communities analysis. This strategy is efficient only if molecule selectivity is high (*i*.*e*., sufficiently separated by the chromatographic system). With the latest methods, selectivity of the main pigments is quite good. However, chemodiversity is often hidden in the baseline [50], and some of these molecules are closely structurally related, which means they are not always well separated by a general analytical method. These minor analogues can slightly pollute a Gaussian (*i*.*e*., individual pigment spectra and background components as weighted sums of Gaussian functions), and have to be taken into account in data interpretation. This is sometimes still slightly trickier for minor peaks and traces. Several times, when dealing with a signal with both porphyrin and carotenoid spectral properties, we had to name some minor pigments PCar. These pigments seemed to be pure enough to use the apex as a reference (Retention time and **λ**_max_) in Table 2. These kinds of spectra are not very common but provide a consistent signal [51]. Carotenoids are involved in chlorophyll photoprotection. To carry out this function and the subsequent regulation of energy flow within the photosynthetic apparatus, carotenoids are bound in discrete pigment-protein complexes in the proximity of chlorophylls [52]. Light-harvesting complexes (LHC) display a wide range of architectures in terms of pigment composition, stoichiometry and location in the apparatus. In these complexes, there is a bridging molecule between the chlorophyll and the carotenoid moieties, which is the fifth ligand for Mg in the chlorophyll macrocycle. In dinoflagellates, the LHC is named PCP for Peridinin-chlorophyll *a*-Protein. This complex is water-soluble and the Chl/carotenoid ratio is in favour of carotenoids [53]. Because our method of extraction covers a wide range of polarity and a high extraction rate, it seems likely that we were able to extract this kind of complex in small amounts, even if it is probably not strictly peridinin that is detected in our PCar. Optimized extraction technique and extraction device are referred to [14]. Finally, we can conclude this part of discussion by saying that, in the mid and long term, technological breakthroughs will probably eventually make it possible to identify each trace compound in a complex pigment mix. Currently, the right question to ask is probably whether a peak/spectrum (even incompletely characterized or slightly polluted in the peak tail) useful to distinguish communities or not?

### Chemotaxonomy and markers

Chemical taxonomy is based on investigations of the distribution of compounds or groups of biosynthetically related metabolites in series of related clades, taxa or species. A major challenge is to identify stringent chemotaxonomic markers that unambiguously signal the presence of taxonomic entities in natural samples. Despite the complexity of metabolism, even for photosynthetic unicellular organisms, significant advances have been made in recent decades, notably in genomics. Metabolism is the product of anabolism and catabolism reactions driven by genes and environmental factors. Some biosynthesis genes have been lost or acquired during lineage radiation and this helps us to distinguish clades and phenotypes. However, multiple factors can influence pigment expression. In terms of biogenetic classification, primary metabolites can be distinguished from secondary metabolites. The former are directly involved in physiology (growth, development, and reproduction), and the latter, as far as their functions are understood, are involved in allelopathy mechanisms, aiming to protect algae against environmental stresses, defend them from predators, and/or communicate intra- and interspecifically. The attribution of microalgal pigments to these classes remains unclear and is not very well documented but, according to the definitions, it is obvious that they can play both roles [54]. The analysis of our results raised the question of which kind of metabolites best indicate a plastid type clade, a phylum, a class, or finer taxonomic levels, which is quite difficult to answer based on present knowledge. However, our study has contributed some elements to this discussion.

First, we assume that ephemeral biosynthetic intermediates have little chemotaxonomic value since they cannot be reliable over time. This is probably the case for some pigments such as protochlorophyllide, chlorophyllide derivatives, **β**-cryptoxanthin and **β**-cryptoxanthin epoxide.

Our analysis also showed that major photosynthetic pigments (Chlorophyll *a*, *b*, and *c*) are often not suitable for accurately defining taxonomic groups (*i*.*e*., plastid type clades) as shown on Fig 6. Indeed, chlorophyll *a* is widely distributed because of its major role in both photosystems (II and I). Derivative forms of Chl *a* arise either from its anabolism or its catabolism. The various forms of enzymes responsible can be detected and are quite common in all taxa. However, some classes like Bacillariophyceae are known to synthesize large quantities of chlorophyllases, which are more or less specific [35]. This fact explains why we found a large chlorophyll chemodiversity in our bacillariophyte analyses. Moreover, epimerases are highly substrate-selective as well as promiscuous. Some traces of minor pigments likely result from cellular enzymes and oxidations, and the difficulty is to discern which of them are sufficiently specific to become reliable markers. Moreover, microalgae that are adapted for growth in extreme biotopes or with a particular metabolism might express specific enzymes modifying common signalling pathways and/or modifying common pigments. In this case, the pigments produced could be good candidates to serve as chemotaxonomic markers and possibly also as molecules of interest for biotechnologies. For example, this could explain why we found a stringent pigment combination for *Galdieria sulphuraria* (*Gs*) strain (Car50, Car57, P4, P22).

On the other hand, some degradation pigments (*e*.*g*., red pheophorbides, chlorophyll catabolites, primary fluorescent chlorophyll catabolites and non-fluorescent chlorophyll catabolites) cannot be used as markers. It is also the case with pheophytin *a* for which the sample storage and extraction process would likely increase the amount of this pigment. In our case—with collection at the end of exponential growth, no storage before analysis and an optimized soft extraction—conditions were not met to produce significant amounts of degradation products. Since pheophytin *a* is probably the most widely distributed degradation product in natural communities, it cannot be considered as a specific marker for microalgae chemotaxonomy but does however have an interest for monitoring phytoplankton populations senescence. In this case, when studying degradation pigments it is more appropriate to work at 405 nm rather than 436 nm.

Fluctuations in pigment quantities are usually a rapid response to environmental factors because most of pigments are involved in basic processes such as photosynthesis and photoprotection. Cells need to be reactive to survive in a very competitive and rough environment. The fast pigment conversion levels results without any changes in gene expression, although long-term acclimation causes changes in the transcript level of the genes, which encode proteins in biosynthetic pathways [55]. In Table 4, we propose a list of hypothetical markers resulting from pigment metabolism that are presumably genetically driven. We hypothesize that strong markers can be found in these metabolites [18,56]. Some of these molecules may not be detected by visible spectroscopy. Hence, acquisition of UV spectra has potential advantages, even though it has not been exploited in phytoplankton chemotaxonomy. This methodology has been applied to plant chemotaxonomy [57] but most of the time, UV detection is coupled with other detectors like mass spectrometry or ELSD [58] to cluster different taxa. The field needs innovative breakthroughs to identify next generation of markers. Hyphenated analytical tools like HPLC-UV DAD-MS^n^, which have a dereplication strategy, could become a future means of expanding microalgal chemotaxonomy beyond strict pigment detection and taking into account more secondary metabolites.

**Table 4:** List of genetically-driven molecules involved in pigment biosynthesis pathways.

**CHLOROPHYLL AND PORPHYRIN PATHWAYS**
**Biosynthetic intermediates**
Porphobilinogen
Uroporphyrinogen
Protoporphyrinogen IX
Mg-Protoporphyrin IX 13-monomethyl ester
Billiverdin
Bilirubin
Mesobilirubinogen
Protochlorophyllide
Divinyl-protochlorophyllide
Divinyl-chlorophyllide a
**Ending products**
Uroporphyrin I
Coproporphyrin I
D-Urobilin
I-Urobilin
L-Stercobilin
Bacteriochlorophyll a
Bacteriochlorophyll b
Bacteriochlorophyll c
Bacteriochlorophyll d
Zn-Bacteriochlorophyll a
**CAROTENOID PATHWAYS**
**Biosynthetic intermediates**
4,4'-Diapo-neurosporene
Hydroxy-spirilloxanthin
3,4-Dihydro-anhydrorhodovibrin
7,9,9'-tricis-Neurosporene
Phytofluene
Neurosporene
Zeinoxanthin
α- and **β**-Cryptoxanthin
**ζ**- and **γ**-Carotene
**β**-Zeacarotene
Spheroidene
[3R,2'S]-Myxol 2'-**α**-L-fucoside
5-Deoxy-strigol
3-Hydroxy-echineone
Caloxanthin
Xanthoxin
3'-Hydroxy-abscisate
**Ending products**
Staphyloxanthin
4,4'-Diapo-lycopenedial
Rhodopinal glucoside
2,2'-Diketo-spirilloxanthin
Tetrahydro-spirilloxanthin
[2S,2'S]-Oscillol 2,2'-di[**α**-L-fucoside]
R.g.-Keto III
Okenone
Lutein
3,4-Dihydro-spheroidene
7,8-Dihydro-**β**-Carotene
Hydroxy-chlorobactene glucoside ester
[3R,2'S]-Myxol 2'-[2,4-di-O-methyl-**α**-L-fucoside
[3S,2'S]-4-Ketomyxol 2'-**α**-L-fucoside
Strigol
Astaxanthin diester
Isoreneriatene
Thermo-biszeaxanthin
Zeaxanthin diglucoside
Nostoxanthin
Capsanthin
Capsorubin
Dihydroxy-phaseic acid

This non-exhaustive list was built by mining KEGG pathway maps, map 00906 for carotenoids and map 00860 for porphyrins and chlorophylls [accessible at www.genome.jp/kegg updated version on 18^th^ April 2016].

Biosynthetic intermediates could be detected in microalgal analyses but, according to the discussion above, probably constitute weak markers for chemotaxonomy. Ending products could be also detected. These molecules are more likely able to be accumulated and remain stable in cells. They could constitute strong markers for photosynthetic microorganism chemotaxonomy. This hypothesis is strengthened by the fact that lutein is accumulated in some of our samples belonging to CHLORO-1 and EUGLENO-1 types. Similarly, our experience with biosynthetic intermediates showed that violaxanthin was not always detected in our *Cb* analyses. Moreover, **β**-cryptoxanthin was not present in our *Cp* analyses but detected in the same strain by Baudelet et al. [23].

Molecules listed for carotenoid biosynthesis belongs to terpenoid backbone biosynthesis, unusual spirilloxanthin pathway, normal spirilloxanthin pathway, okenone pathway, lutein biosynthesis, spheroiden pathway, myxol biosynthesis, and astaxanthin biosynthesis. Some pathways belong to cyanobacteria, alpha- and gamma- proteobacteria displaying photosynthetic pigments. Detection of markers belonging to these pathways is valuable since biologists are faced with a wide variety of microorganisms in natural samples, not only to eukaryotic microalgae. Because genes from metabolism were acquired or lost during evolution, microalgae clades do not express the same gene patterns and nor the same suites of pigments. Genomic data mining for microalga species is currently quite limited so it is not that simple to conclude whether a gene has been lost or if it is insufficiently expressed to be detected. For strains with which we have the most experience, intermittent presence of some pigments means that the related genes are conserved but where a pigment is absent, and background information is sparse, conclusions should be drawn with caution.

Among all the factors influencing pigment-related gene expression metabolism, UV has been particularly studied [59–62]. Irradiance and spectral distribution of light are also of course directly involved in photosynthesis [63–71]. The related aspects of photoperiod [72] and circadian rhythm [73] have an impact on microalgal pigment composition. Low temperature culture or an anoxic environment can lead to partial conversion of methyl-pyropheophorbide in meso-a pyropheophorbide [74]. Nutrients also affect the relative concentrations of pigments [75–78]. Mixing regimes of water masses, at a culture or meso scale level, are also influential factors [79,80]. According to species, pigmentation can change depending on the growth phase [81]. For example, xanthophyll to chlorophyll ratio increases throughout the microalgal growth phase and declines once in stationary phase in the genera *Amphidinium* and *Euglena* [82]. Thus, it is widely accepted that the environmental conditions of microorganisms can affect their metabolism. A methodology has thus been recommended that aims to vary systematically different culture parameters (composition of media, aeration, temperature, pH, addition of enzyme inhibitors etc.) to increase the number of available secondary metabolites from a single strain [83]. This methodology, called OSMAC (One Strain—Many Compounds), optimizes the investigation of chemodiversity produced by a single strain of a microorganism. Changes in culture conditions and inducing stresses would modulate different biosynthetic pathways, thereby increasing the metabolic diversity of a strain and the possibility of obtaining original compounds for biotechnology applications and chemotaxonomy purposes [84]. Fluctuations in pigment diversity (including minor ones) likely arise from both types of metabolism, but minor compounds probably belong more to secondary metabolites or participate in pigment turnover. To improve our general understanding of this phenomenon, and although the experimental logistics would be heavy, holistic studies could be performed mimicking ecosystem complexity in terms of biological communities (biocenosis) and biotopes. The present study has not explored these aspects so far but provides a way in which such studies could be pursued.

Micro-organism co-cultivation makes it possible to study different kinds of ecological behaviour. Symbiosis and predation can both affect phenotypes [85,86]. Among the analysed strains, *Chlorarachnion reptans* (Cr) illustrates that some species can predate other photosynthetic species. It is well known than some dinoflagellates can practice kleptoplastidy (*i*.*e*., retain plastids from different strains) [87]. Regarding the *Cr* strain, we were not able to determine whether this was really the case as we did not identify the major markers of its prey, the diatom *Nitzschia curvilineata*. It seems that the kleptochloroplast turnover is variable, but it might be relatively fast after feeding on a prey [88], which could be the case for *Cr*. This kind of behaviour should be taken into account for data interpretation as should some molecules that remain silent as long as there is no co-cultivation (or co-presence) with other microorganisms (*i*.*e*., allelopathy effects). Our results are mostly based on monospecific cultures, which may constitute a bias compared to multispecies samples collected in open waters. A validation of our ending markers (yellow boxes in Fig 6) by oceanographers would be welcome as this would modulate their scope, as Tamm and co-workers did with lake samples [89].

Regarding chemotaxonomy and lineage evolution, this study raises two main issues. The first question is about whether the Chlorarachniophyceae should belong to the Chromista as is presently accepted. Our findings support the hypothesis that the Chlorarachniophyceae could belong to CHLORO-1 plastid type and so, to the green lineage, rather than the brown lineage (Chromista) in terms of pigments. Indeed, *Bn* and *Cr* both contain all the markers belonging to the green lineage (*i*.*e*., Chl *b*, Viola, 9-*cis* Neo, Car12, and Lut). However, Chlorarachniophyceae plastids are surrounded by four membranes like the Chromista, which is a sign of a secondary endosymbiosis, while green alga plastids are bound by only two membranes [90]. A probable explanation that is now quite well accepted is that the original secondary host phagosome was not photosynthetic and thus different from the original Chromista secondary host [91]. Then, Euglenozoans belonging to Protozoa display Diato and Diadino pigments which are part of the Chromista photoprotection apparatus. They also harbour the violaxanthin photoprotection system (Viola-Anthera-Zea), which belongs to the green lineage. So, both known photoprotection systems seem to be viable. This could indicate a recent tertiary endosymbiosis in terms of evolution, a hypothesis that is strengthened by the fact that Euglenids display a three-membrane topology [92] like dinoflagellates, which are well known for their tertiary endosymbiosis (one membrane has been lost during the evolution). The taxonomy of this algae group is still contentious. Further corroborating studies concerning ultrastructural characters (membrane plastids, microtubules, starch, pyrenoid, etc.), biochemistry (isoprenoid biosynthetic enzymes, photorespiratory enzymes), and molecular phylogeny (single or combined genes, intron presence, genome arrangement, etc.) still need to be performed to elucidate the evolution of these groups.

As mentioned by Wright and Jeffrey [93], one of the issues in phytoplankton chemotaxonomy is that many taxa share the same pigment patterns and often cannot be identified based on pigment combination alone. Ten years after their article, this situation is more or less the same despite the exploration of minor pigment diversity. Indeed, among the 32 algal groups defined by [93], our study examined 14 and did not distinguish a stringent pigment combination identifying strains *Pp*, *Cp*, *Cb*, *Saa*, *Hp*, *Ds*, *Ig*, *Oa2*, *Cmu*, *Cmi*, *Ctl*, *Cg*, *Tp* or *At2*. However, we found a couple of markers for the other strains that we studied. Another relevant challenge is to identify stringent markers applicable to a particular plastid type clade, which this is much more complex. Two strategies can be considered. The first one, which is the weaker of the two, is to look for known stringent strain markers in natural multi-species samples to gain a qualitative overview of the clades present. However, without a stringent pigment combination for each species likely to develop in monitored environments it is risky to extend this conclusion further. For this strategy, Fig 6 provides 55 new (or unidentified) strain-specific pigments. The second strategy is to look for pigments that are common to a single clade. Our study highlighted Car36, which is common to the Glauco-1 and Rhodo-1 plastid types, as well as Car1, Car7, Car16, Car17, and Car20 for all *Alexandrium* strains analysed (Dino-1 plastid type). We did not identify other pigments specific to a taxon, even within the genera where we analysed several representatives (*Chaetoceros*, *Odontella*, *Dunaliella* and *Scenedesmus*). This latter strategy is interesting, but analyses of pure strains in monitored environments are still essential to strengthen the conclusions. Quantitative measurements are obviously important for understanding the dynamics of phytoplankton communities. However, traces or minor pigments are difficult to quantify accurately, especially when these pigments remain unidentified (*i*.*e*., no available standard). Moreover, the ratios with Chl *-a* or *-b* would have little chemotaxonomic value since chlorophyll cellular quota can vary widely among strains, the environmental conditions and the extraction technique [47]. This is why we did not include any quantification is the present study. It seems there is still a scientific gap to be overcome before we can fully use minor pigments in phytoplankton community monitoring. At the present time, the best way to move ahead could be to work from relative quantifications (peaks area) with a standard operating procedure for all the steps leading to pigment analysis.

To conclude this part, all these data illustrate part of the cellular plasticity of microalgae and could be used as references for qualitative data. To give a broad idea of pigment quantities and characteristics (traces, minor and major pigments), we have provided chromatograms and spectra in the Supplemental data (S2 and S3 Figs). These should be interpreted with care. Only a part of the microalgal biodiversity is known and described. It is important not to over-interpret natural sample analyses since many unknown algal types still remain to be identified from the global ocean [1]. Absence of some markers therefore cannot be used to conclude about the absence particular taxa from natural samples. Absence of evidence is not the same as evidence of absence. The untapped chemodiversity of microalgae remains a good opportunity to find chemotaxonomic markers. However, microalgal cellular plasticity, linked notably to the activation of silent or weakly expressed microbial gene clusters, still represents a challenge to the identification of strong markers. Hence this is a truly complex field.

### Perspectives for future research

Major pigments of microalgae are well known and easily identified by UV-DAD detector in standard chromatographic conditions. However, when biologists or oceanographers conduct field studies on phytoplankton samples, they are confronted with a large chemodiversity including minor or variable pigments, pigments present in inconsistent quantities, and trace pigments. Using all this data in a rational way to understand the dynamics of phytoplankton populations remains a challenge. In this study, we proposed a guide to several specific chemotaxonomic markers that may be used to explore microalgae pigment diversity/complexity, and pinpointed important factors that can modulate data interpretations. Ninety-eight pigments were new or undescribed carotenoid or porphyrin derivatives with original spectral properties, but whose planar structure, relative structure and absolute configuration remain to be established. Nevertheless, our outcomes highlight that even non-fully characterized pigments could be used in oceanography and limnology. This microalgae community analysis can also be useful as a prioritization tool for aquaculture, drug discovery and other applications involving pigment chemodiversity. In our study, it was decided not to vary culture light irradiances in order to avoid increasing complexity in our analysis. Our results should be considered as pigment fingerprints (*i*.*e*., metabolic snapshot in defined conditions) that we could call the endopigmentome, since the exometabolome was not studied in this project. Using the same methodology, future work could focus on the same strains, varying environmental factors and experimental design. A meta-analysis of our study, combined with the following related studies and then an comparison with field data could be extremely relevant for discerning which markers are the most reliable.

## Supporting information

S1 Fig **(A) Calculation of carotenoid band ratio.** UV-visible spectra showing wavelength I; II and III of a carotenoid. Band ratio corresponds to the % ratio Δ III/ Δ II. **(B) Calculation of porphyrin band ratio.** UV-visible spectra showing the location of I the Soret (blue maximum) and II the red bands of Chlorophyll -*a*.
(PDF)

S2 Fig Chromatograms at 436 nm of all carotenoid and chlorophyll pigments detected in the 37 analysed strains. (PDF)

S3 Fig Spectra of unidentified pigments. (PDF)

S1 Table Pigments and derivatives used as chemotaxonomic markers of phytoplankton strains, species, and classes. (PDF)

S1 Dataset Gephi community (.gephi). (ZIP)
