# Supplementary material for: Community analysis of pigment patterns from 37 microalgae strains reveals new carotenoids and porphyrins characteristic of distinct strains and taxonomic groups
Source: PLoS One. 2017 Feb 23;12(2):e0171872. doi: 10.1371/journal.pone.0171872 (PMC5322898; doi:10.1371/journal.pone.0171872)
Supplement: S1 Fig — (A) Calculation of carotenoid band ratio. UV-visible spectra showing wavelength I; II and III of a carotenoid. Band ratio corresponds to the % ratio Δ III/ Δ II. (B) Calculation of porphyrin band ratio. UV-visible spectra showing the location of I the Soret (blue maximum) and II the red bands of Chlorophyll -a. (PDF) [file pone.0171872.s001.pdf]

Supporting Information

S1.A Fig. Calculation of carotenoid band ratio.

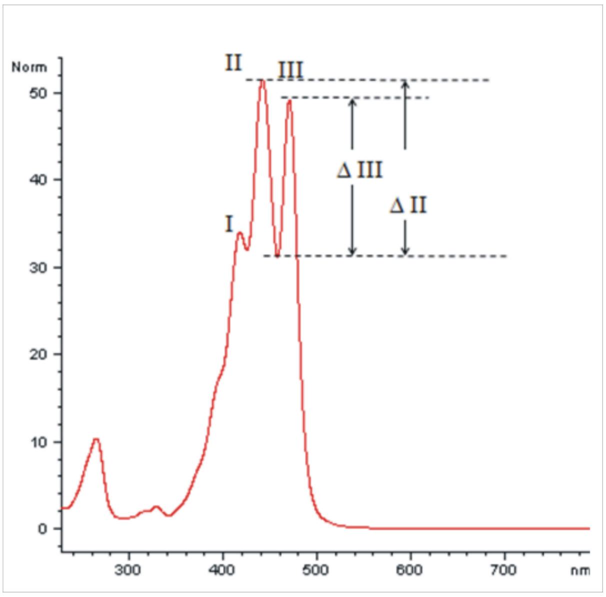

UV-visible spectra showing wavelength I; II and III of a carotenoid. Band ratio corresponds to the % ratio Δ III/ Δ II.

S1.B Fig. Calculation of porphyrin band ratio.

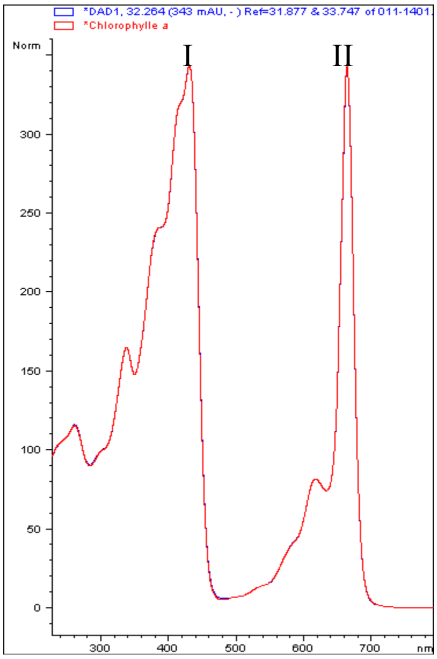

UV-visible spectra showing the location of I the Soret (blue maximum) and II the red bands of Chlorophyll -*a*.
