# Supplementary material for: Community analysis of pigment patterns from 37 microalgae strains reveals new carotenoids and porphyrins characteristic of distinct strains and taxonomic groups
Source: PLoS One. 2017 Feb 23;12(2):e0171872. doi: 10.1371/journal.pone.0171872 (PMC5322898; doi:10.1371/journal.pone.0171872)
Supplement: S2 Fig — (PDF) [file pone.0171872.s002.pdf]

Supporting Information

S3 Figs. Chromatograms at 436 nm of all carotenoid and chlorophyll pigments detected in the 37 analysed strains. Ancillary pigments are annotated according to Table 2.

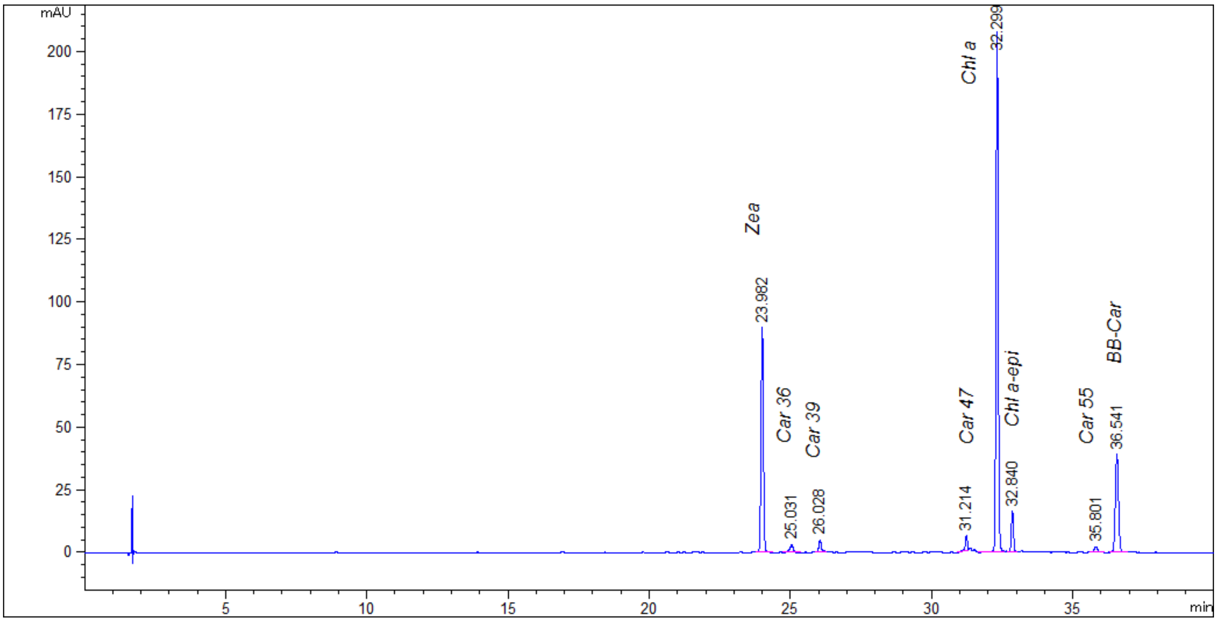

Porphyridium purpureum SAG 1380-d

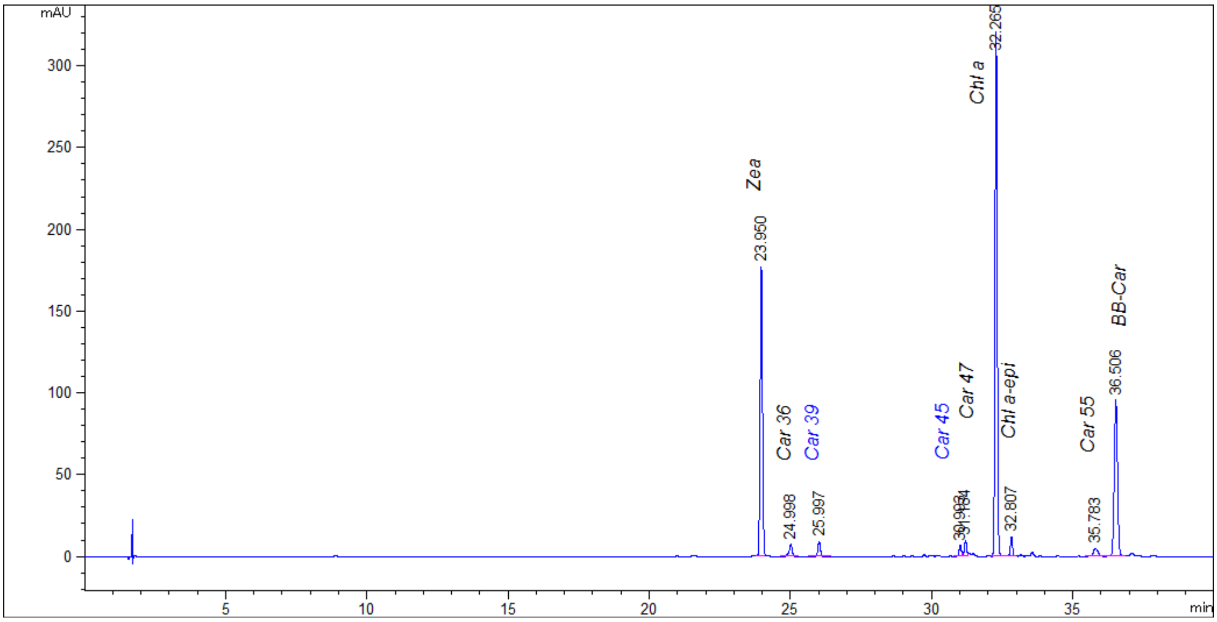

Rhodella violacea SAG 115.79

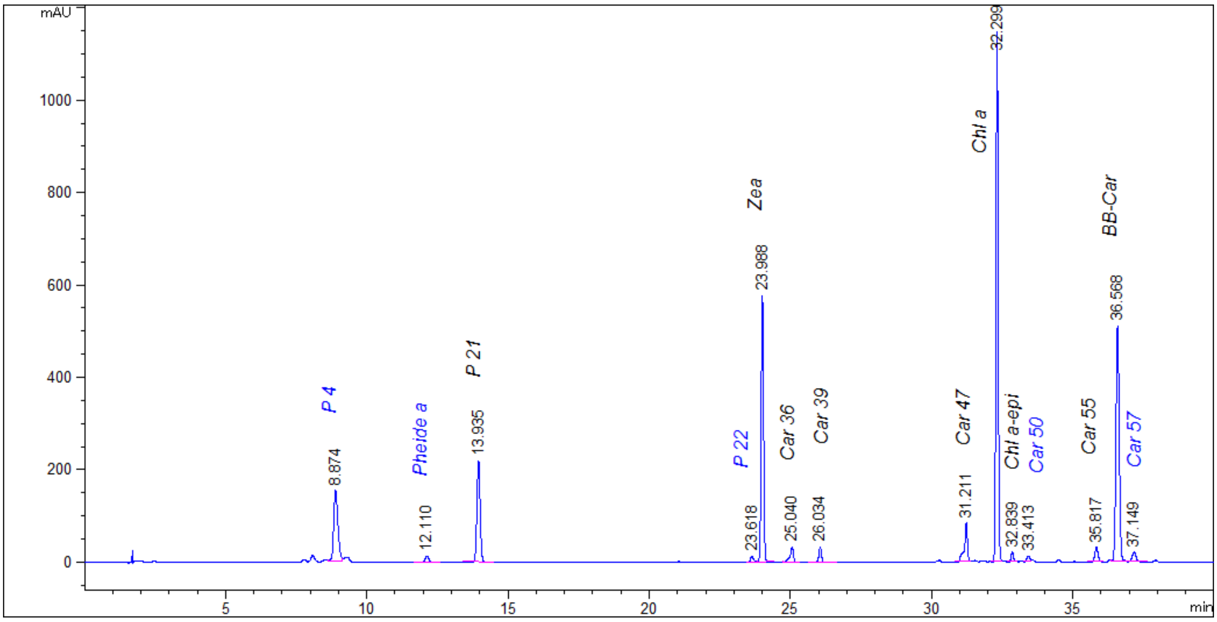

Galdieria sulphuraria

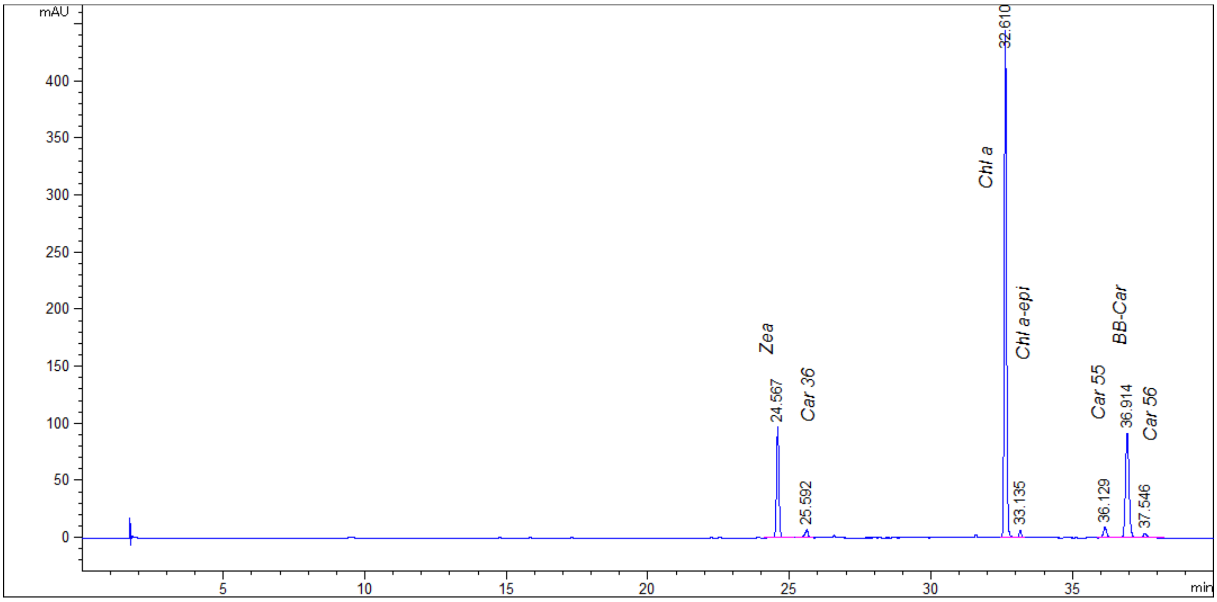

Cyanophora paradoxa **SAG 29.80**

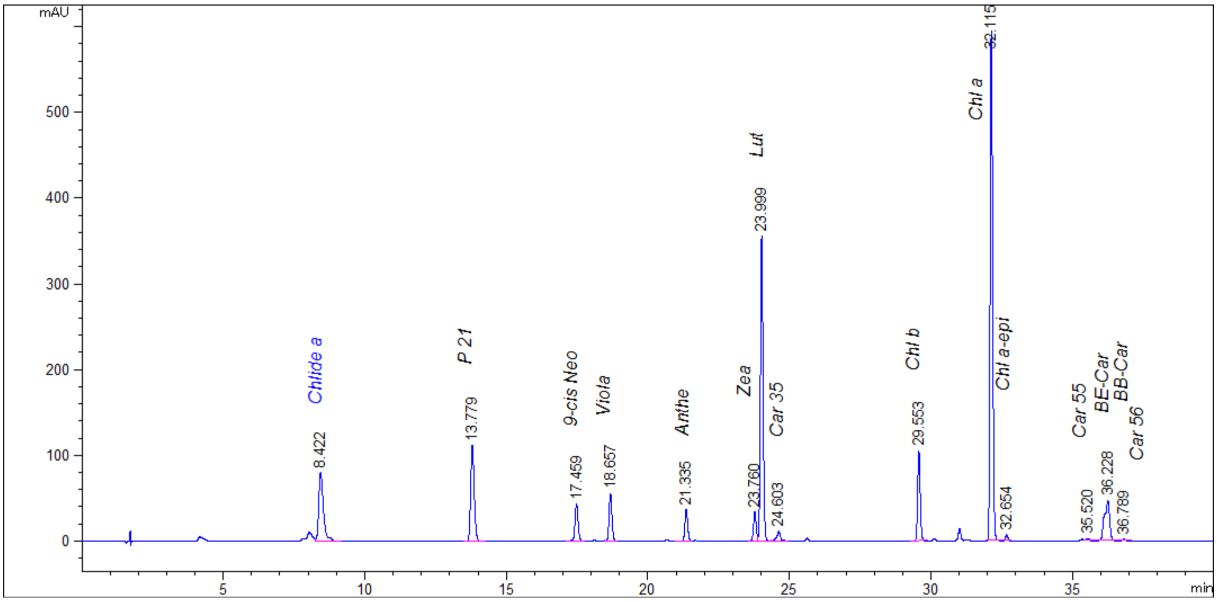

***Closterium baillyanum* SAG 50.89**

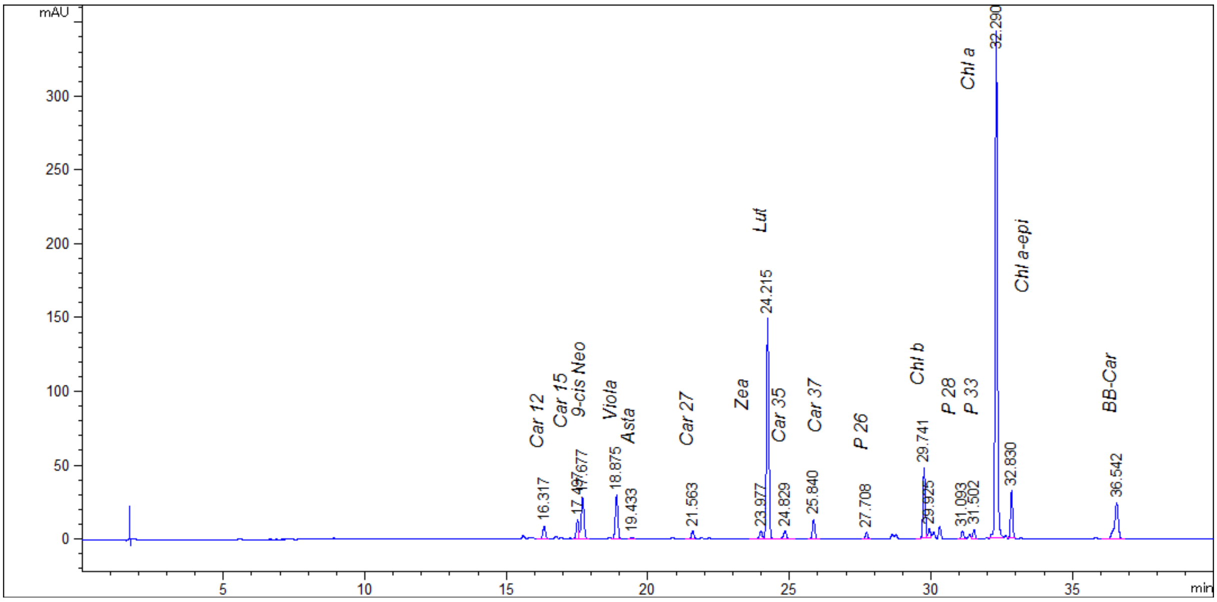

***Scenedesmus acutus f. alternans* UTEX 72**

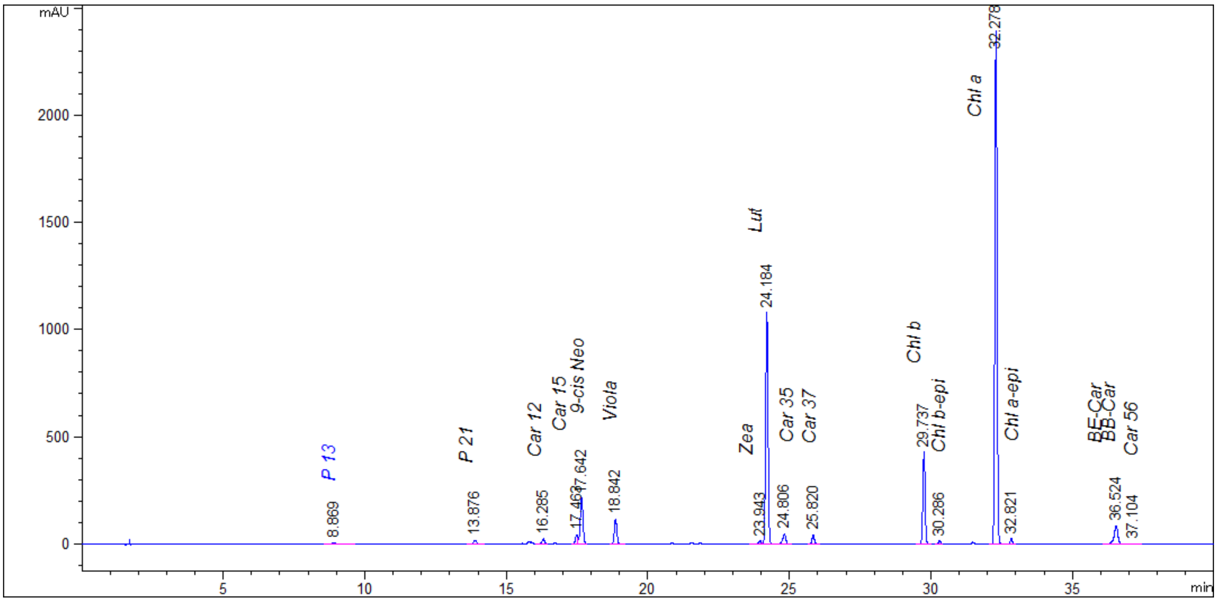

Scenedesmus obliquus **UTEX 1450**

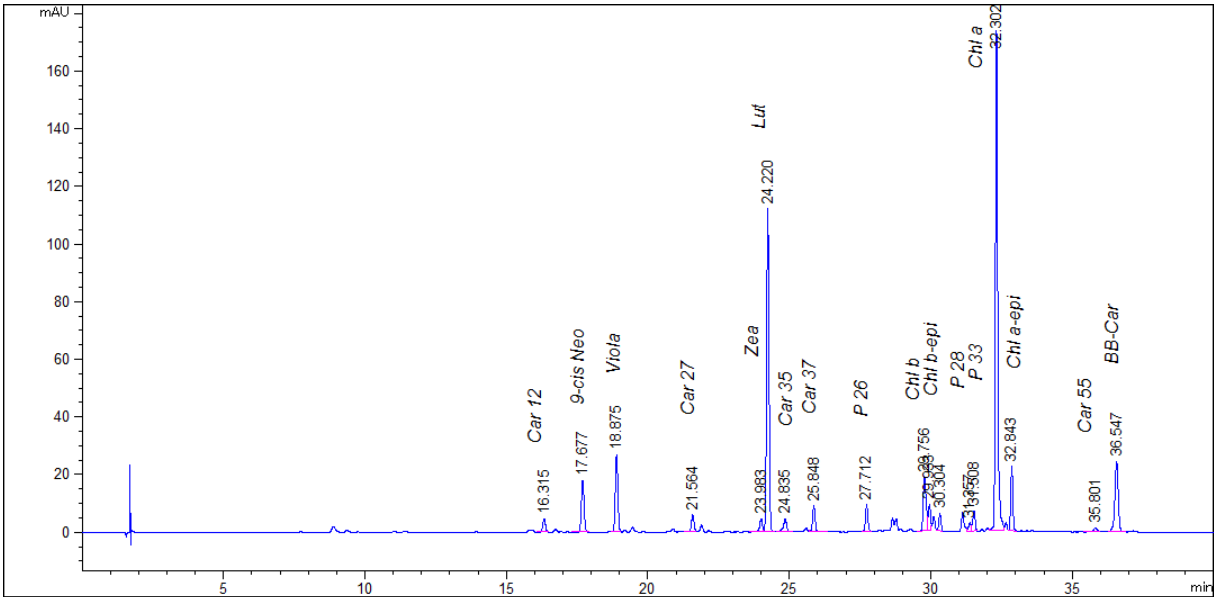

Haematococcus pluvialis **CCAP 34/7**

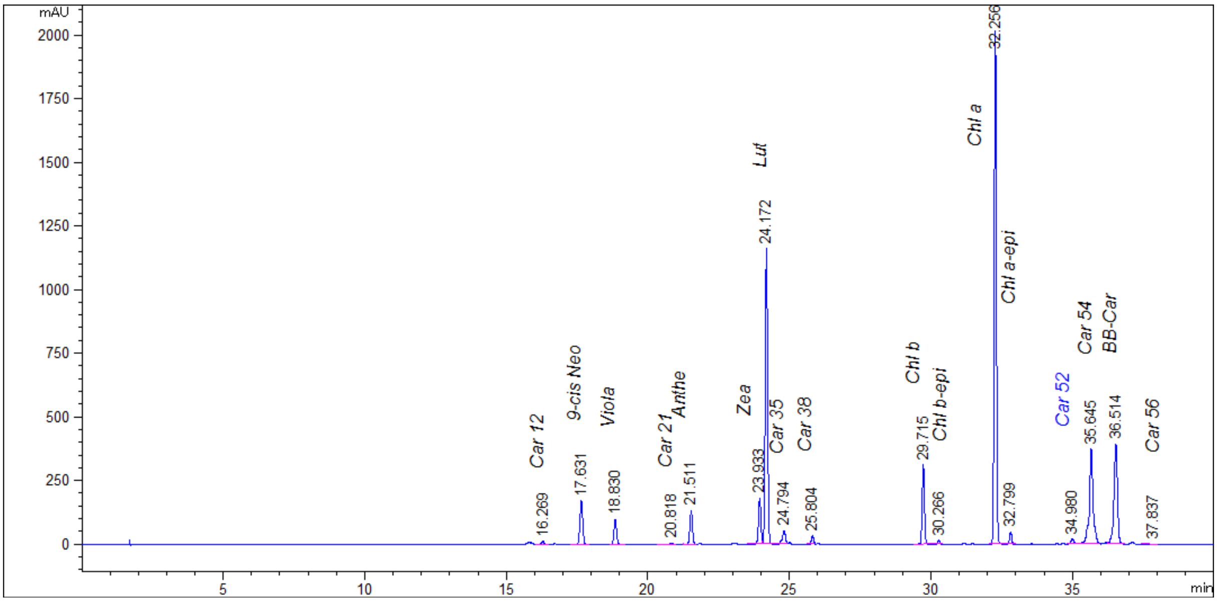

***Dunaliella* sp. CCAP 19/19**

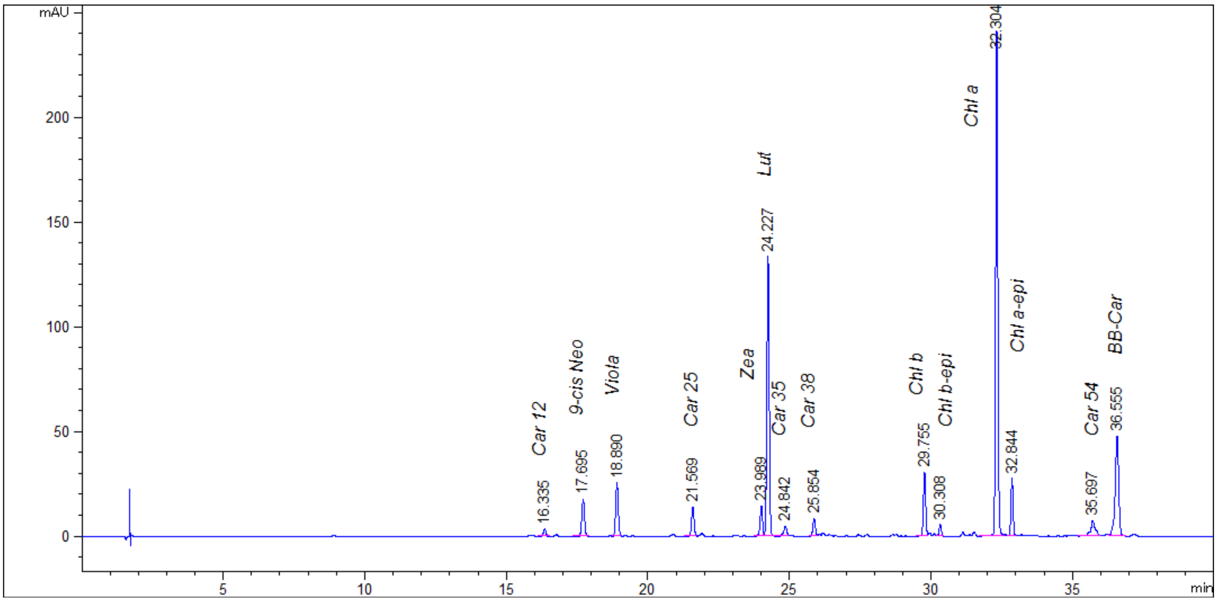

***Dunaliella salina* SAG 19-3**

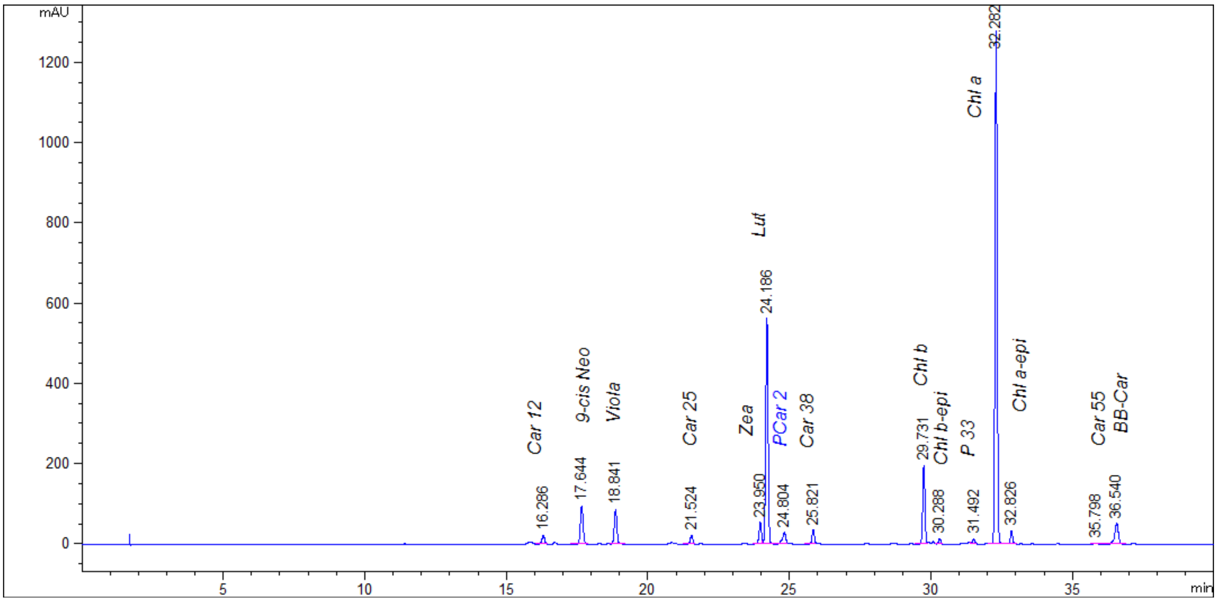

***Chlorella autotrophica* CCMP 243**

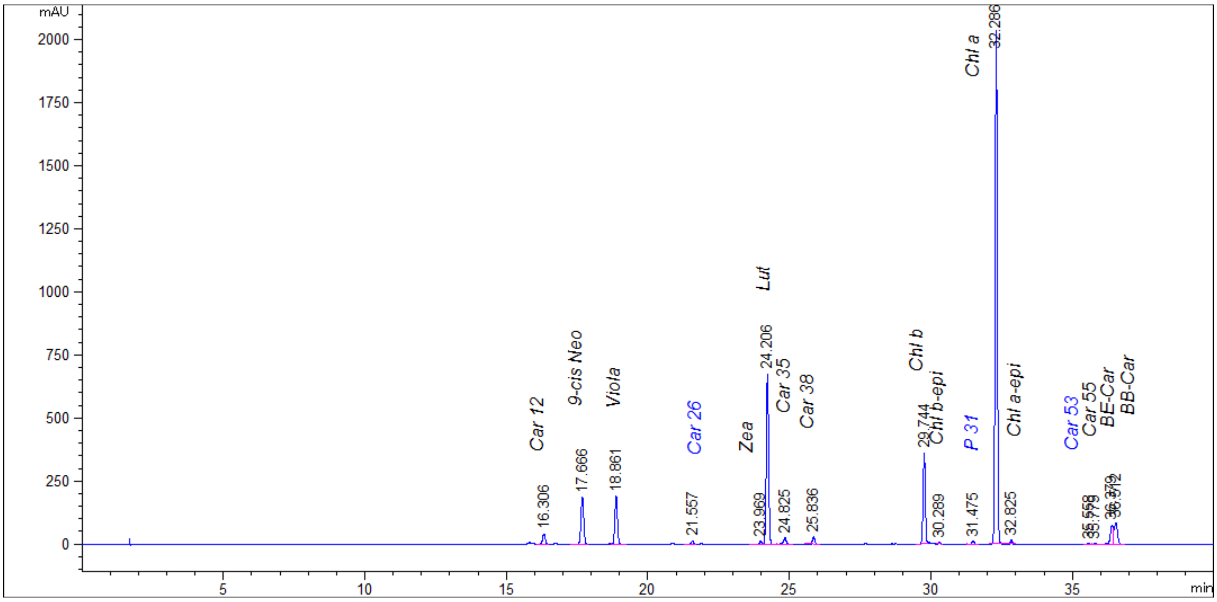

***Chlorella vulgaris* SAG 2.80**

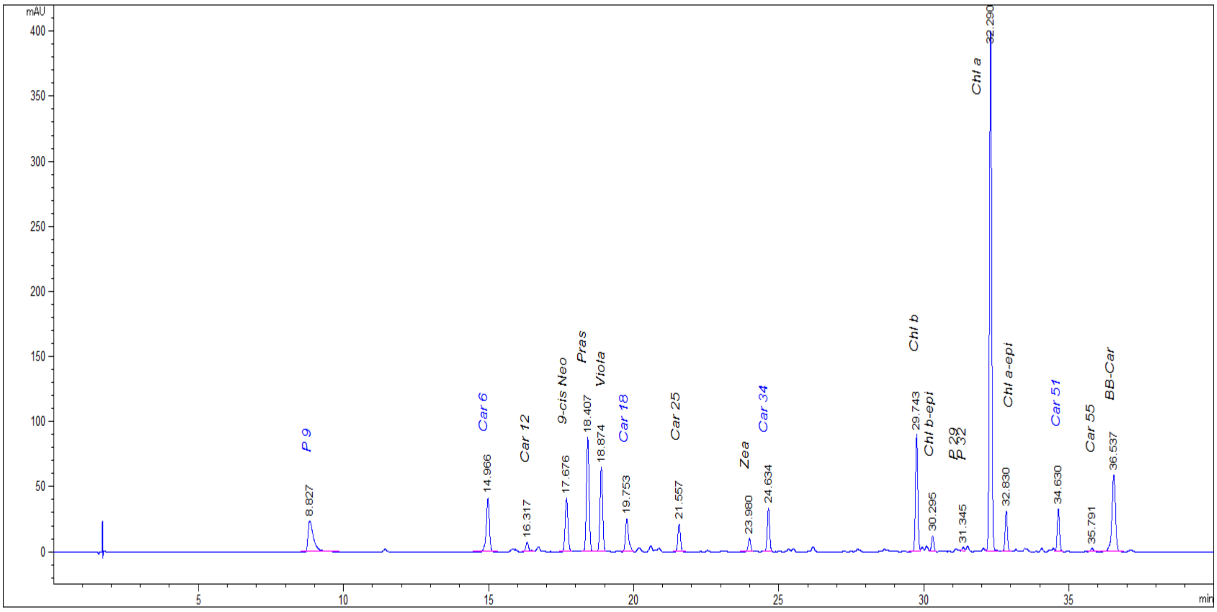

***Ostreococcus tauri* H95**

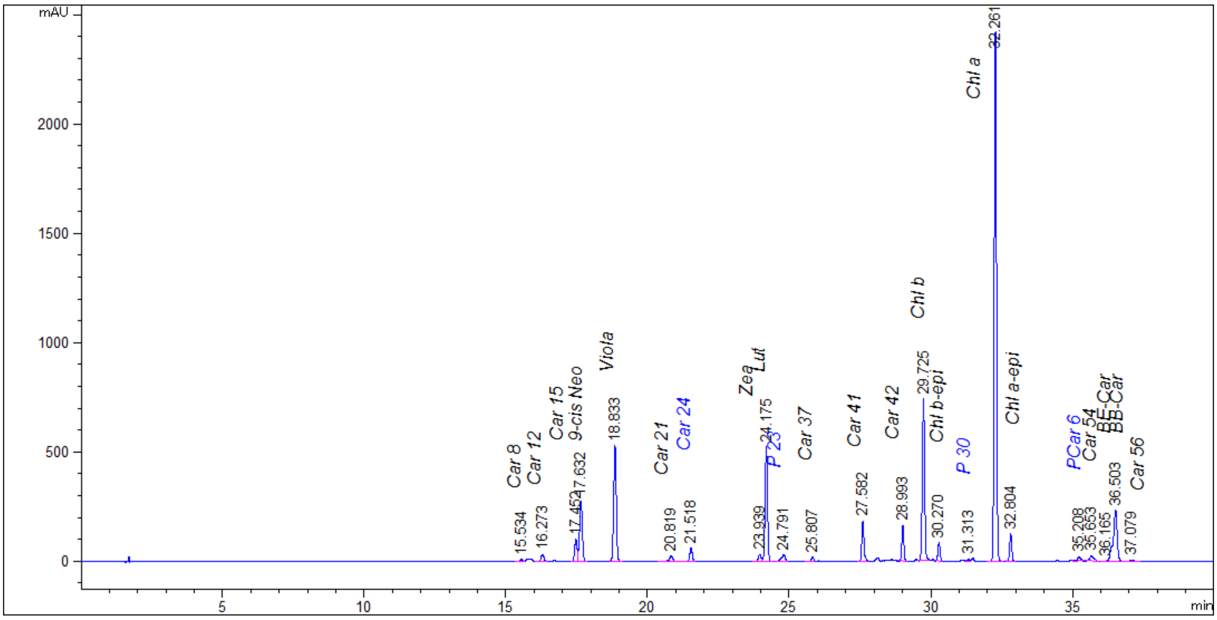

***Tetraselmis suesica* CCMP 904**

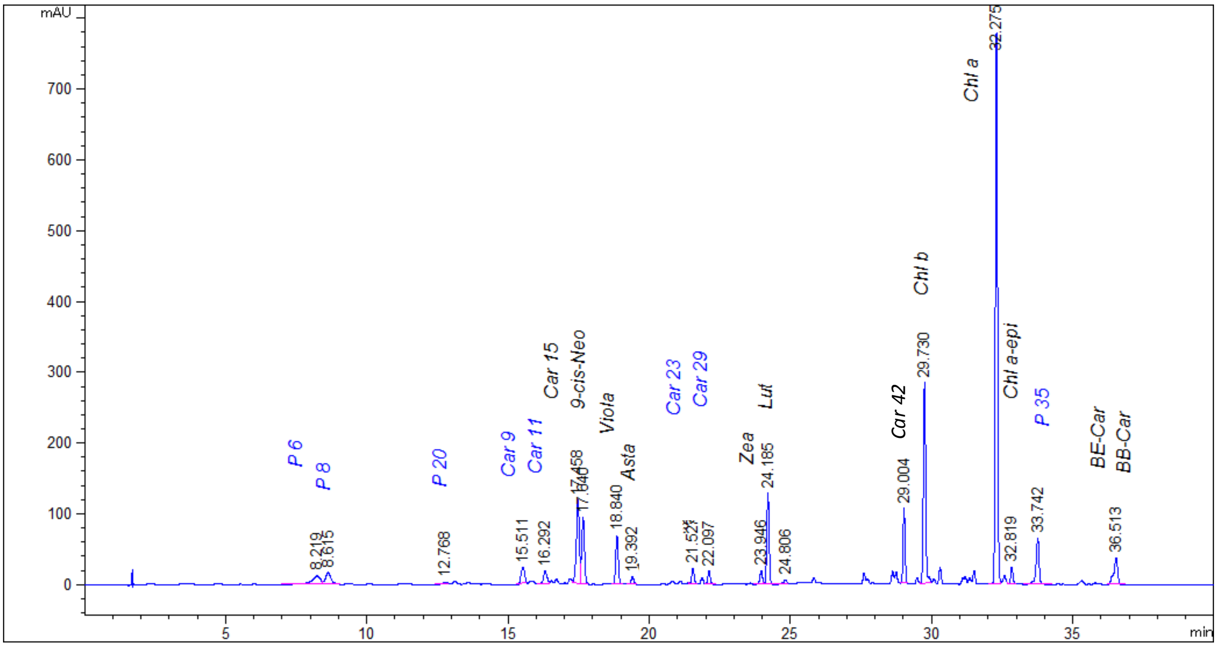

Chlorarachnion reptans SAG 26.97 (fed with Nitzschia curvilineata.)

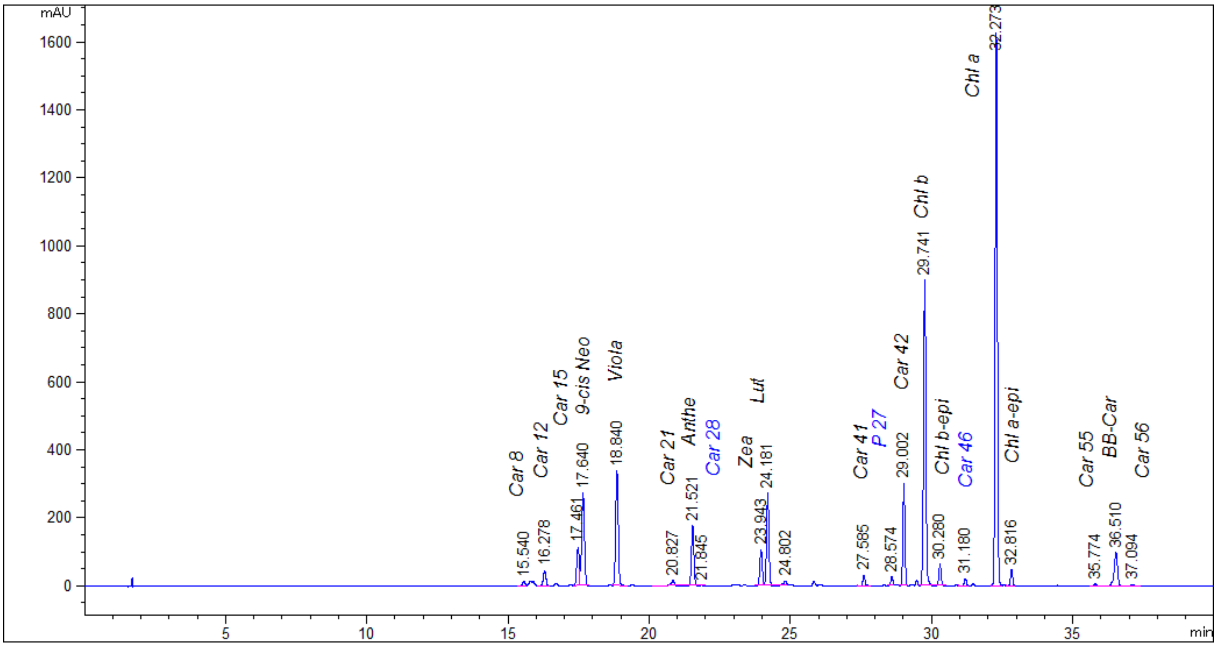

Bigelowiella natans CCMP 621

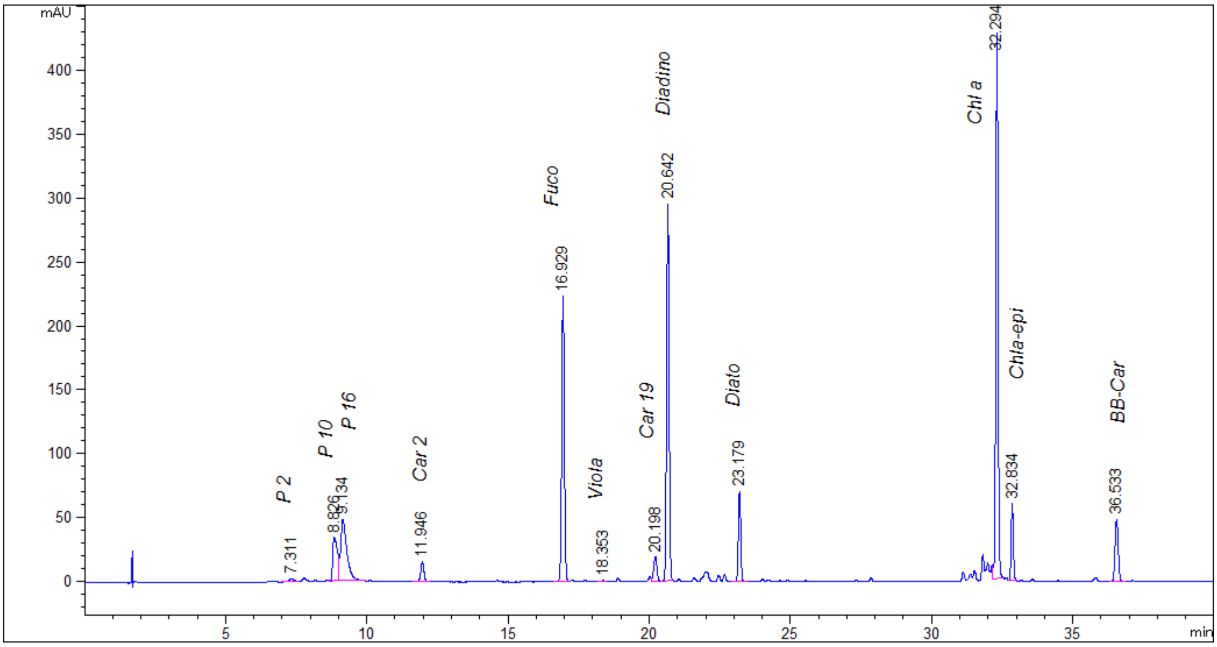

Isochrysis galbana SAG 13.92

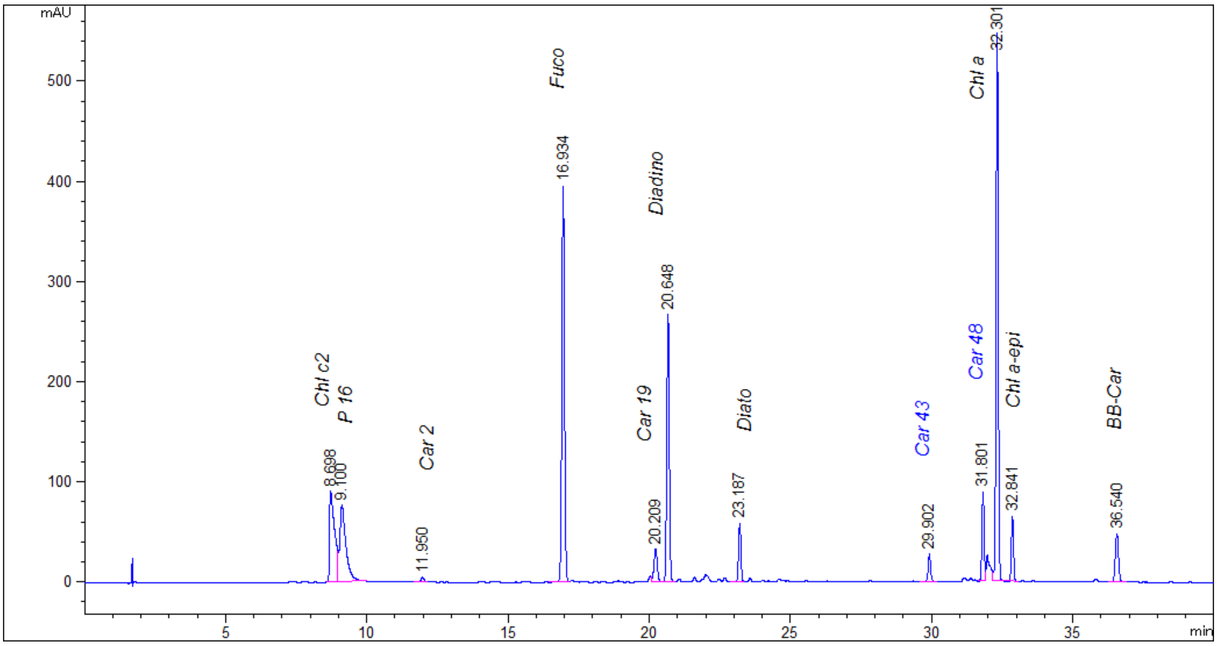

***Tisochrysis lutea* CCAP927/114**

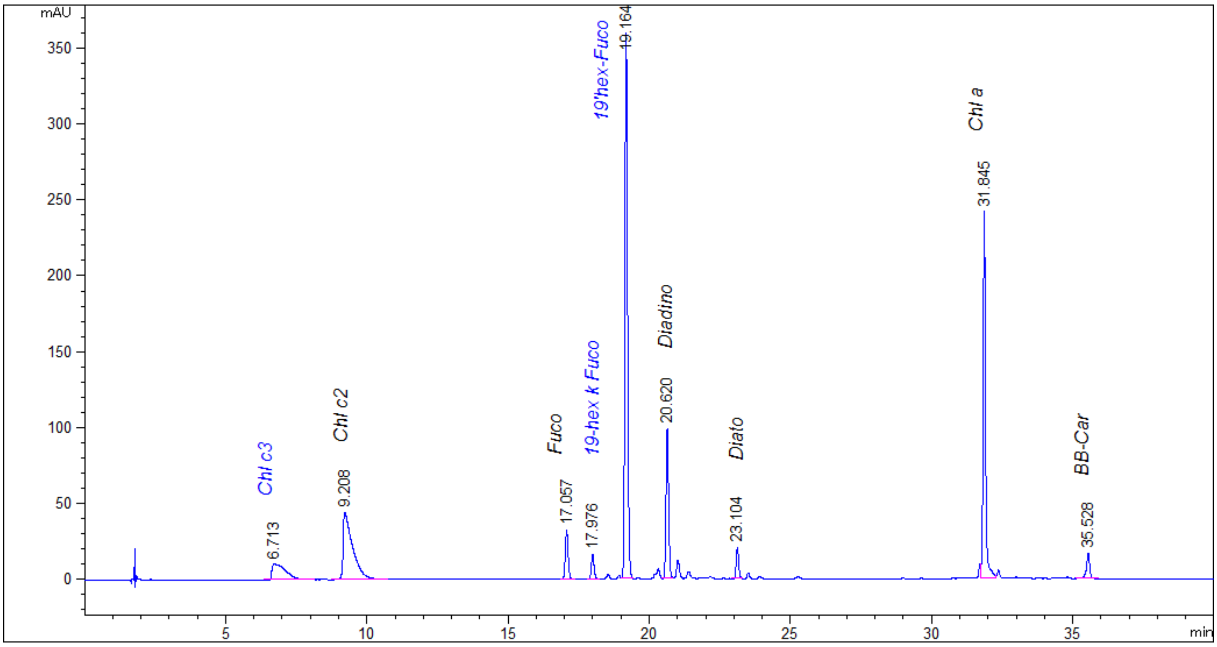

Emiliania huxleyi **SAG 33.90**

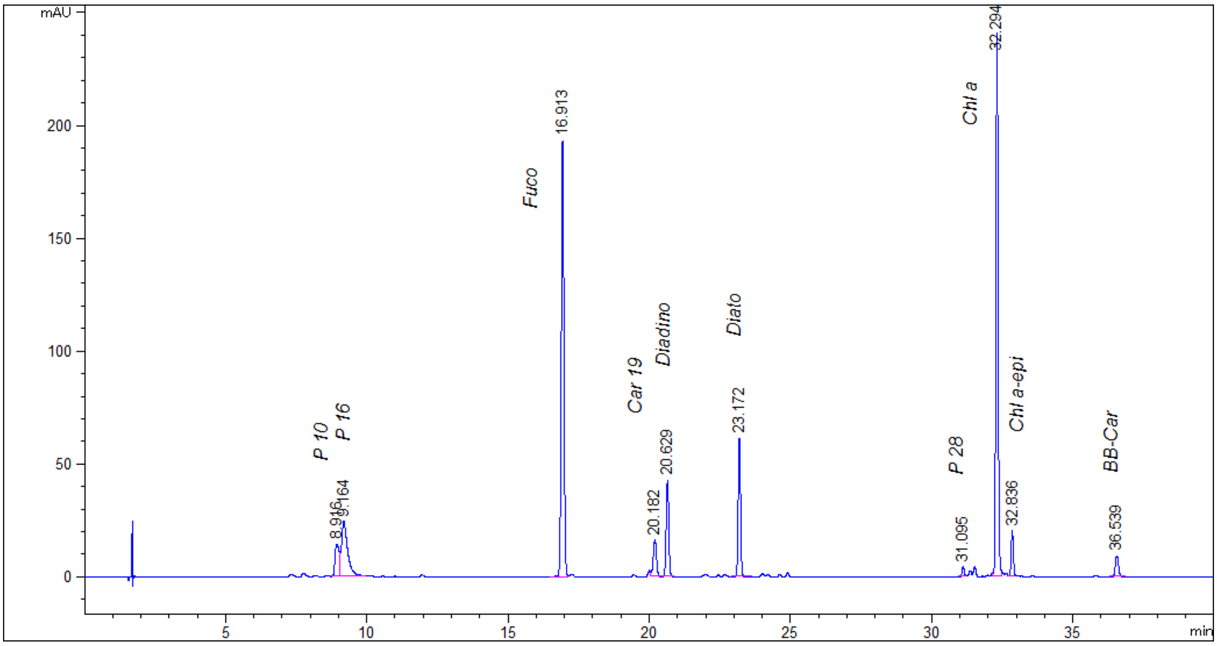

Thalassiosira pseudonana SAG 1224-11a

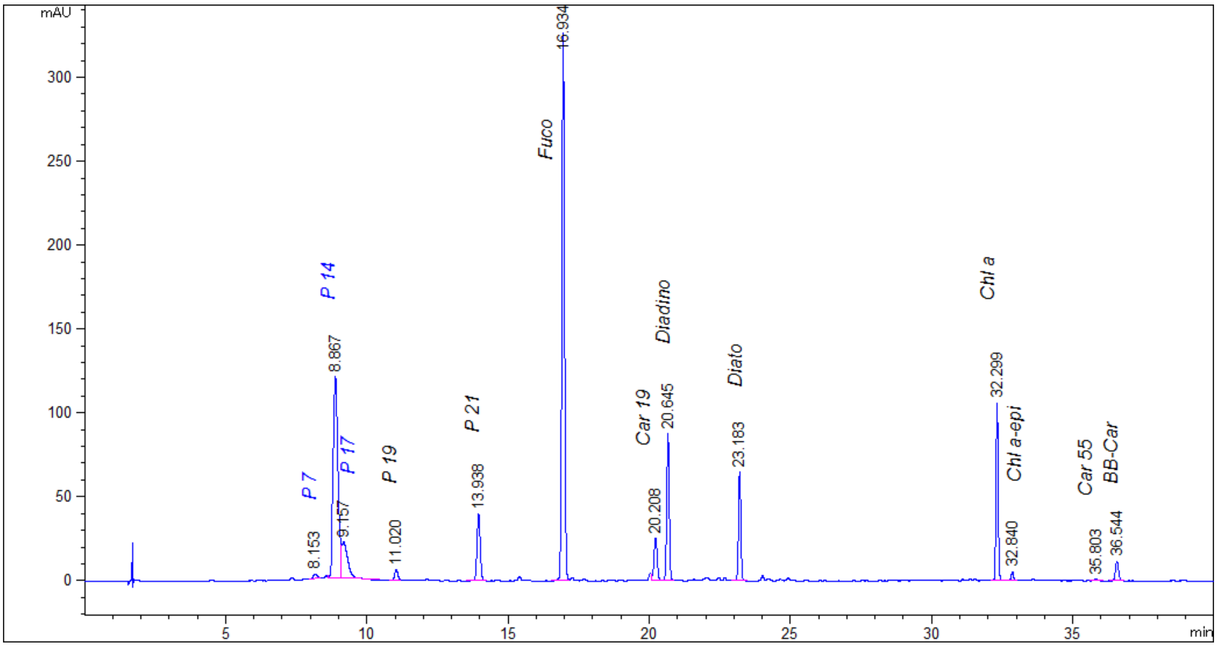

Skeletonema grethae **CCAP 1077/4**

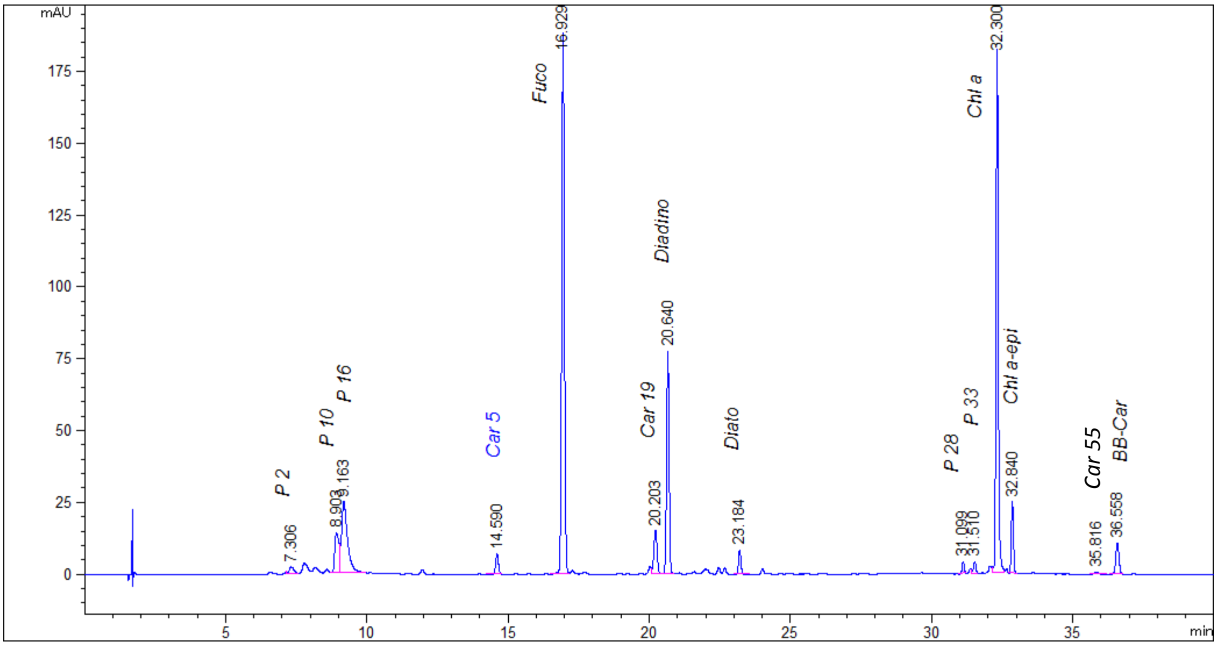

Odontella aurita **123**

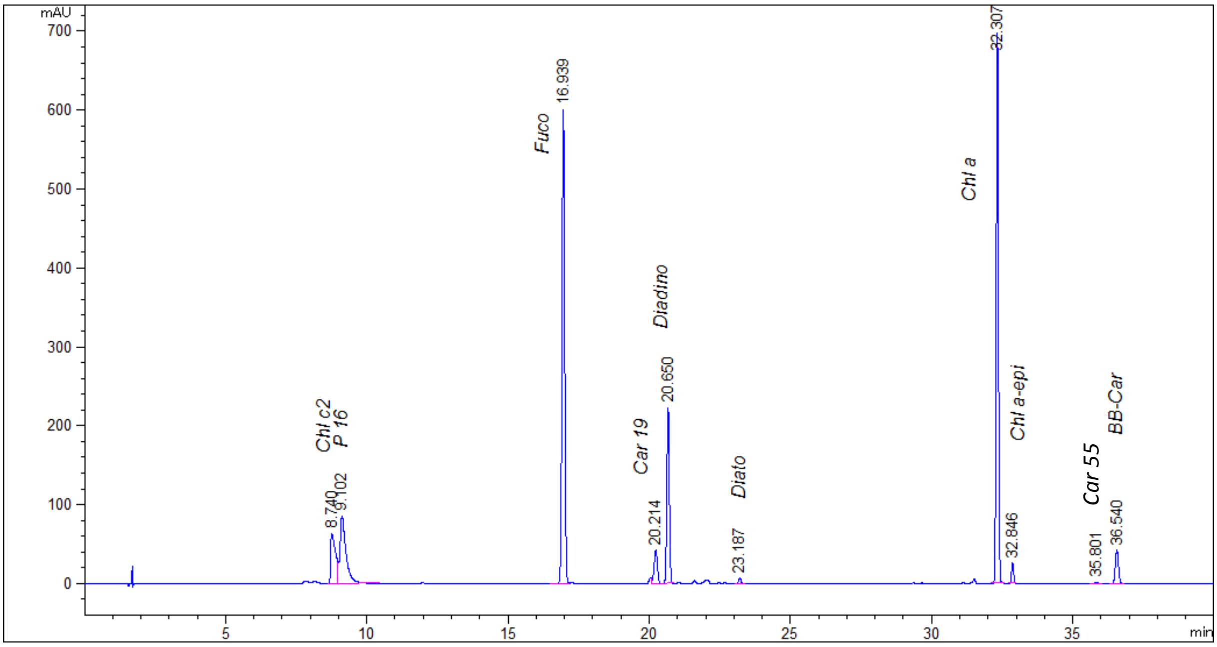

Odontella aurita **122**

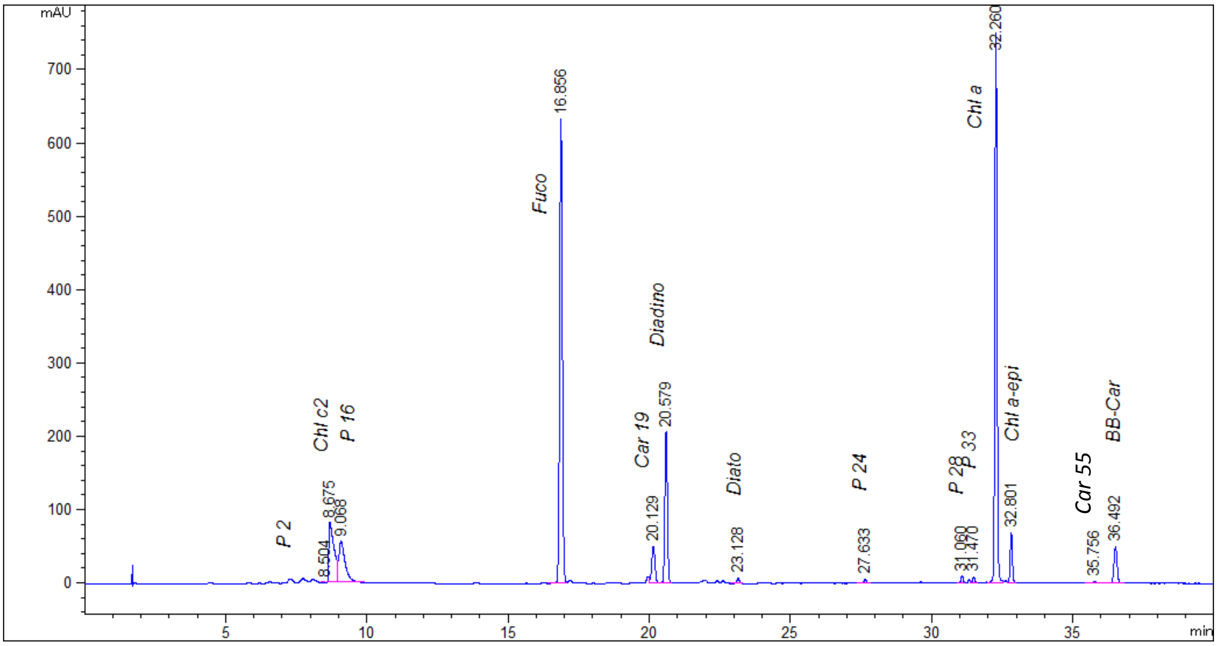

Chaetoceros *mulleri*

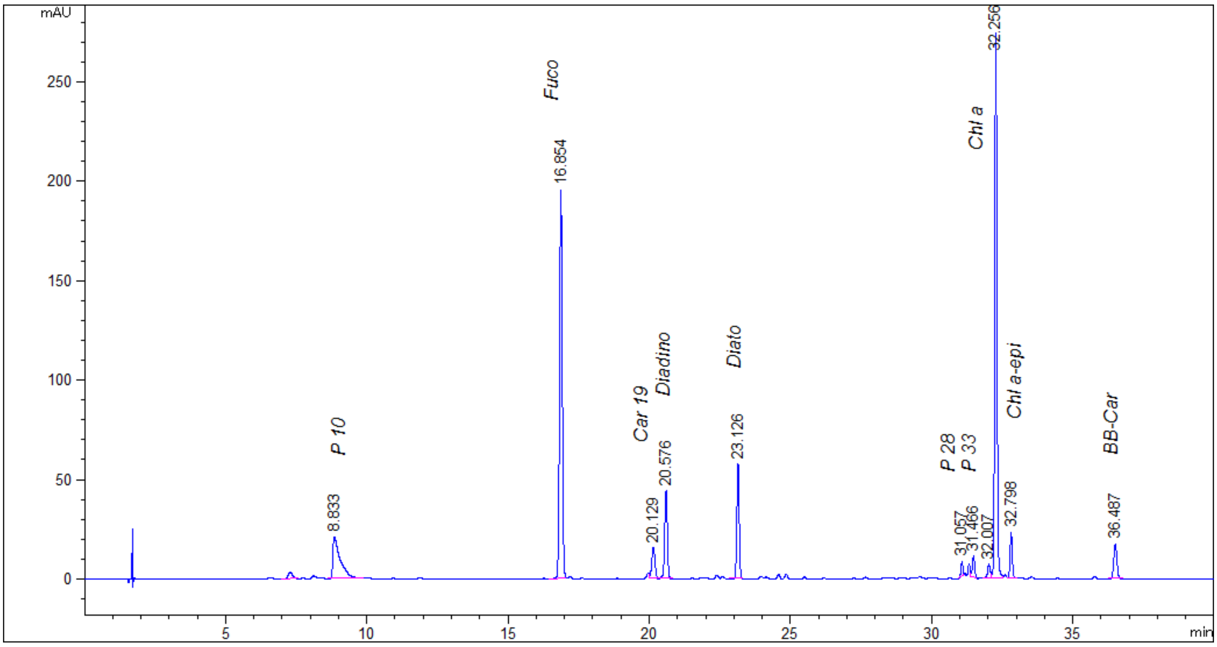

Chaetoceros minus

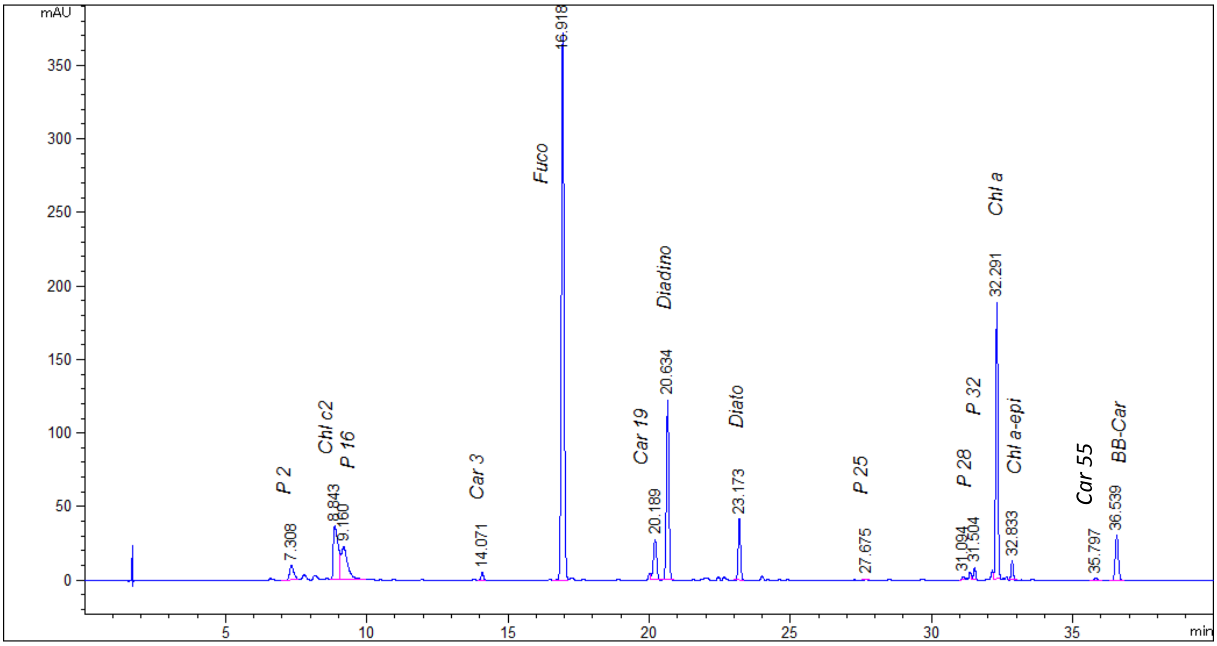

***Chaetoceros* sp. *tenuissimus* like**

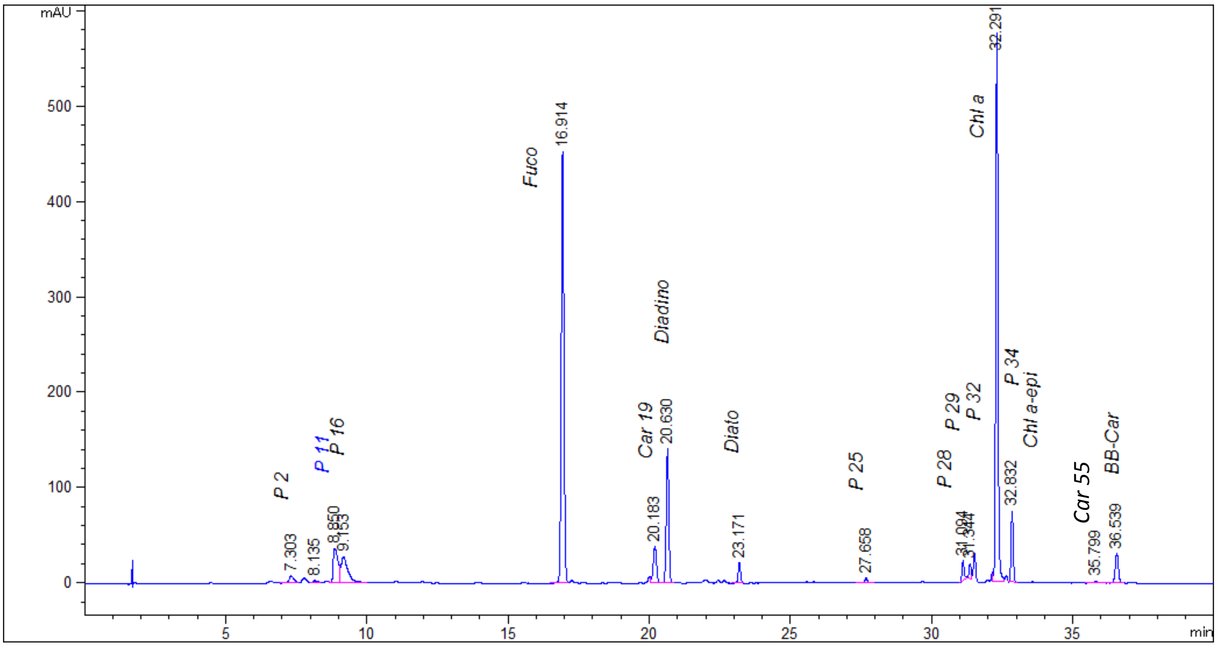

Chaetoceros calcitrans f. pumillum **CCAP 1010/11**

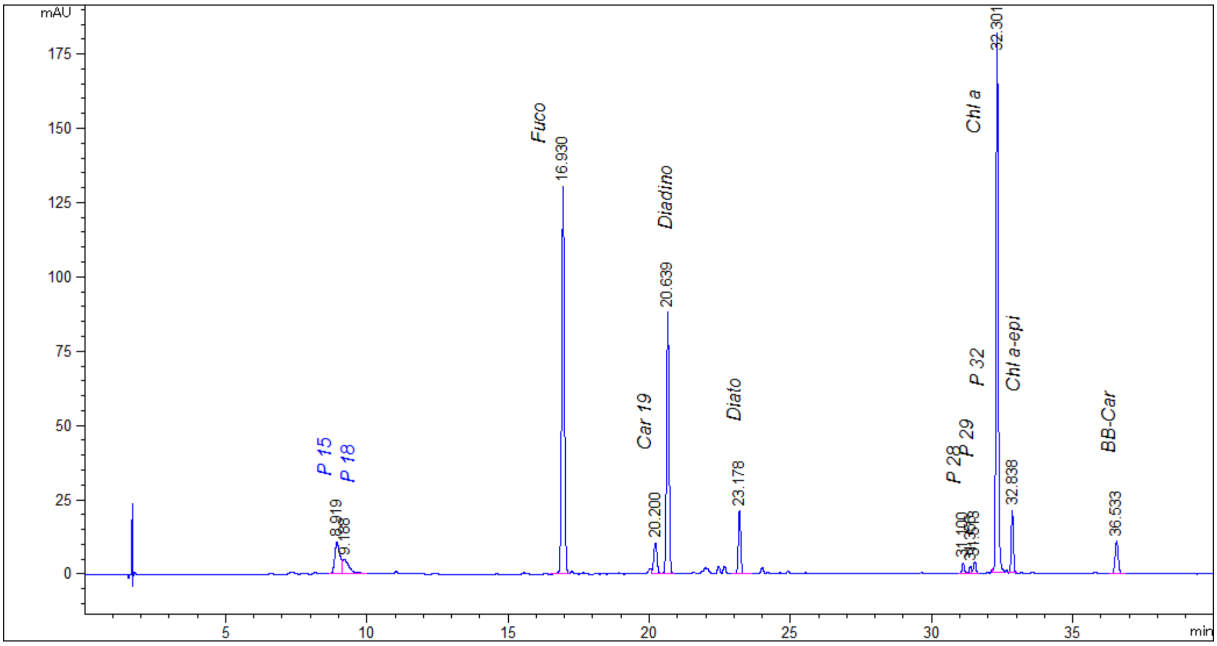

Chaetoceros calcitrans **CCMP 1315**

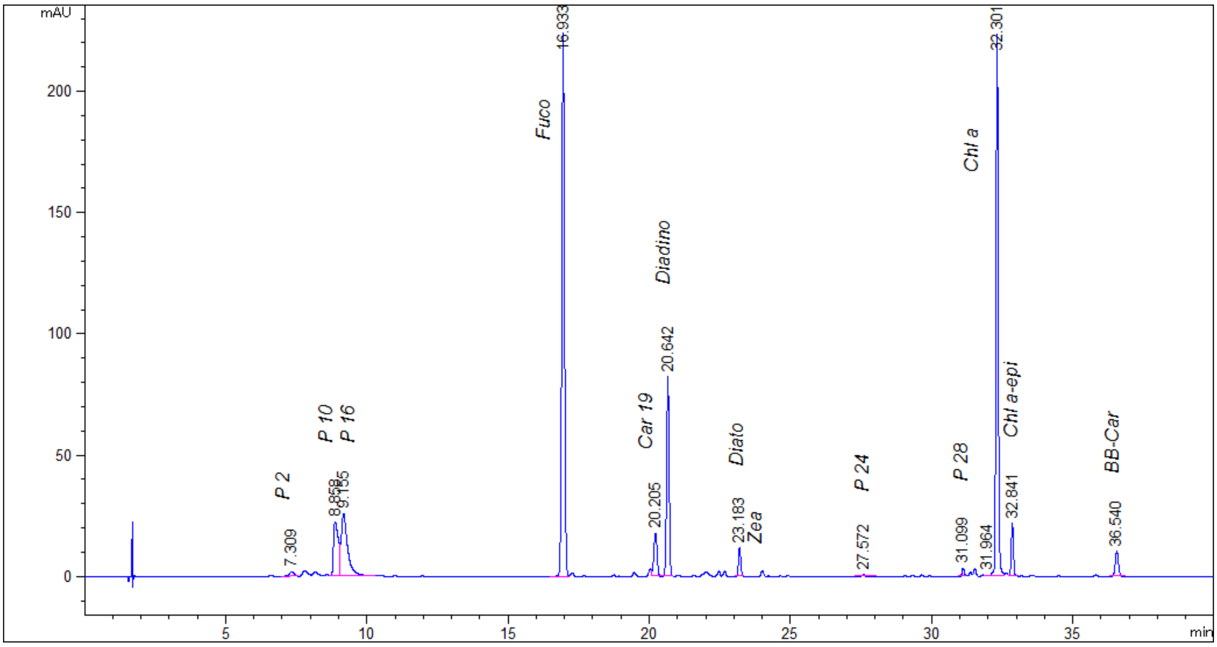

Chaetoceros gracilis **UTEX LB 2658**

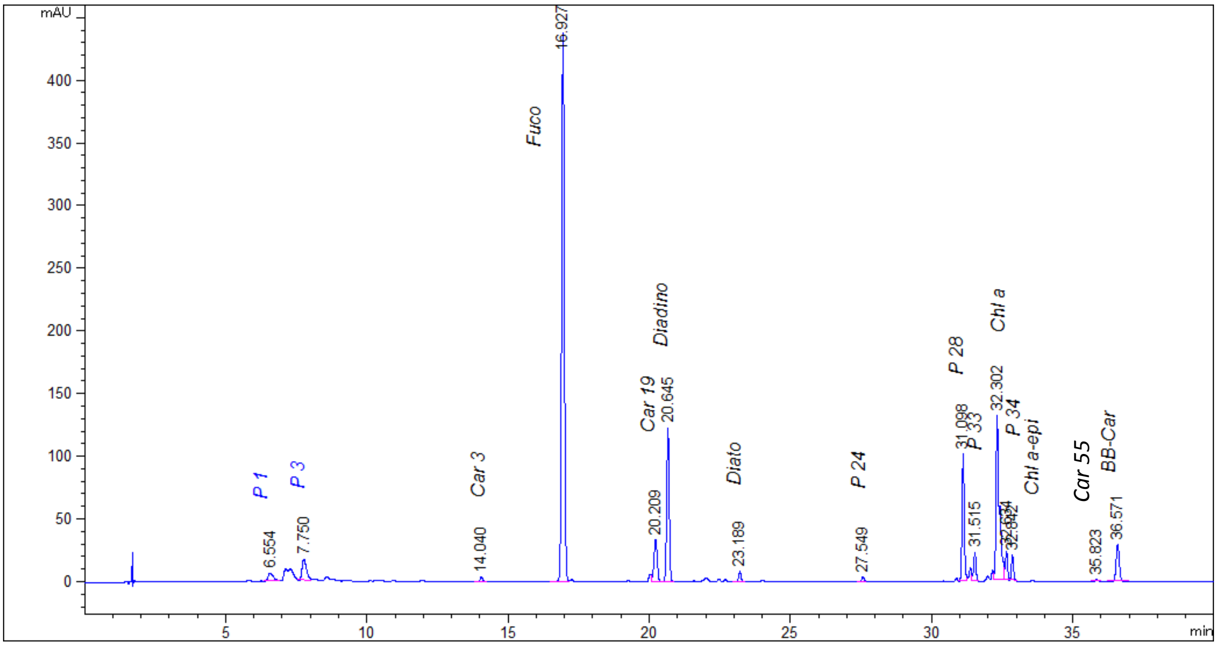

Nitzschia **sp. CCMP 2526**

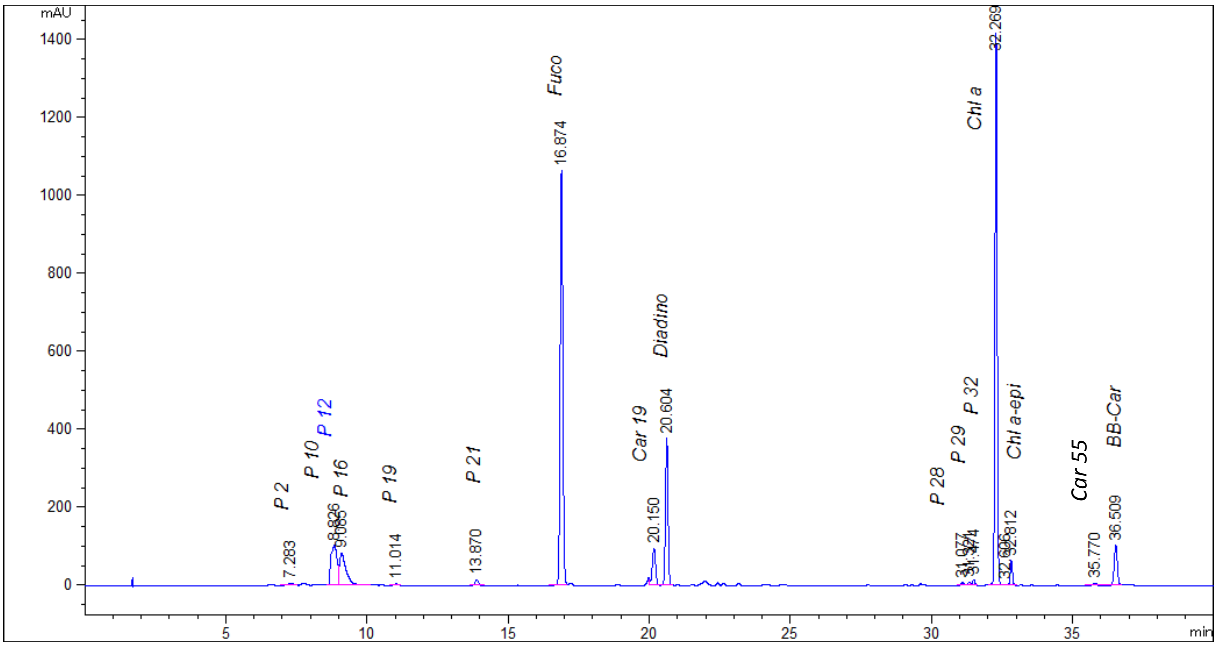

Phaeodactylum tricornutum CCMP 632

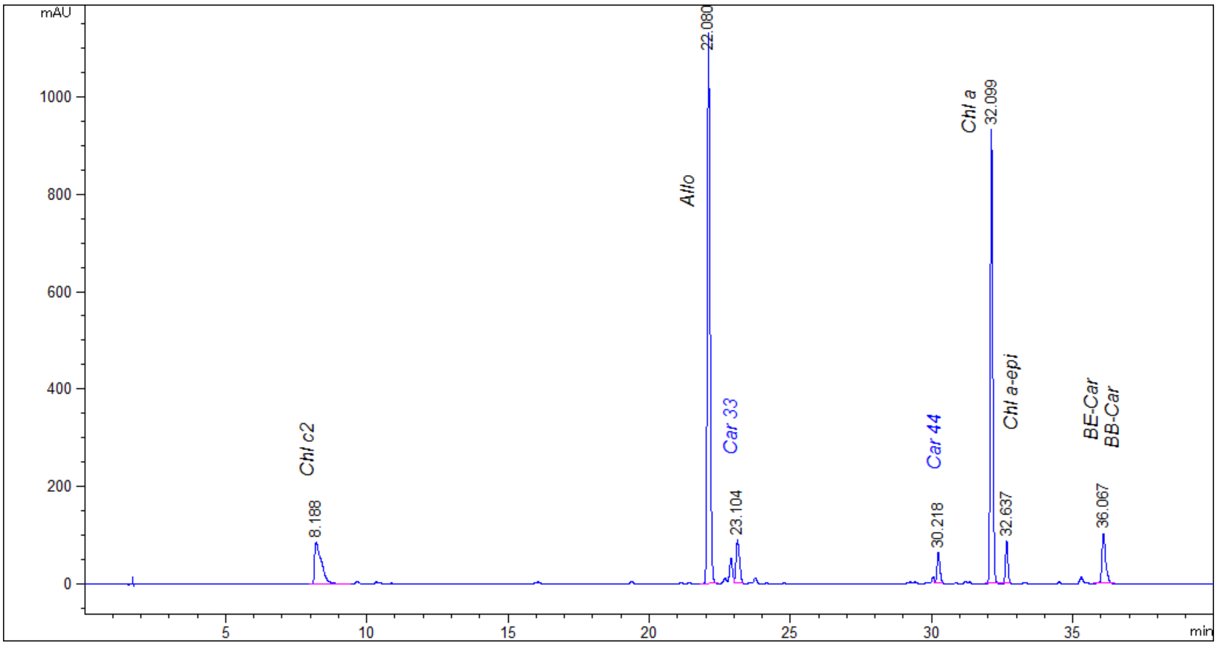

***Rhodomonas salina* CCAP 978/27**

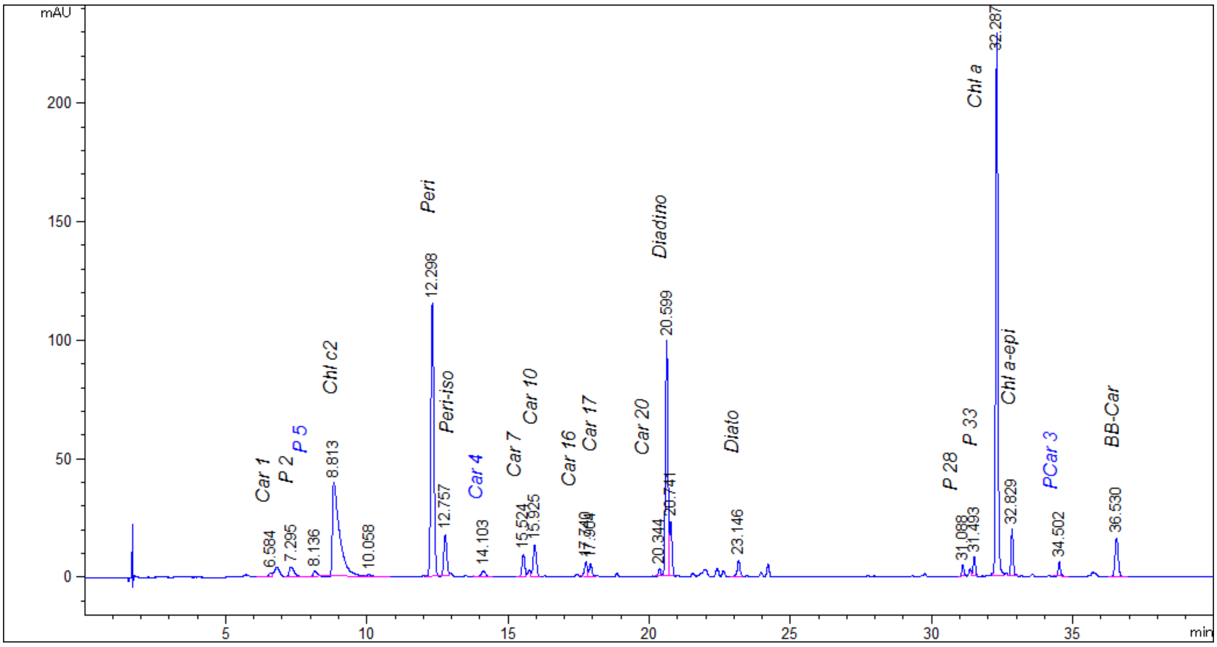

Alexandrium tamarense MOG 835 (toxic)

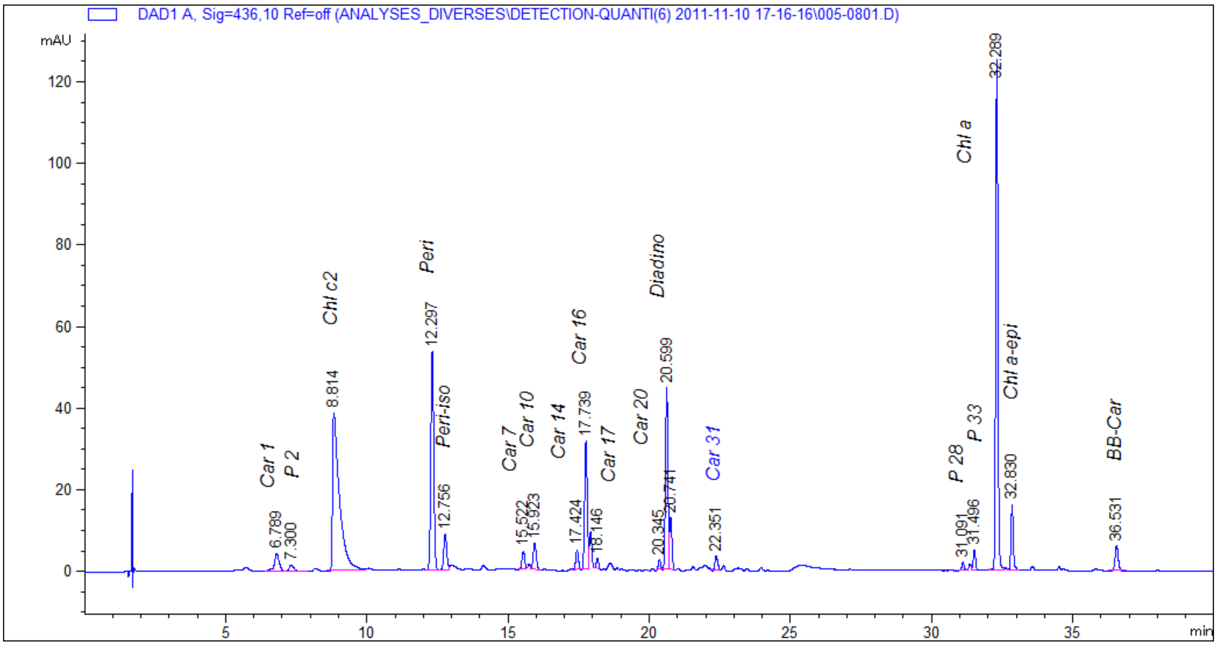

Alexandrium tamarense PLY 497A (non toxic)

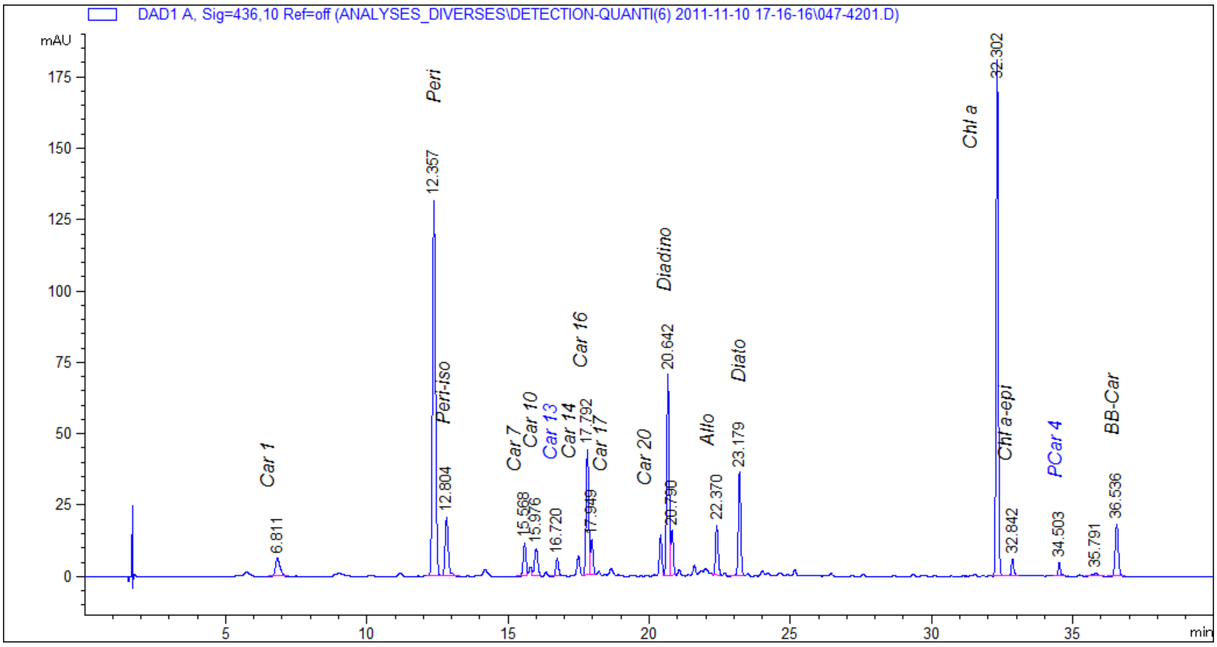

Alexandrium minutum **CM 1002 (non toxic)**

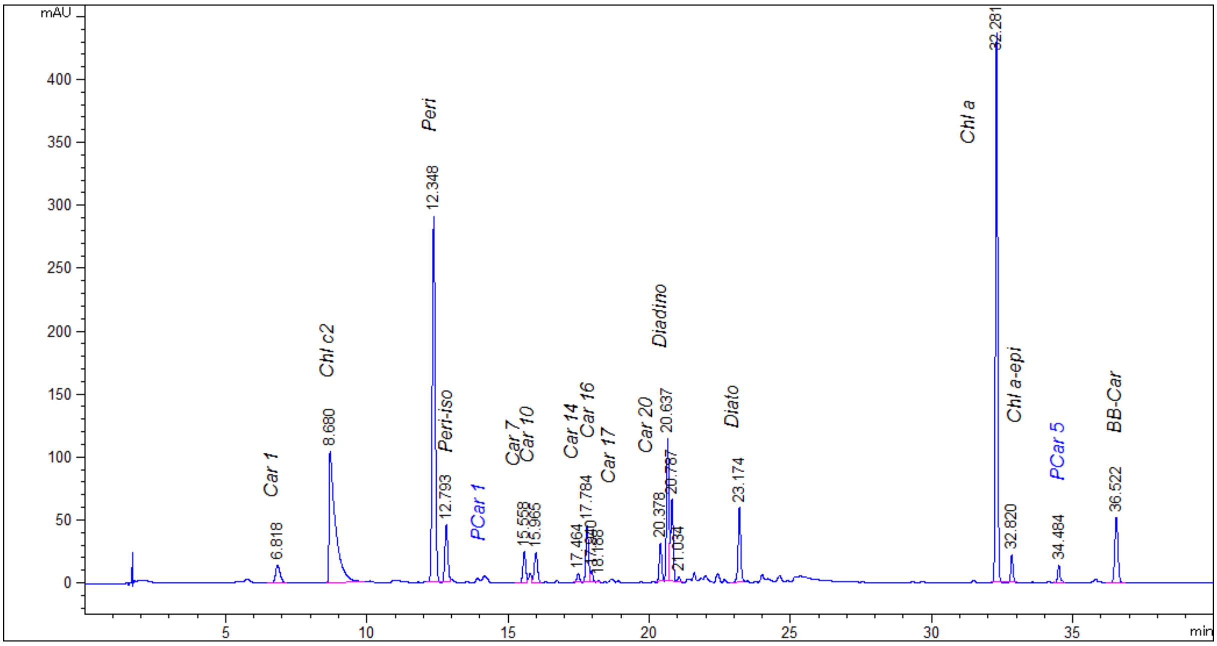

Alexandrium minutum **AM89BM (Toxic)**

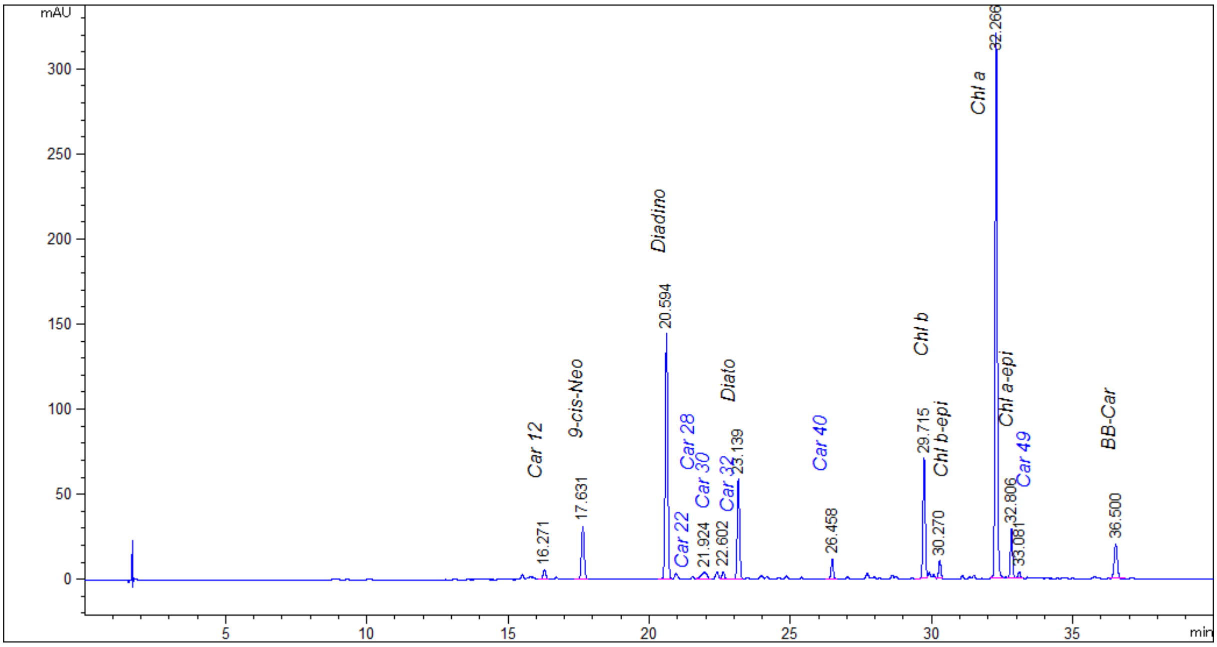

Euglena proxima **SAG 1224-11a**
