# Supplementary material for: Community analysis of pigment patterns from 37 microalgae strains reveals new carotenoids and porphyrins characteristic of distinct strains and taxonomic groups
Source: PLoS One. 2017 Feb 23;12(2):e0171872. doi: 10.1371/journal.pone.0171872 (PMC5322898; doi:10.1371/journal.pone.0171872)
Supplement: S3 Fig — (PDF) [file pone.0171872.s003.pdf]

Supporting Information

S4 Figs. Spectra of unidentified pigments.

P1

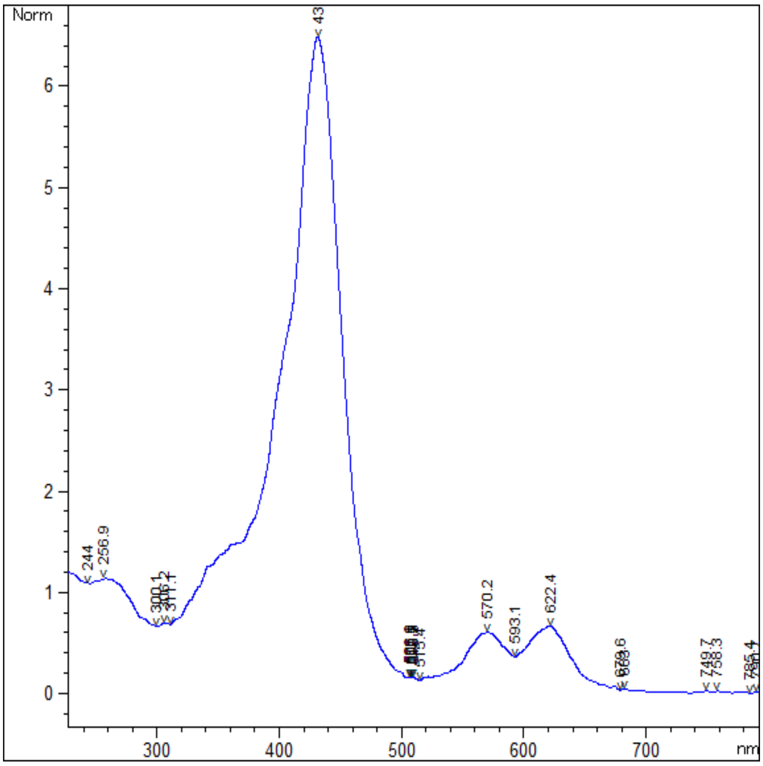

P2

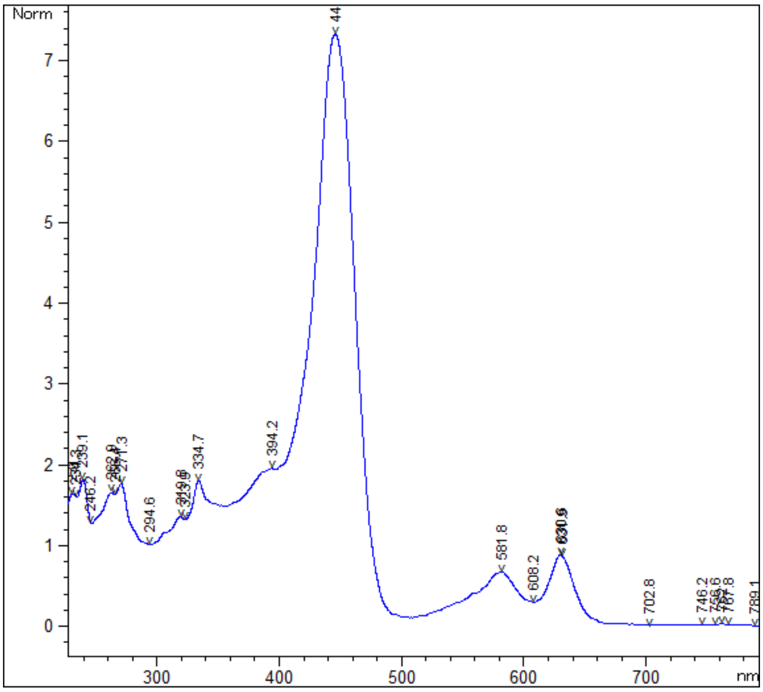

P3

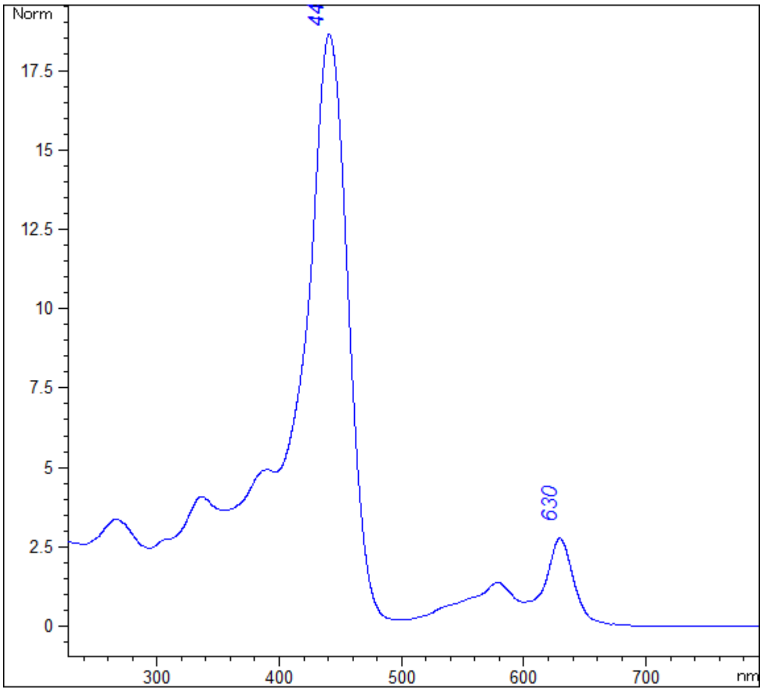

Norm
17.5
15
12.5
10
7.5
5
2.5
0
300
400
500
600
700
nm
44
630

P4

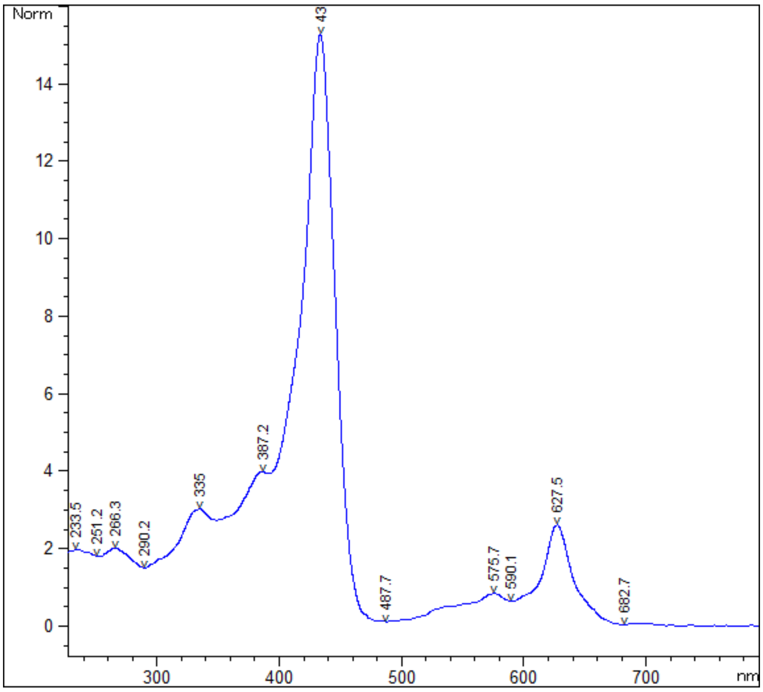

Norm
14
12
10
8
6
4
2
0
300
400
500
600
700
nm
43
387.2
335
233.5
251.2
266.3
290.2
487.7
575.7
590.1
627.5
682.7

P5

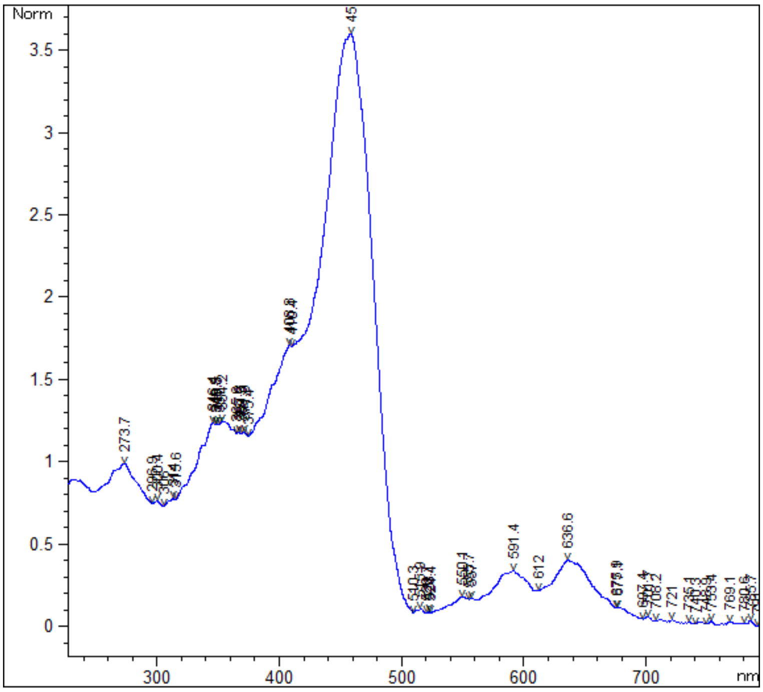

Norm
3.5
3
2.5
2
1.5
1
0.5
0
300
400
500
600
700
nm
45
273.7
591.4
612
636.6
721
769.1

P6

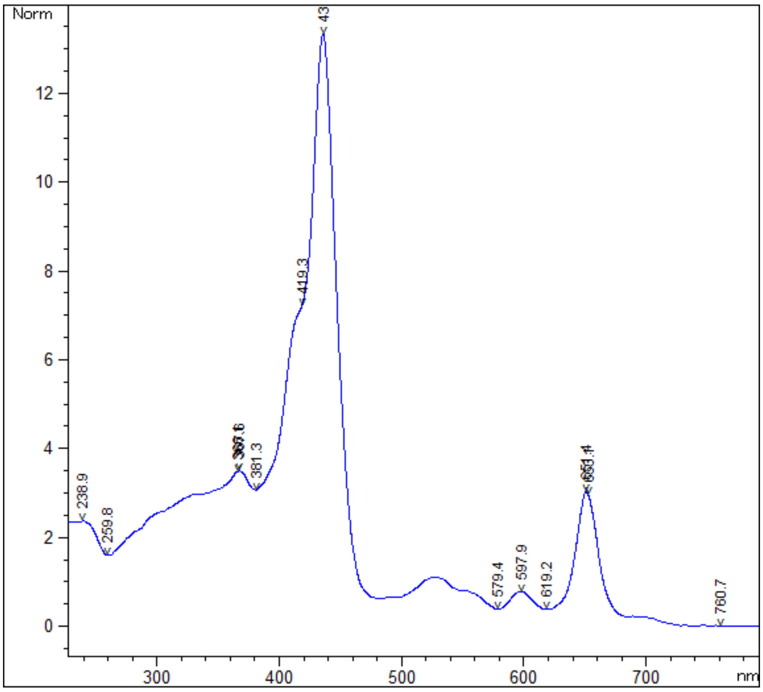

Norm
12
10
8
6
4
2
0
300
400
500
600
700
nm
43
419.3
381.3
238.9
259.8
579.4
597.9
619.2
760.7

P7

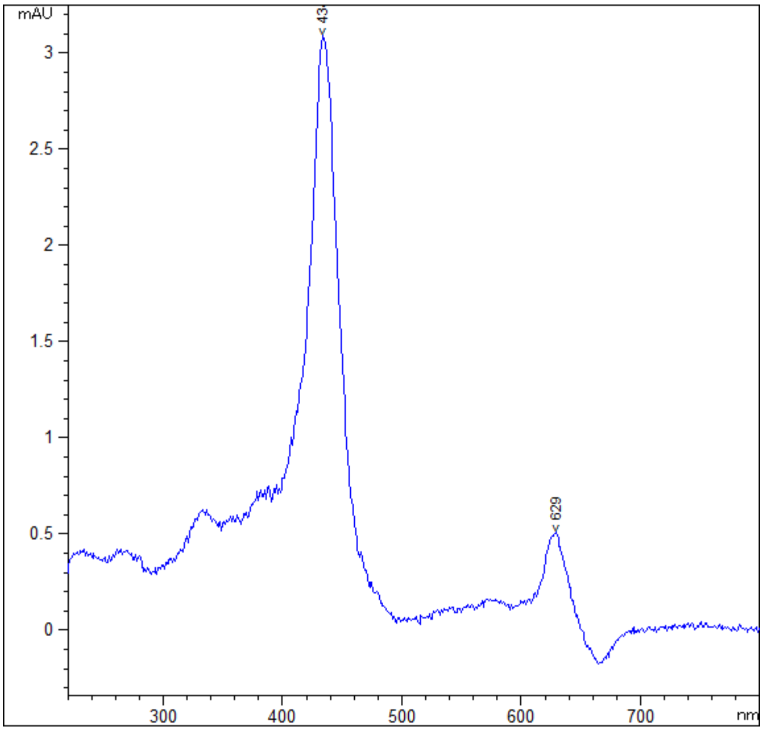
mAU
3
2.5
2
1.5
1
0.5
0
300
400
500
600
700
nm
43
629

P8

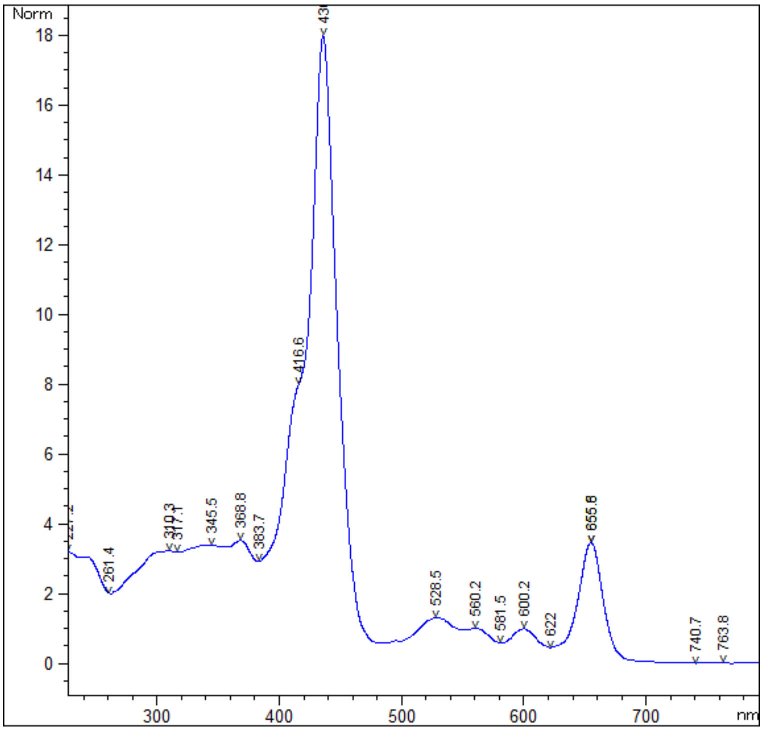
Norm
18
16
14
12
10
8
6
4
2
0
300
400
500
600
700
nm
43
416.6
261.4
310.3
317.1
345.5
368.8
383.7
528.5
560.2
581.5
600.2
622
655.8
740.7
763.8

P9

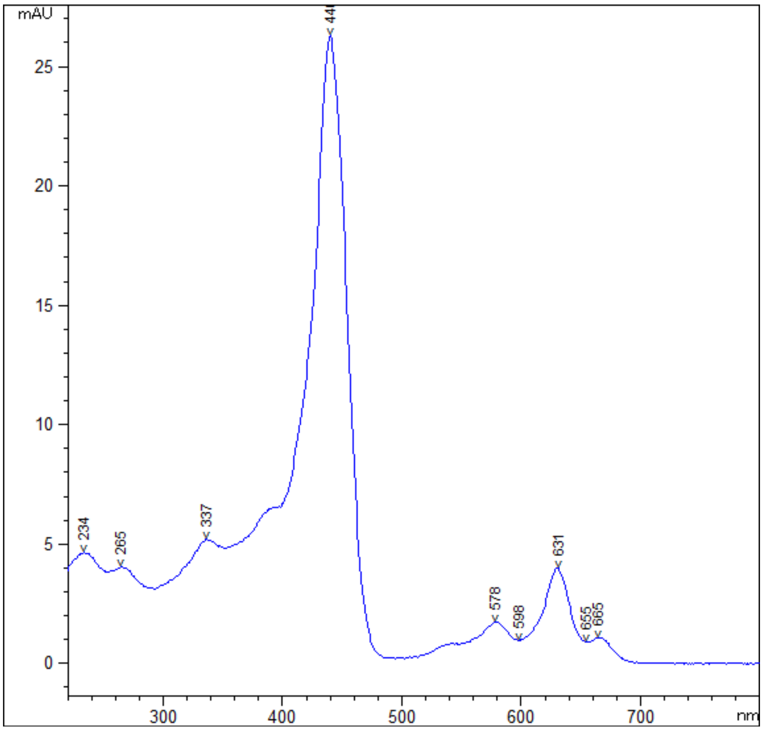

mAU
25
20
15
10
5
0
300
400
500
600
700
nm
440
234
265
337
578
598
631
655
665

P10

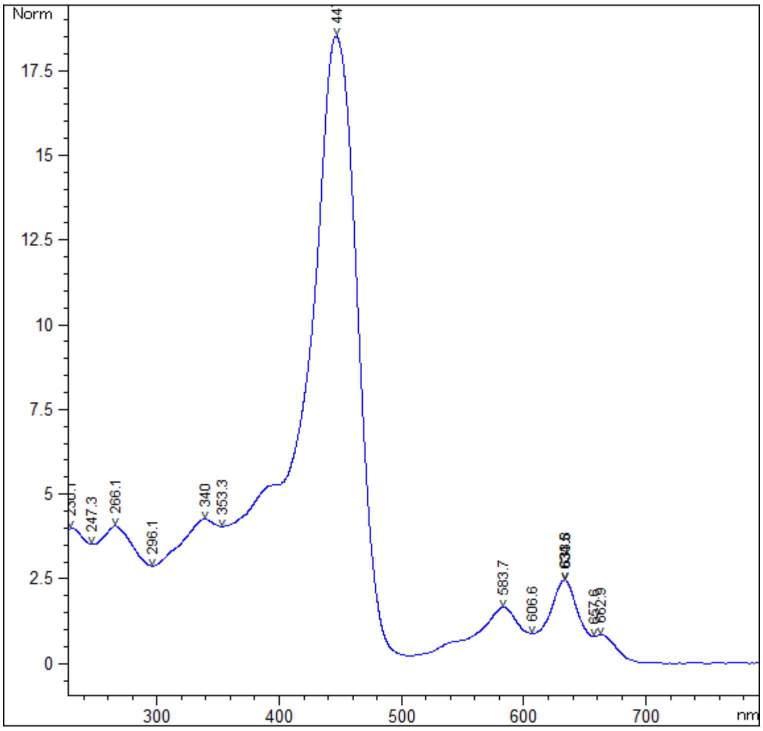

Norm
17.5
15
12.5
10
7.5
5
2.5
0
300
400
500
600
700
nm
447
247.3
266.1
296.1
340
353.3
583.7
606.6

P11

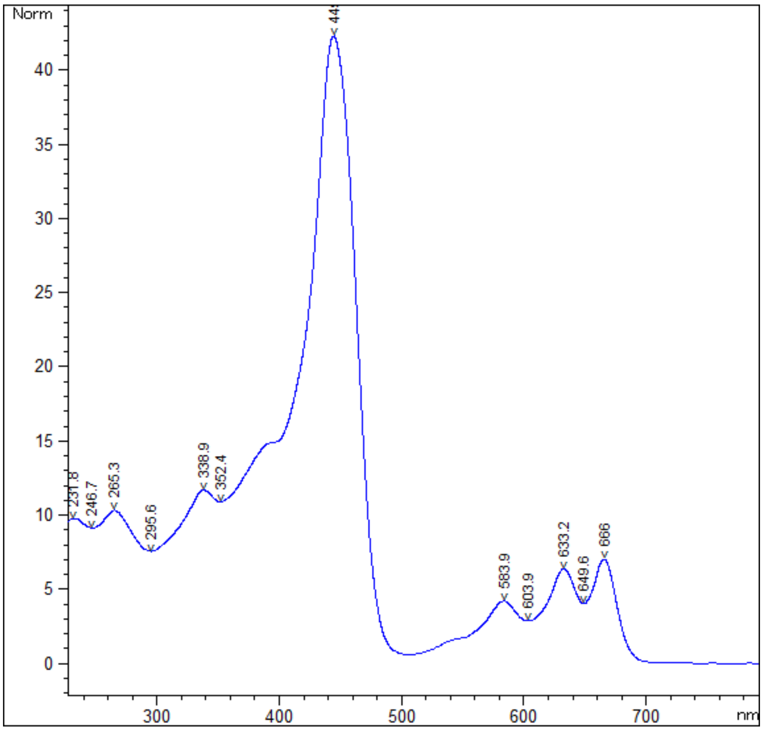
Norm
40
35
30
25
20
15
10
5
0
300
400
500
600
700
nm
231.8
246.7
265.3
295.6
338.9
352.4
44
583.9
603.9
633.2
649.6
666

P12

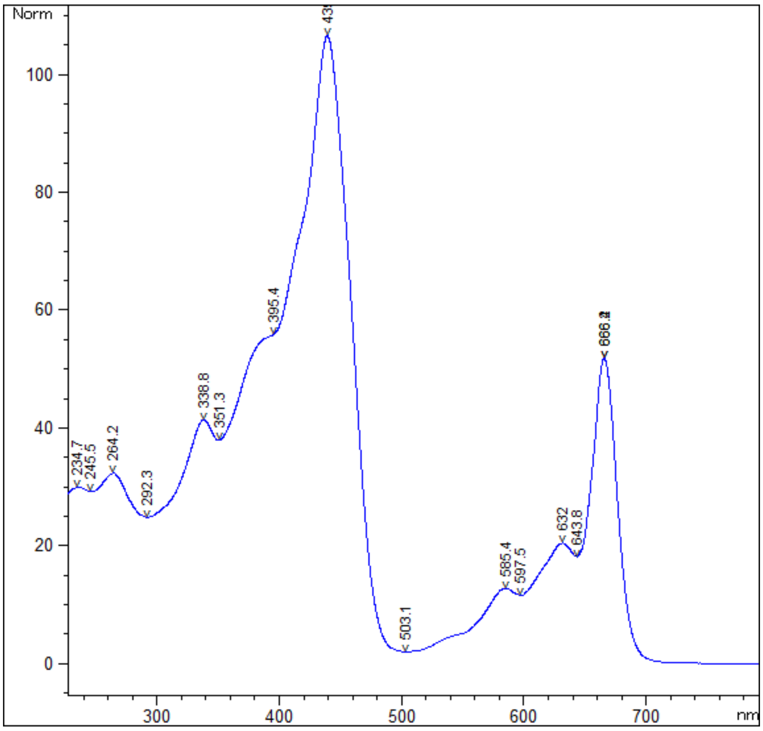
Norm
100
80
60
40
20
0
300
400
500
600
700
nm
234.7
245.5
264.2
292.3
338.8
351.3
395.4
43
503.1
585.4
597.5
632
643.8

P13

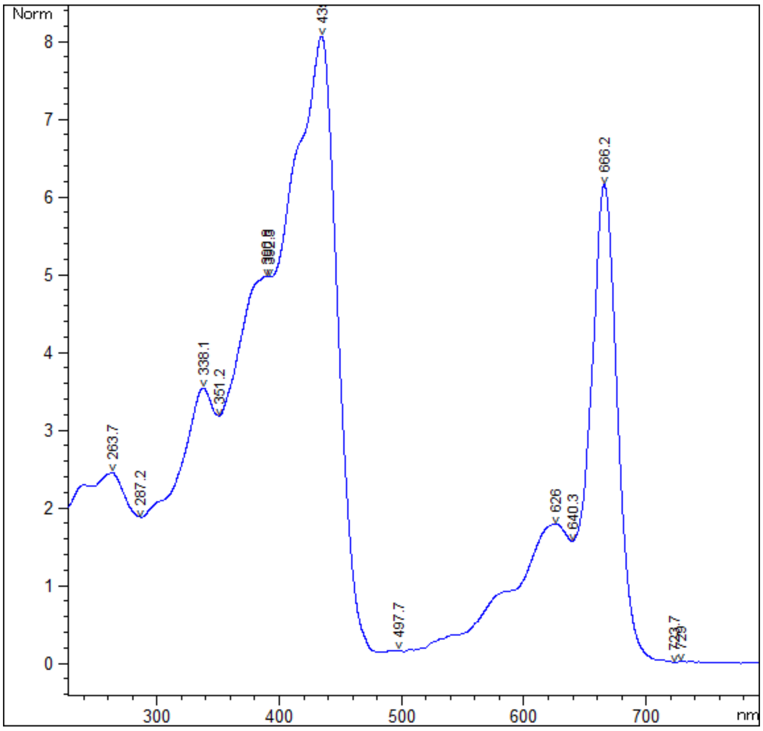
Norm
8
7
6
5
4
3
2
1
0
300
400
500
600
700
nm
263.7
287.2
338.1
351.2
43
497.7
626
640.3
666.2
723.7
729

P14

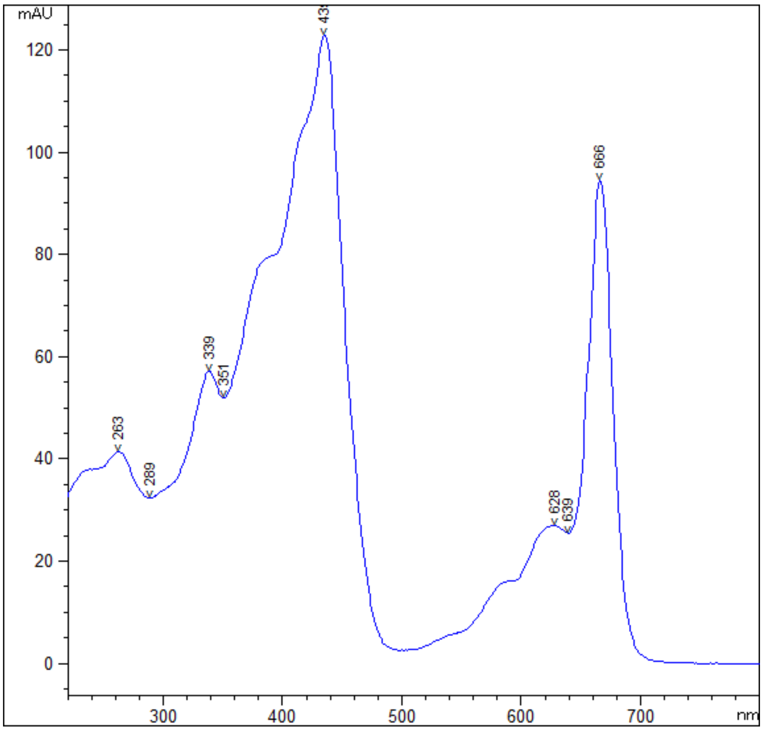
mAU
120
100
80
60
40
20
0
300
400
500
600
700
nm
263
289
339
351
43
628
639
666

P15

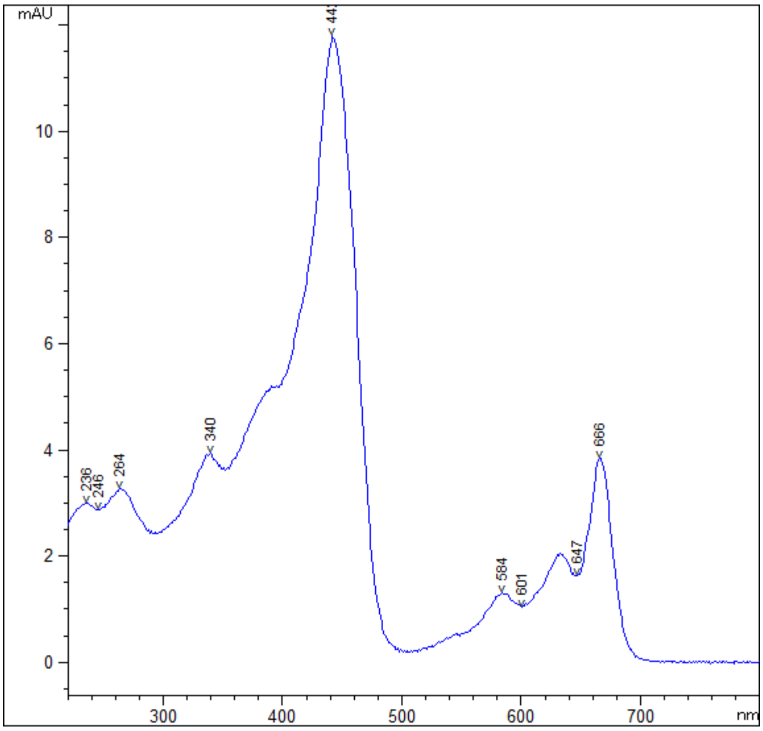
mAU
441
10
8
6
4
2
0
340
236
246
264
584
601
647
666
300
400
500
600
700
nm

P16

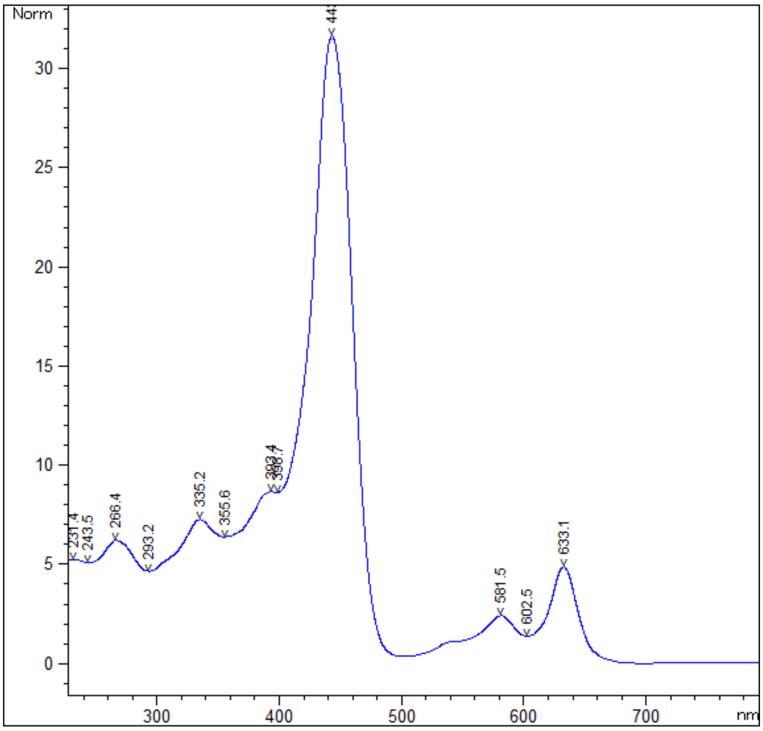
Norm
441
30
25
20
15
10
5
0
231.4
243.5
266.4
293.2
335.2
355.6
581.5
602.5
633.1
300
400
500
600
700
nm

P17

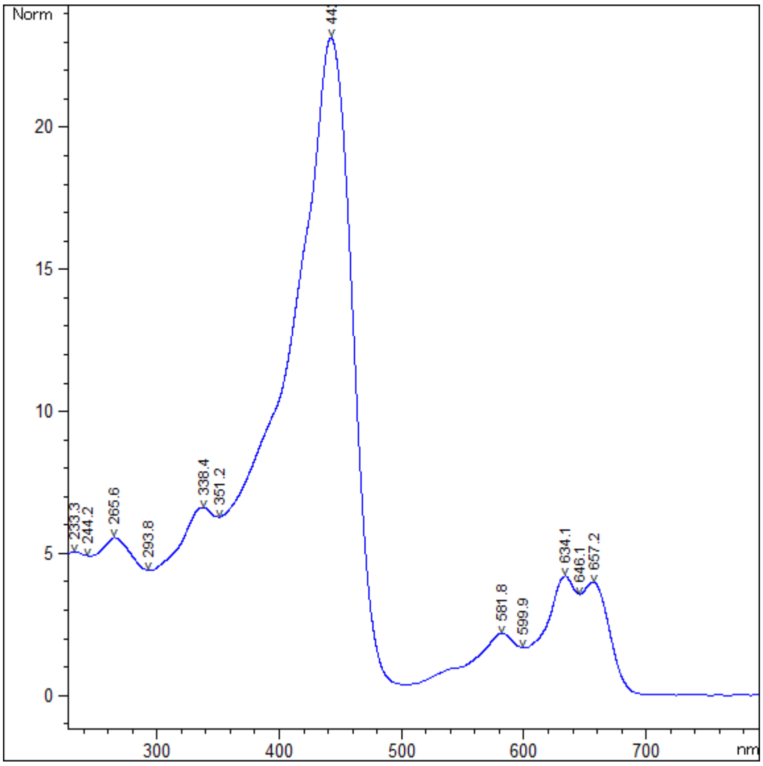
Norm
20
15
10
5
0
233.3
244.2
265.6
293.8
338.4
351.2
581.8
599.9
634.1
646.1
657.2
300
400
500
600
700
nm

P18

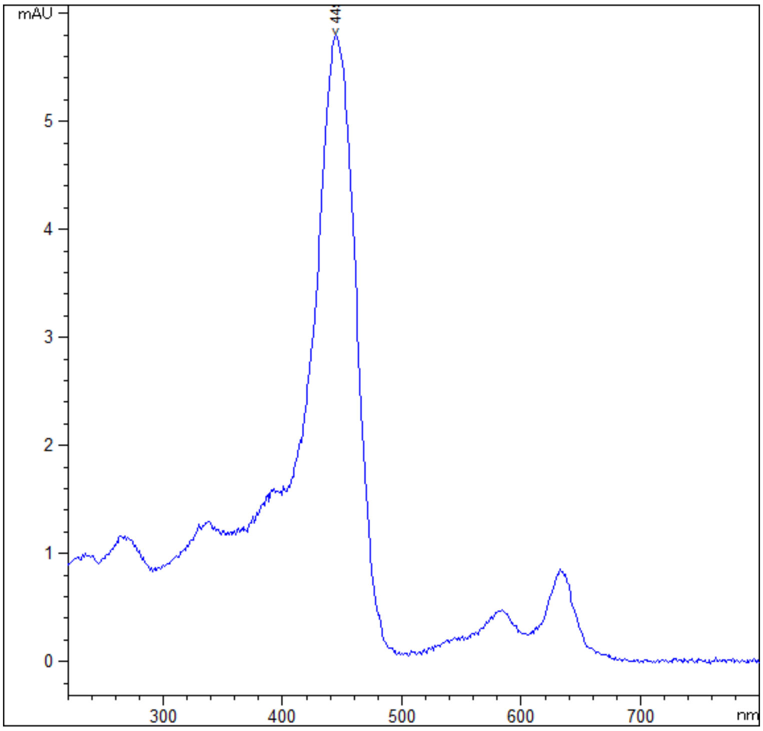
mAU
5
4
3
2
1
0
300
400
500
600
700
nm

P19

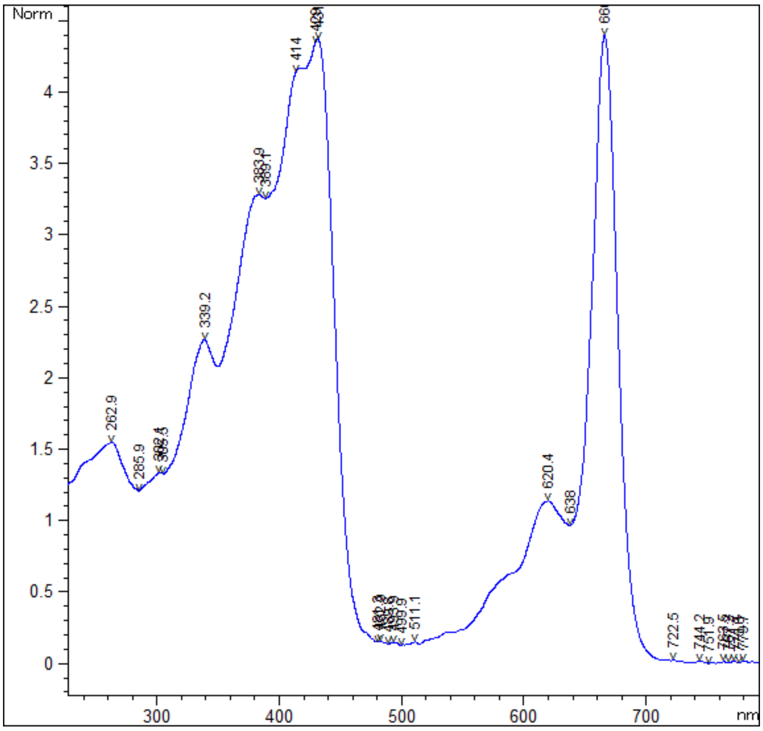
Norm
4
3.5
3
2.5
2
1.5
1
0.5
0
300
400
500
600
700
nm
262.9
285.9
339.2
383.9
389.1
414
620.4
638
511.1
722.5
744.2
751.9

P20

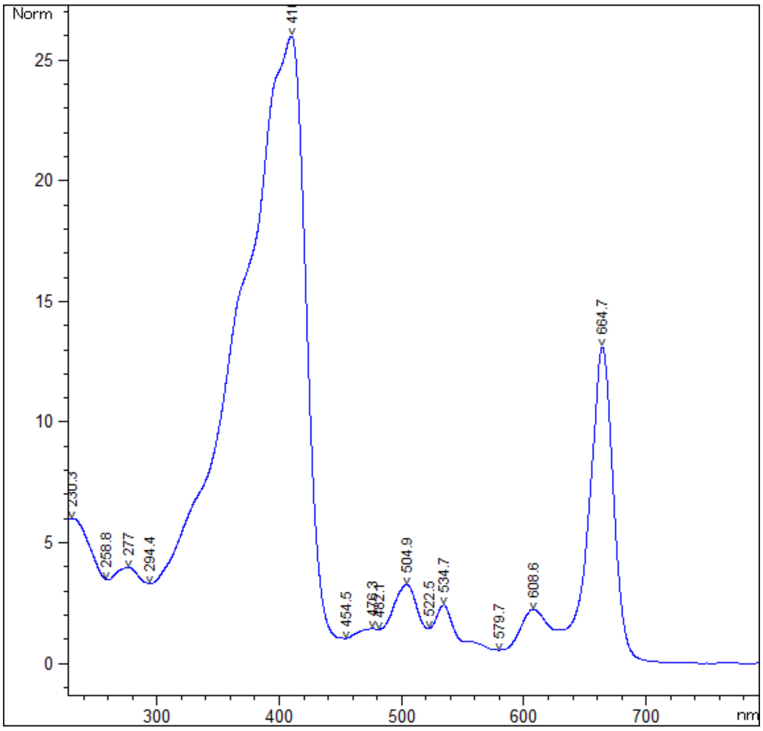
Norm
25
20
15
10
5
0
300
400
500
600
700
nm
258.8
277
294.4
454.5
482.1
504.9
522.5
534.7
579.7
608.6
664.7

P21

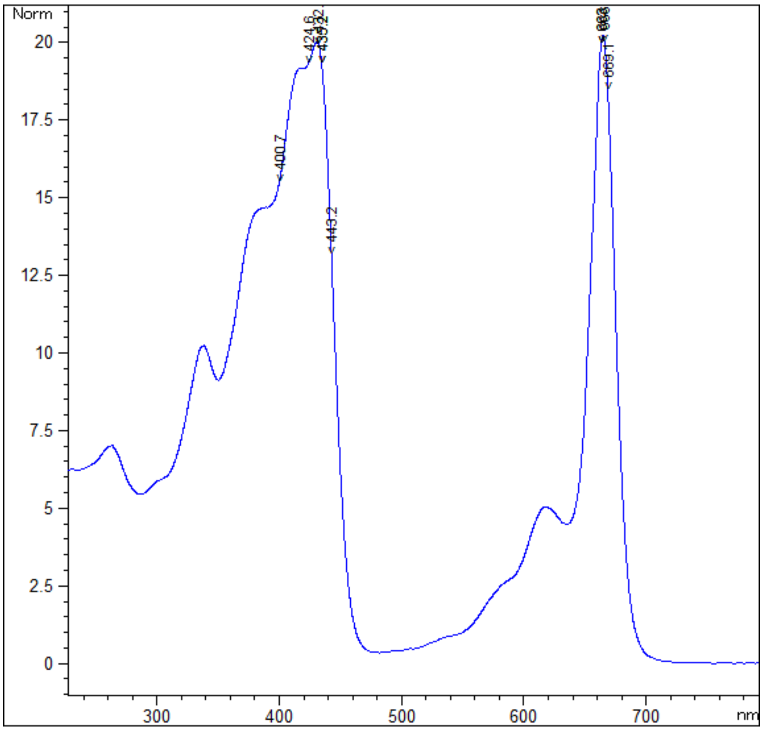
Norm
20
17.5
15
12.5
10
7.5
5
2.5
0
300
400
500
600
700
nm
424.6
400.7
443.2
669.1

P22

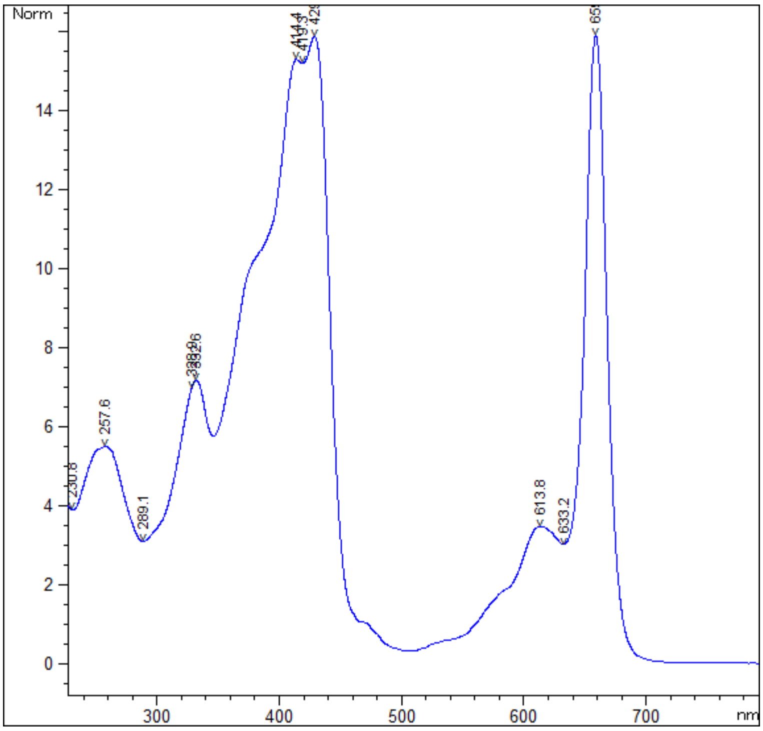
Norm
14
12
10
8
6
4
2
0
300
400
500
600
700
nm
230.8
257.6
289.1
613.8
633.2

P23

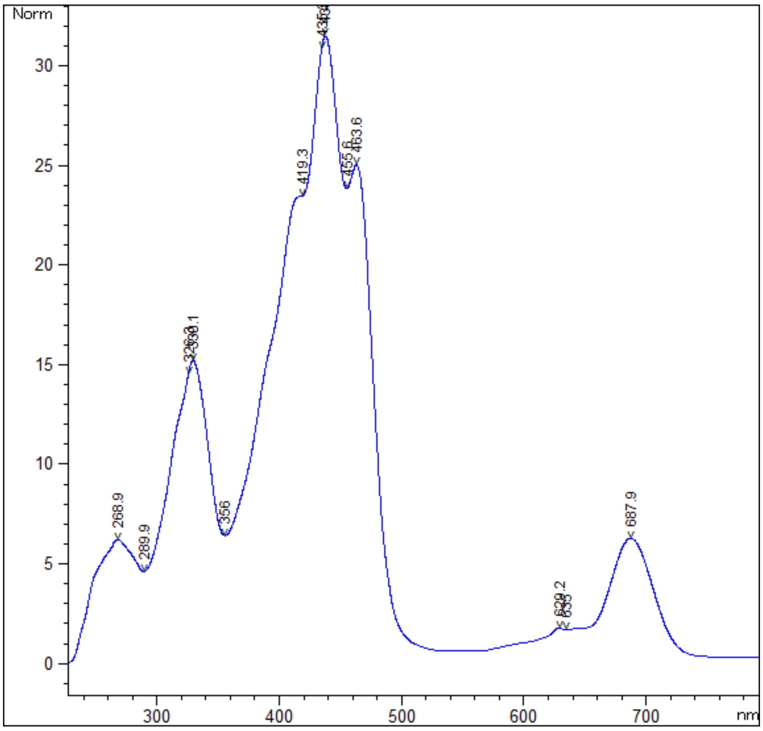
Norm
268.9
289.9
356
419.3
455.6
463.6
687.9
nm

P24

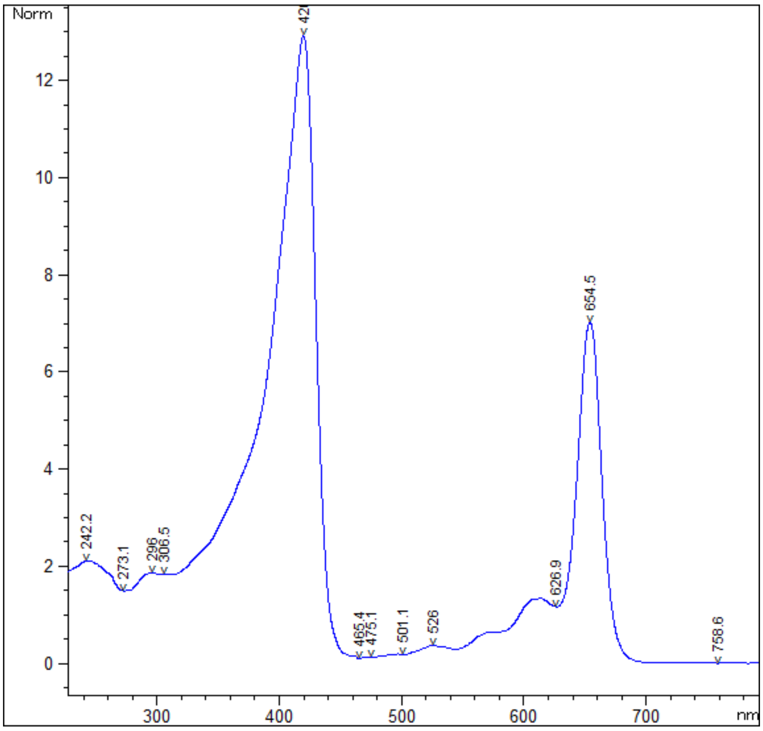
Norm
242.2
273.1
296
306.5
465.4
475.1
501.1
526
626.9
654.5
758.6
nm

P25

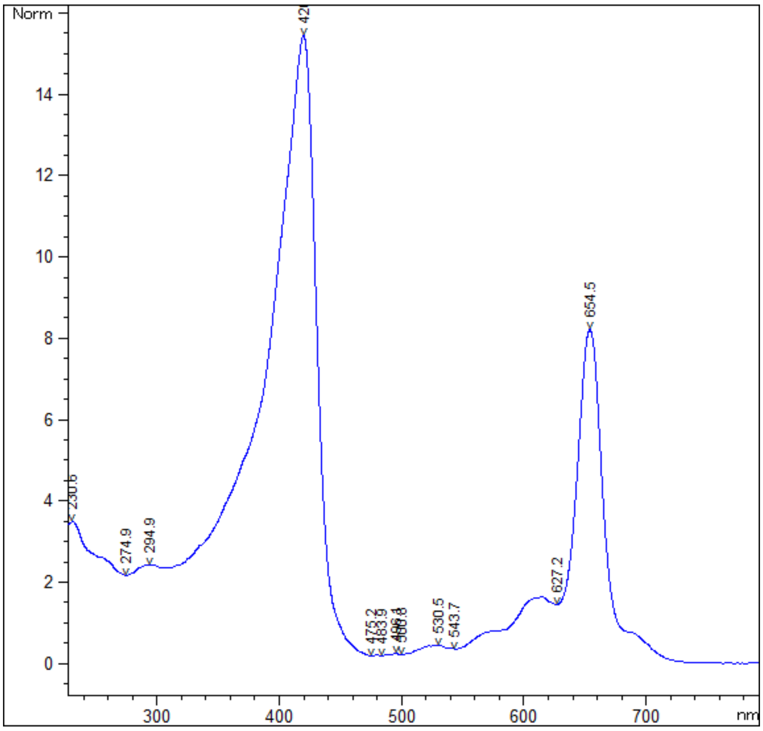

Norm
14
12
10
8
6
4
2
0
300
400
500
600
700
nm
230.5
274.9
294.9
42
475.2
483.9
530.5
543.7
627.2
654.5

P26

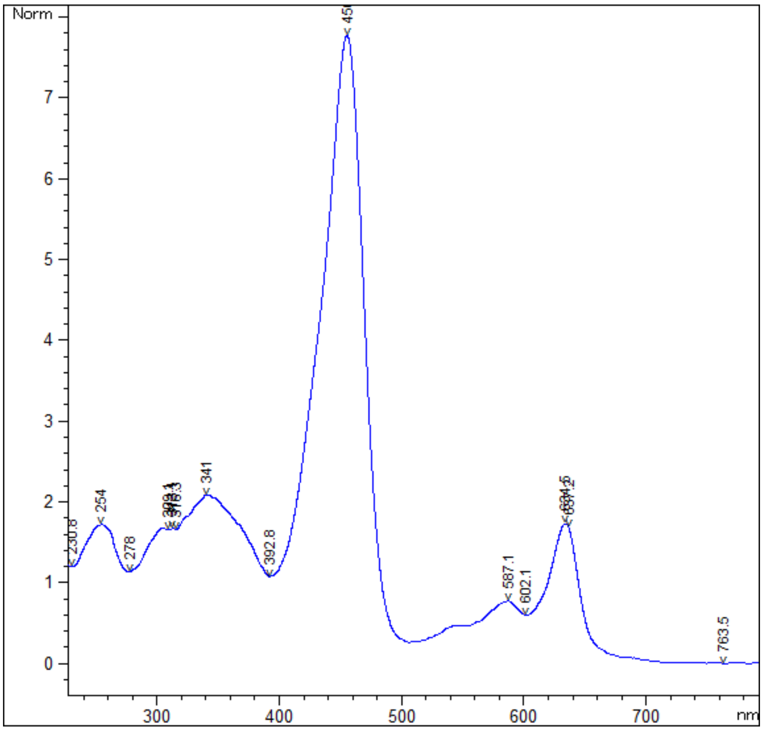

Norm
7
6
5
4
3
2
1
0
300
400
500
600
700
nm
230.8
254
278
341
392.8
45
587.1
602.1
763.5

P27

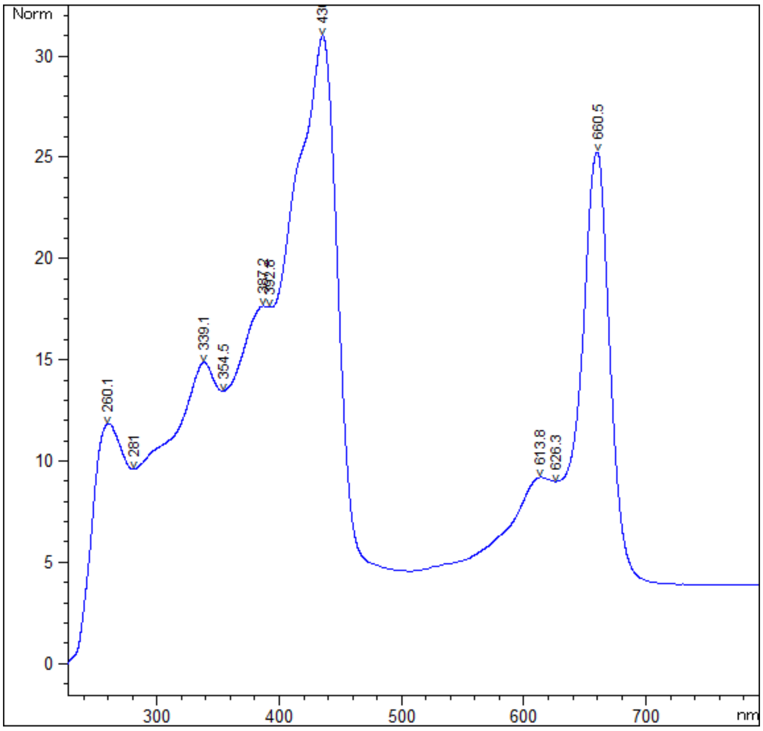

Norm
30
25
20
15
10
5
0
300
400
500
600
700
nm
260.1
281
339.1
354.5
387.2
392.8
660.5
613.8
626.3

P28

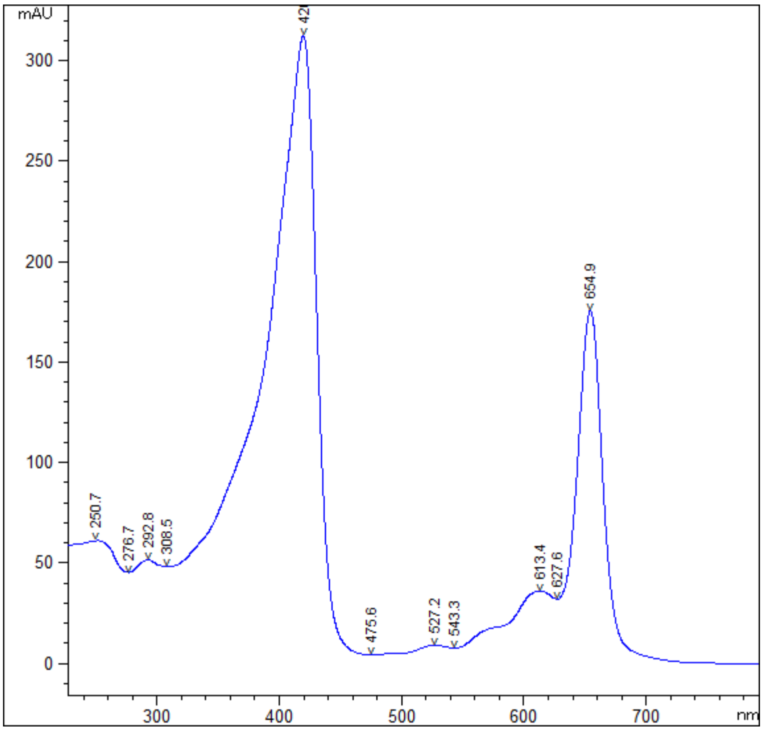

mAU
300
250
200
150
100
50
0
300
400
500
600
700
nm
250.7
276.7
292.8
308.5
475.6
527.2
543.3
613.4
627.6
654.9

P29

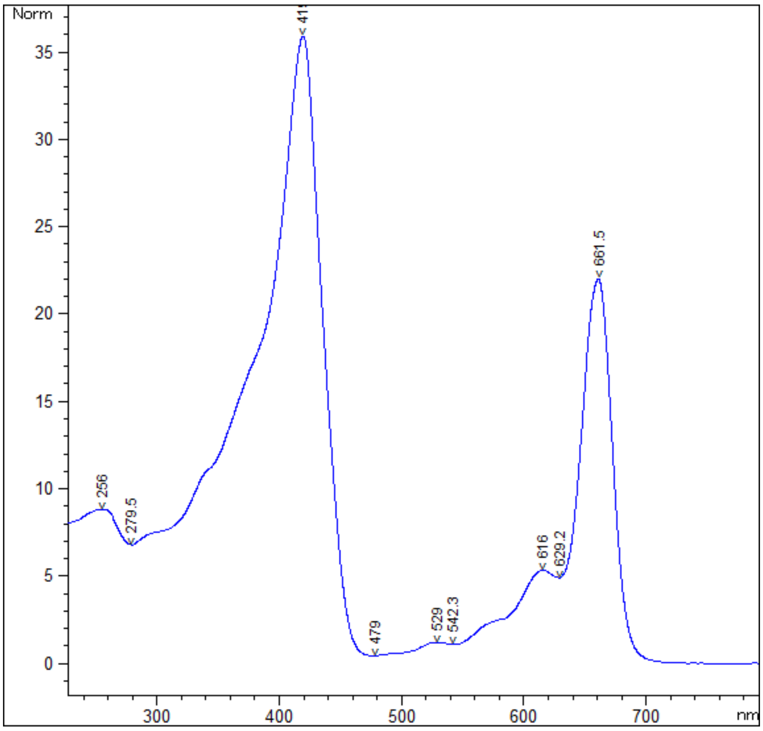
Norm
35
30
25
20
15
10
5
0
300
400
500
600
700
nm
256
279.5
479
529
542.3
616
629.2
661.5

P30

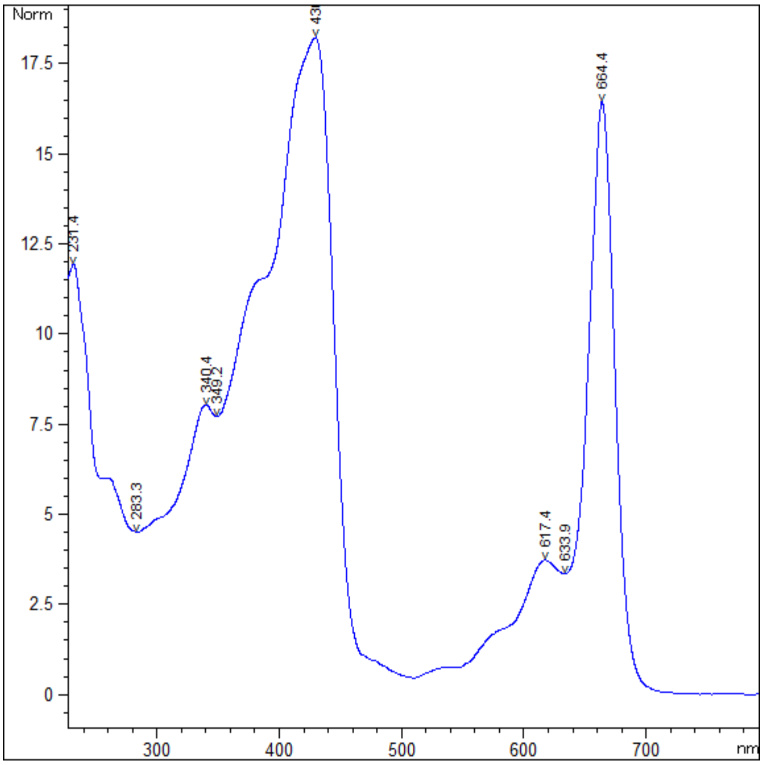
Norm
17.5
15
12.5
10
7.5
5
2.5
0
300
400
500
600
700
nm
231.4
283.3
340.4
349.2
617.4
633.9
664.4

P31

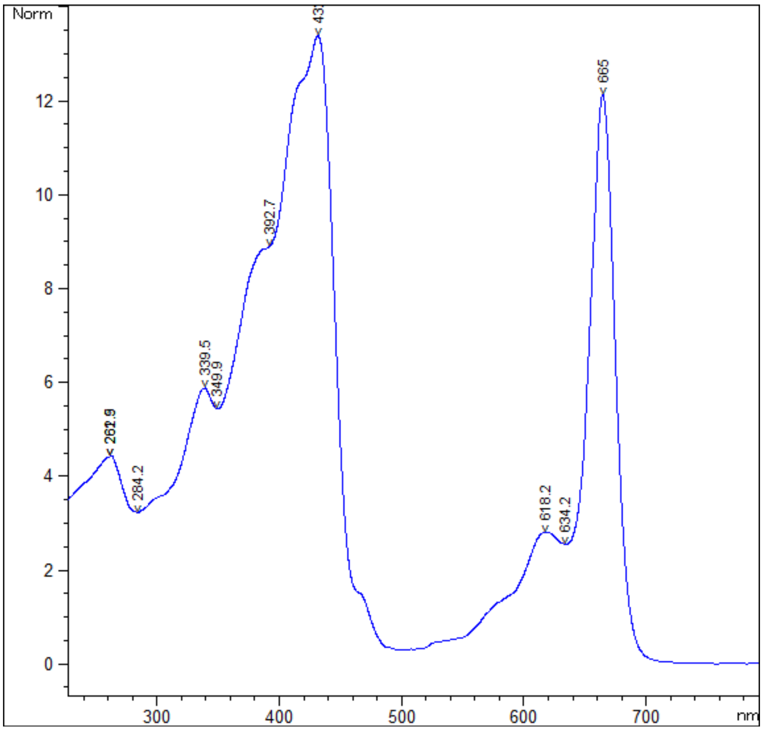
Norm
12
10
8
6
4
2
0
300
400
500
600
700
nm
392.7
339.5
349.9
284.2
665
618.2
634.2

P32

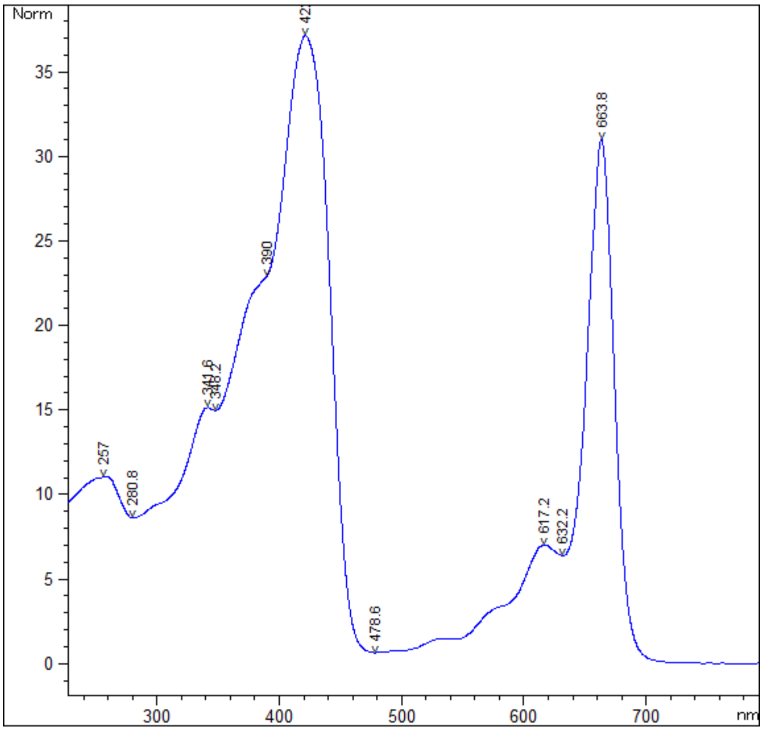
Norm
35
30
25
20
15
10
5
0
300
400
500
600
700
nm
663.8
390
257
280.8
478.6
617.2
632.2

P33

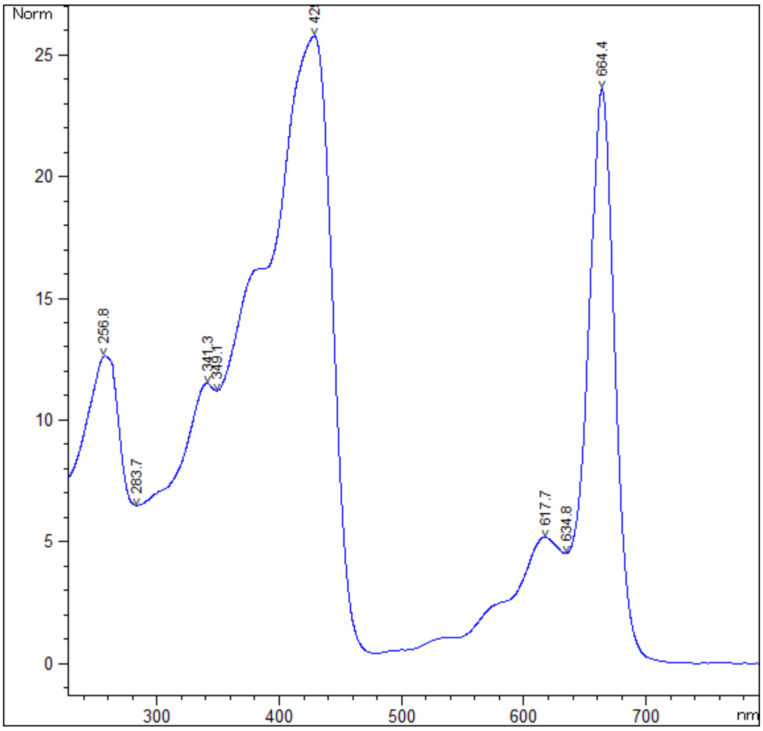
Norm
25
20
15
10
5
0
300
400
500
600
700
nm
256.8
283.7
341.3
349.1
42
664.4
617.7
634.8

P34

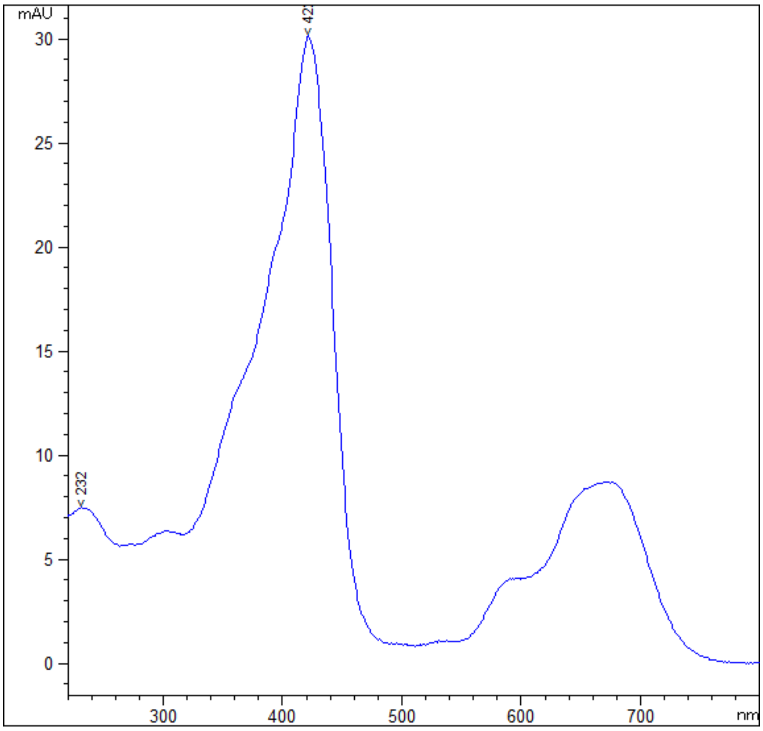
mAU
30
25
20
15
10
5
0
300
400
500
600
700
nm
42
232

P35

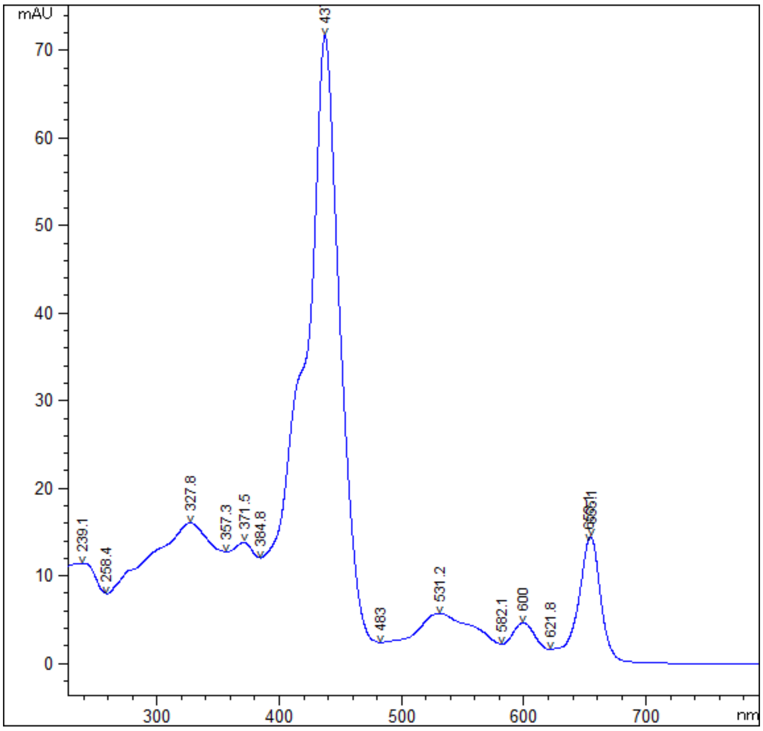
mAU
70
60
50
40
30
20
10
0
300
400
500
600
700
nm
239.1
258.4
327.8
357.3
371.5
384.8
483
531.2
582.1
600
621.8

PCar1

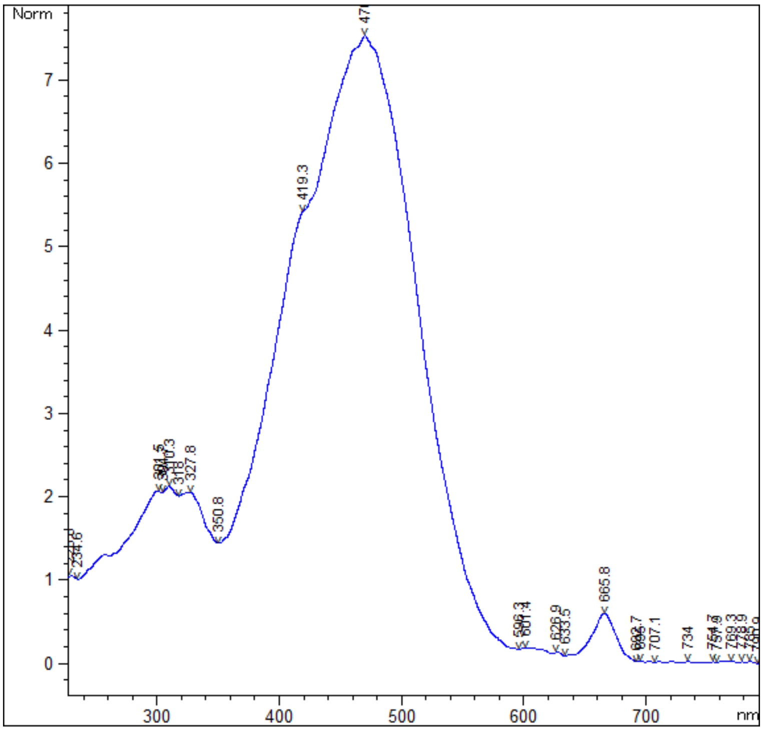
Norm
7
6
5
4
3
2
1
0
300
400
500
600
700
nm
234.6
327.8
350.8
419.3
665.8
707.1
734

PCar 2

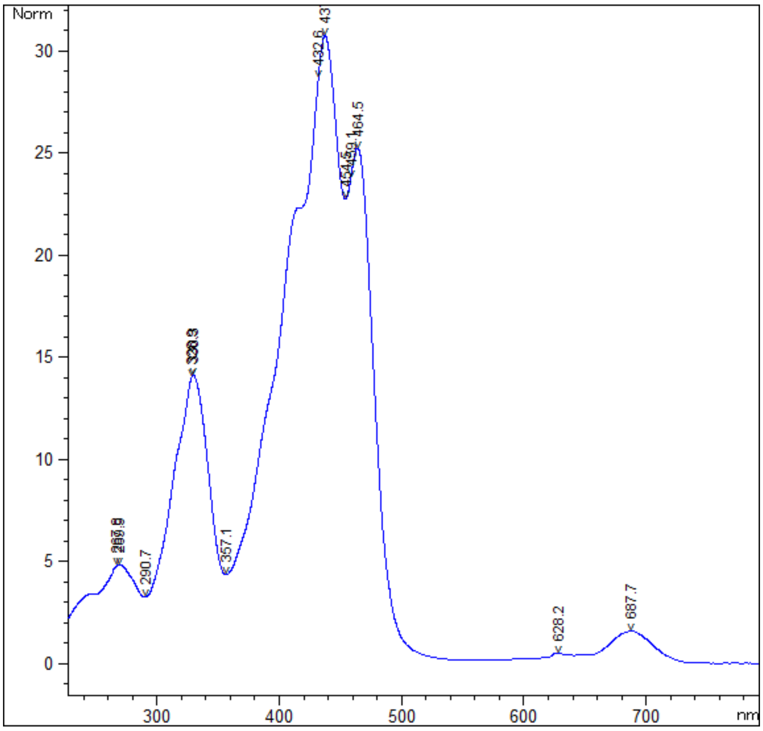
Norm
30
25
20
15
10
5
0
300
400
500
600
700
nm
432.6
464.5
357.1
290.7
628.2
687.7

PCar3

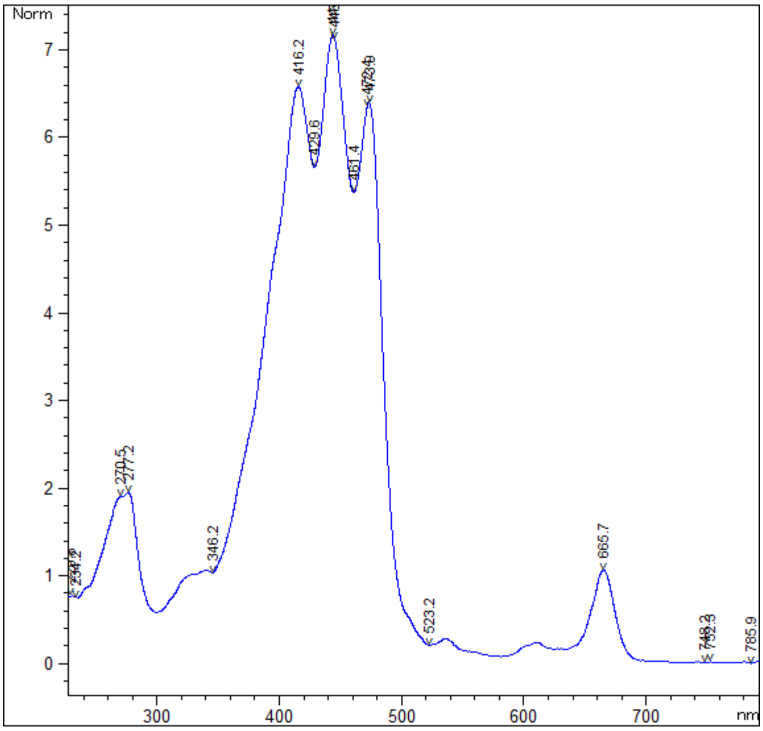
Norm
7
6
5
4
3
2
1
0
300
400
500
600
700
nm
416.2
429.6
461.4
270.5
277.2
346.2
523.2
665.7
785.9

PCar4

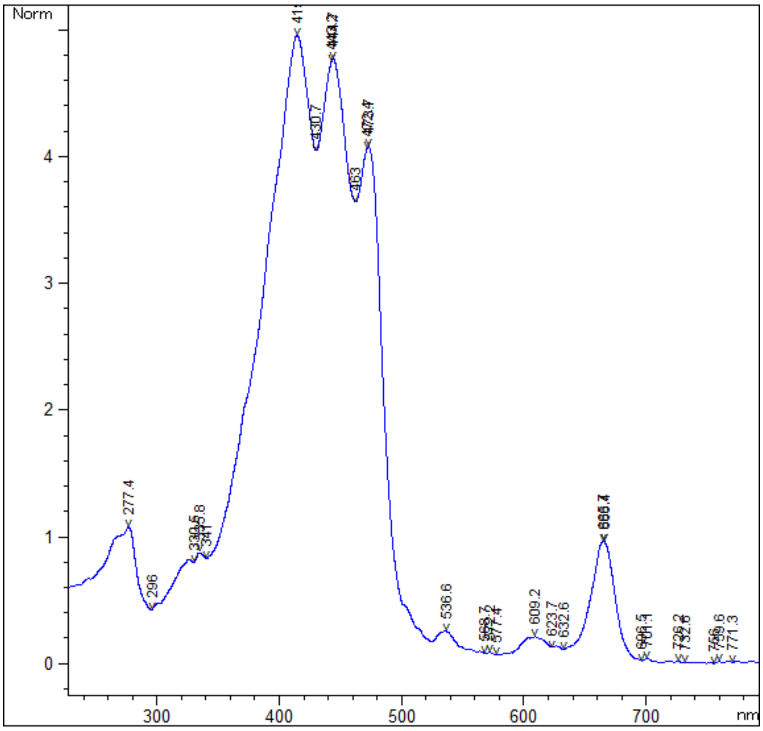
Norm
0
1
2
3
4
300
400
500
600
700
nm
277.4
296
430.7
463
536.6
609.2
623.7
632.6
771.3

PCar5

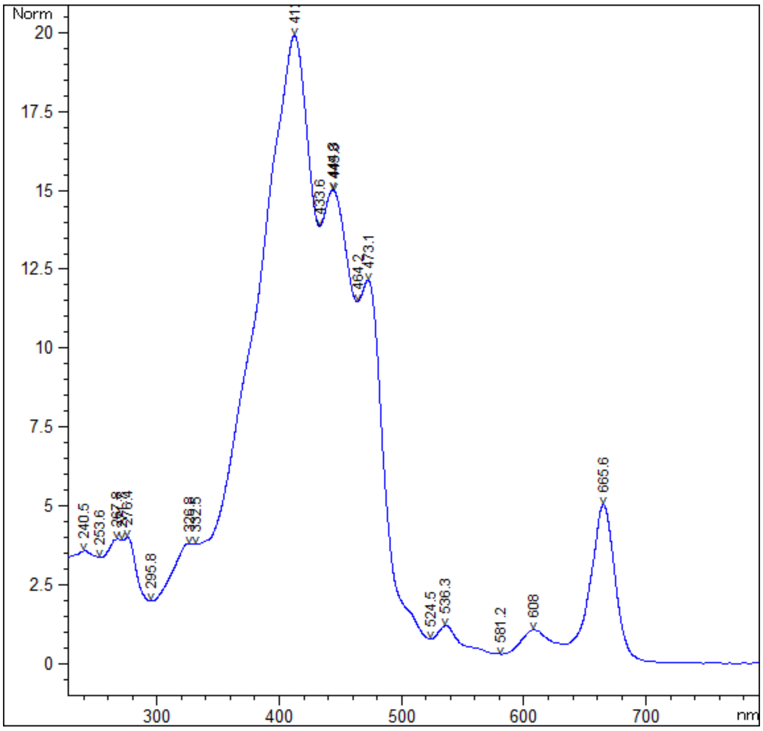
Norm
20
17.5
15
12.5
10
7.5
5
2.5
0
300
400
500
600
700
nm
240.5
253.6
295.8
433.6
464.2
473.1
524.5
536.3
581.2
608
665.6

PCar6

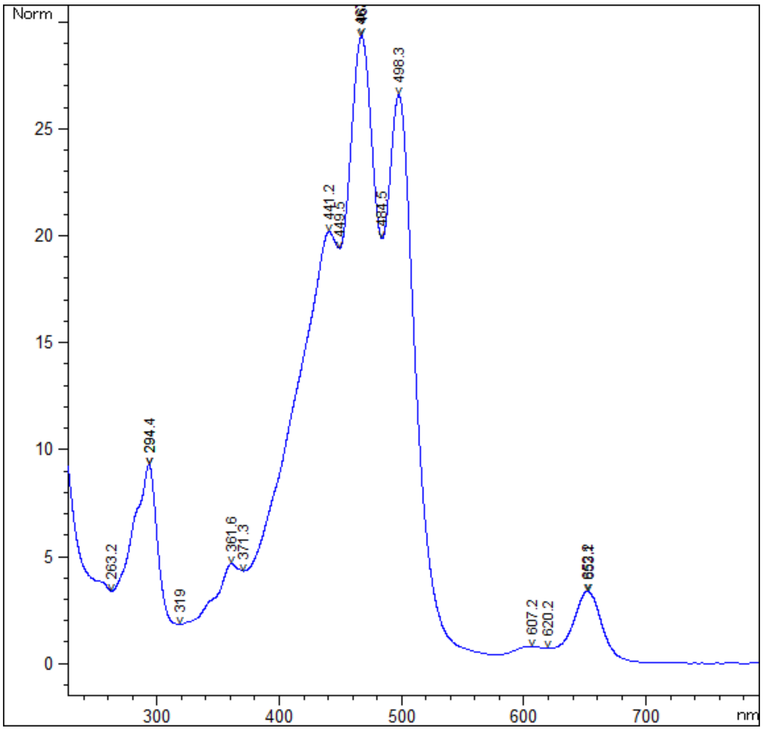
Norm
25
20
15
10
5
0
300
400
500
600
700
nm
263.2
294.4
319
361.6
371.3
441.2
449.5
484.5
498.3
607.2
620.2

Car1

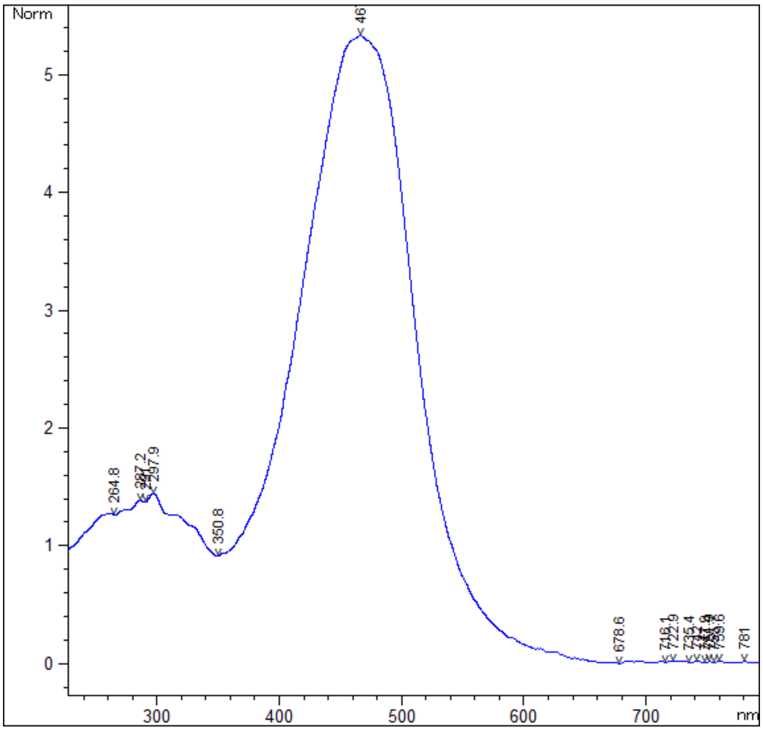
Norm
5
4
3
2
1
0
300
400
500
600
700
nm
264.8
297.9
350.8
678.6
781

Car2

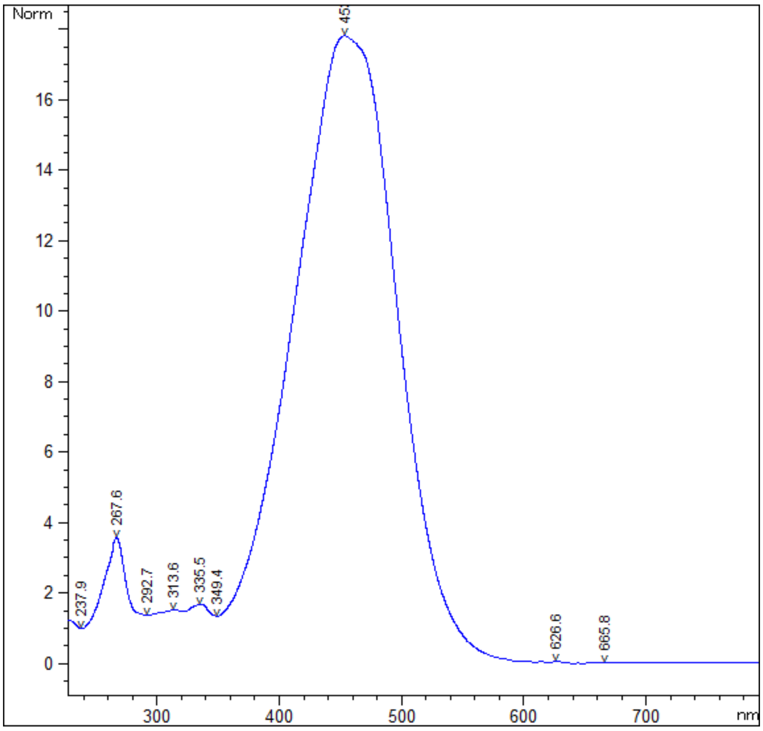
Norm
16
14
12
10
8
6
4
2
0
300
400
500
600
700
nm
237.9
267.6
292.7
313.6
335.5
349.4
626.6
665.8

Car3

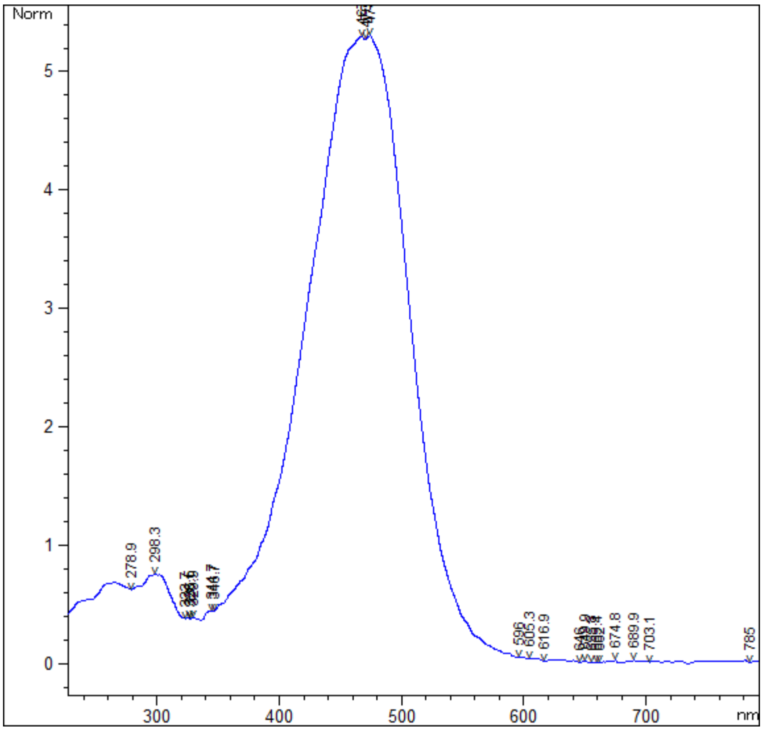
Norm
5
4
3
2
1
0
300
400
500
600
700
nm
278.9
298.3
596
605.3
616.9
674.8
689.9
703.1
785

Car4

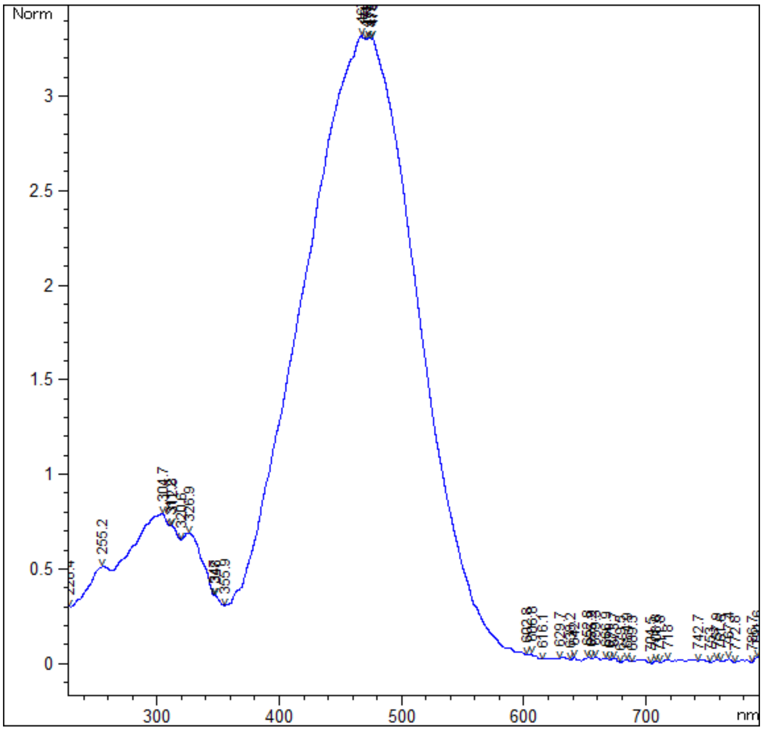
Norm
3
2.5
2
1.5
1
0.5
0
300
400
500
600
700
nm
255.2
304.7
326.9
355.9
616.1
629.7
718
742.7
772.8

Car5

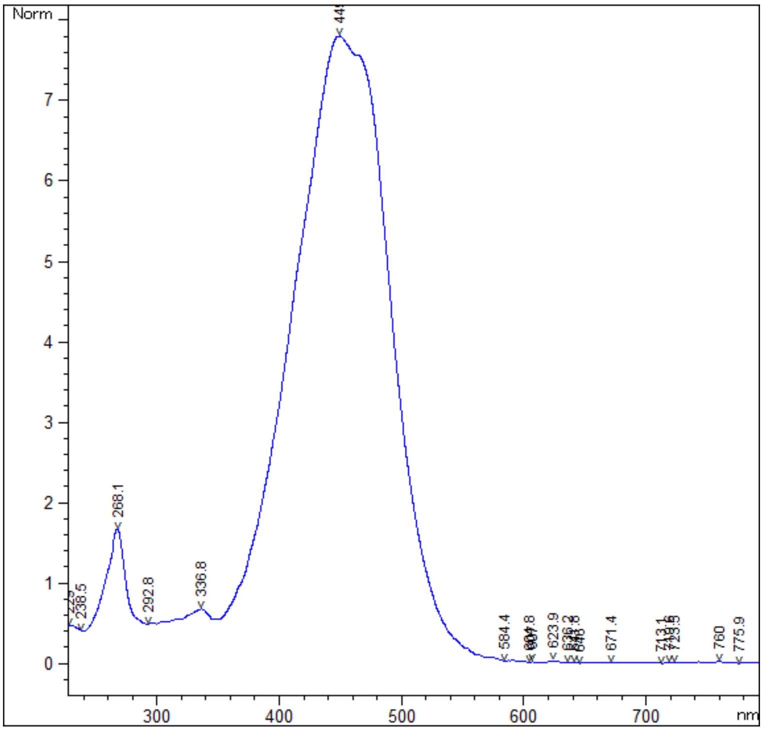

Norm
268.1
292.8
336.8
238.5
584.4
623.9
671.4
713.1
760
775.9
300
400
500
600
700
nm

Car6

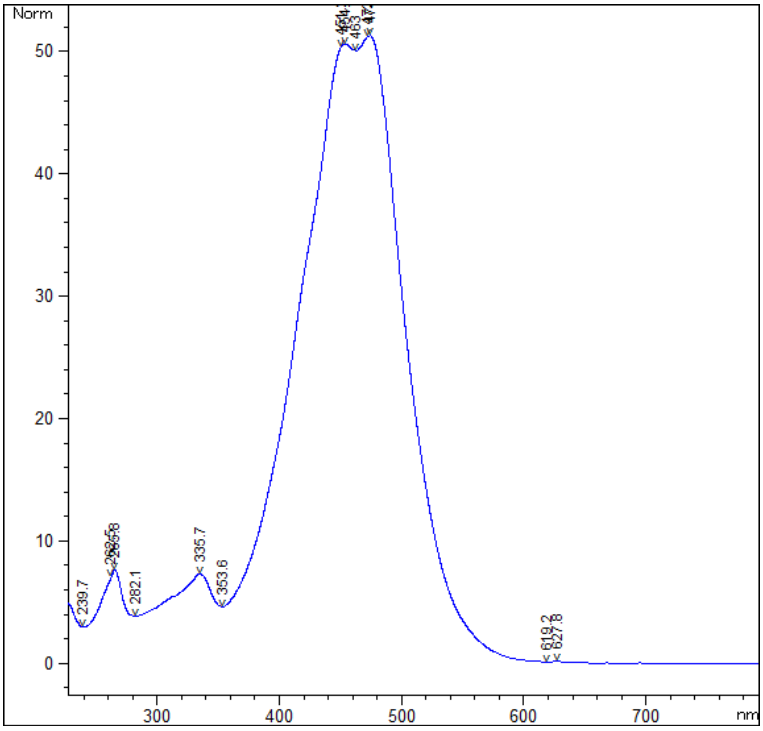

Norm
463
239.7
282.1
335.7
353.6
619.2
627.8
300
400
500
600
700
nm

Car7

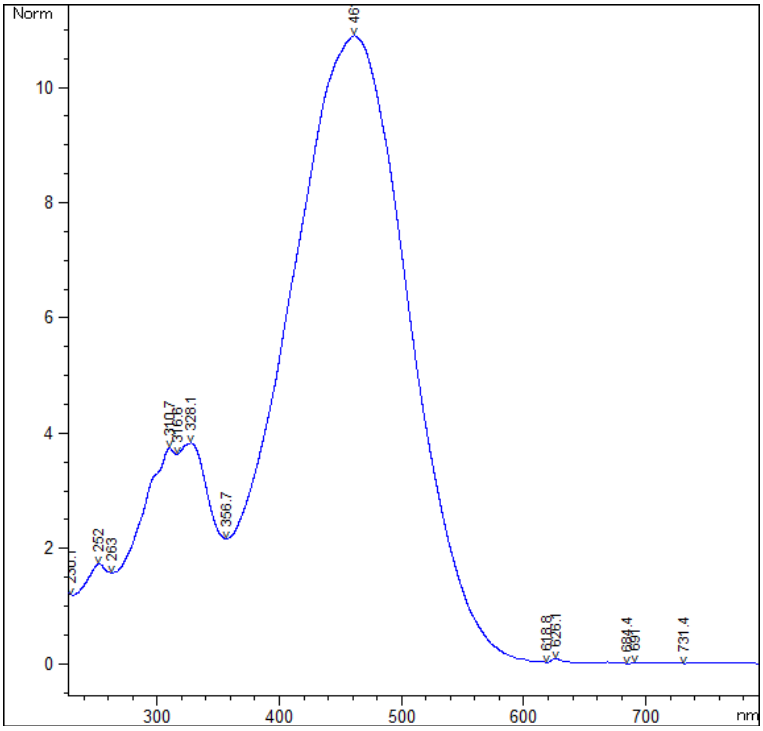
Norm
10
8
6
4
2
0
300
400
500
600
700
nm
46
310.7
316.6
328.1
356.7
252
263
618.8
626.1
691
731.4

Car8

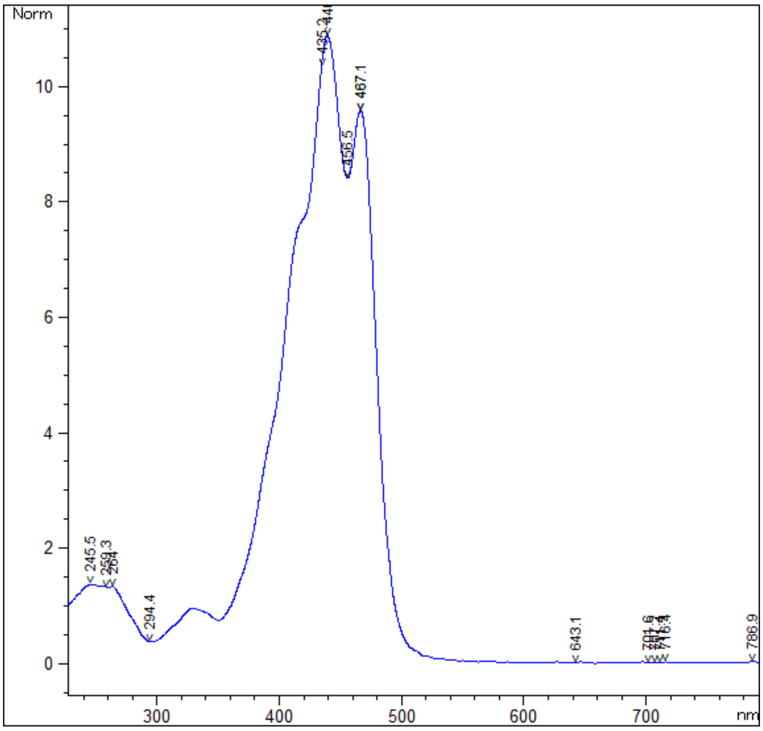
Norm
10
8
6
4
2
0
300
400
500
600
700
nm
435.3
467.1
456.5
245.5
259.3
264
294.4
643.1
701.6
786.9

Car9

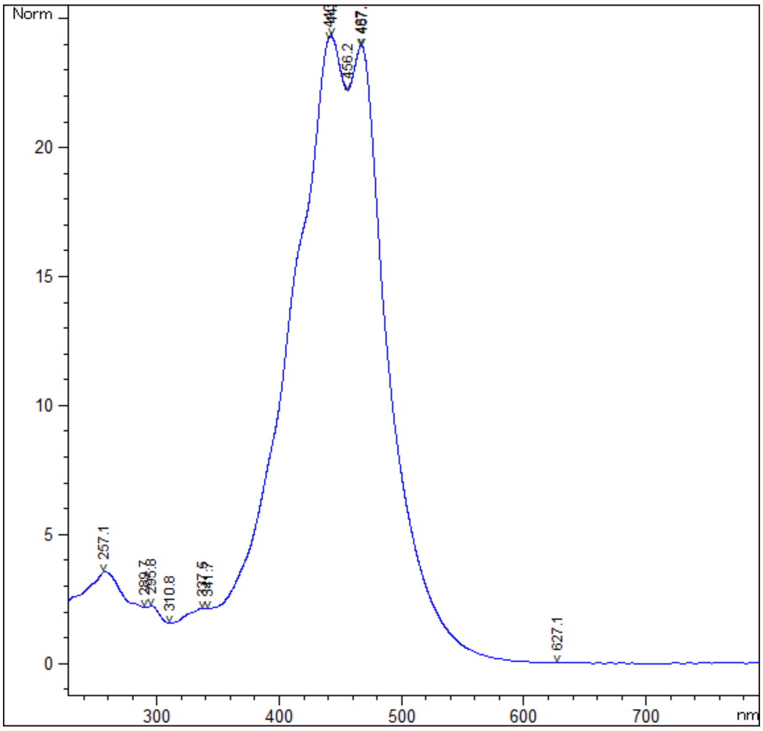

Norm
20
15
10
5
0
300
400
500
600
700
nm
257.1
310.8
456.2
627.1

Car10

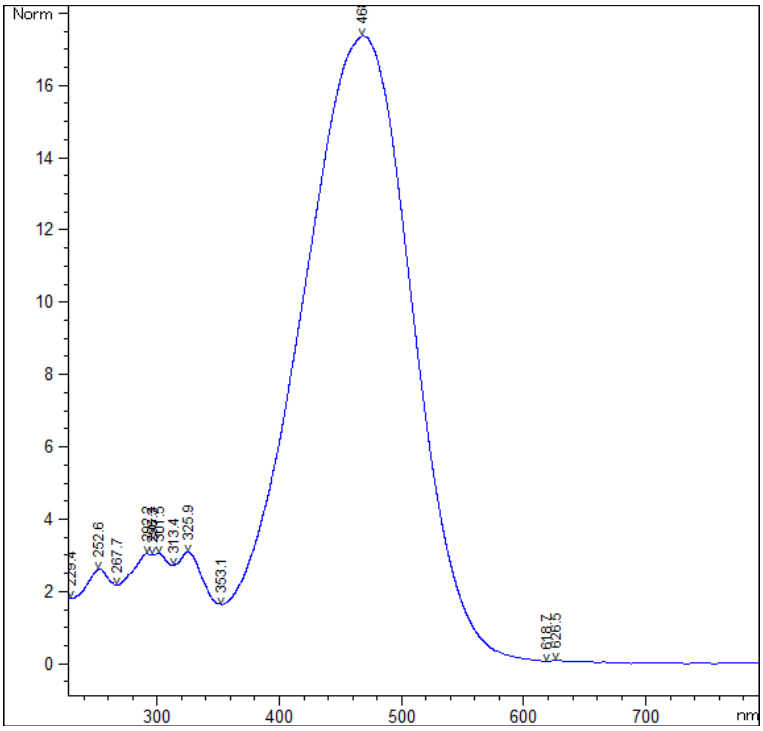

Norm
16
14
12
10
8
6
4
2
0
300
400
500
600
700
nm
252.6
267.7
313.4
325.9
353.1

Car11

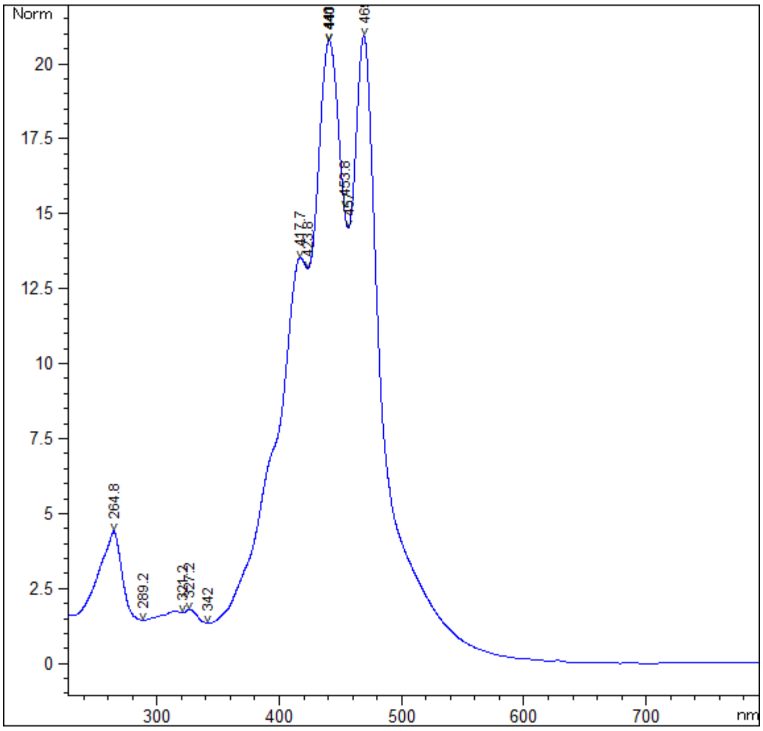

Norm
264.8
289.2
321.2
327.2
342
417.7
423.8
440
453.8
300
400
500
600
700
nm

Car12

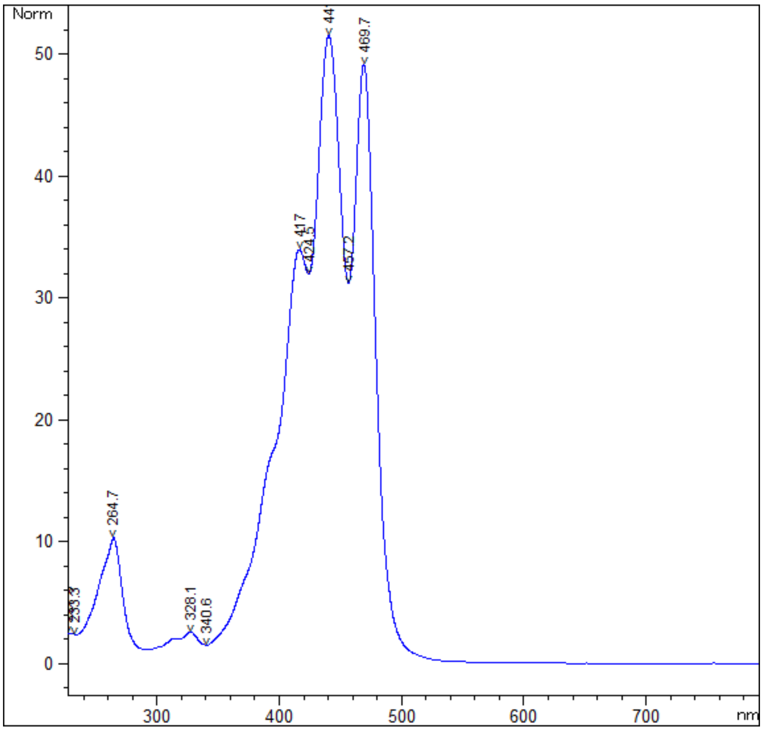

Norm
264.7
328.1
340.6
417
424.5
457.2
469.7
300
400
500
600
700
nm

Car13

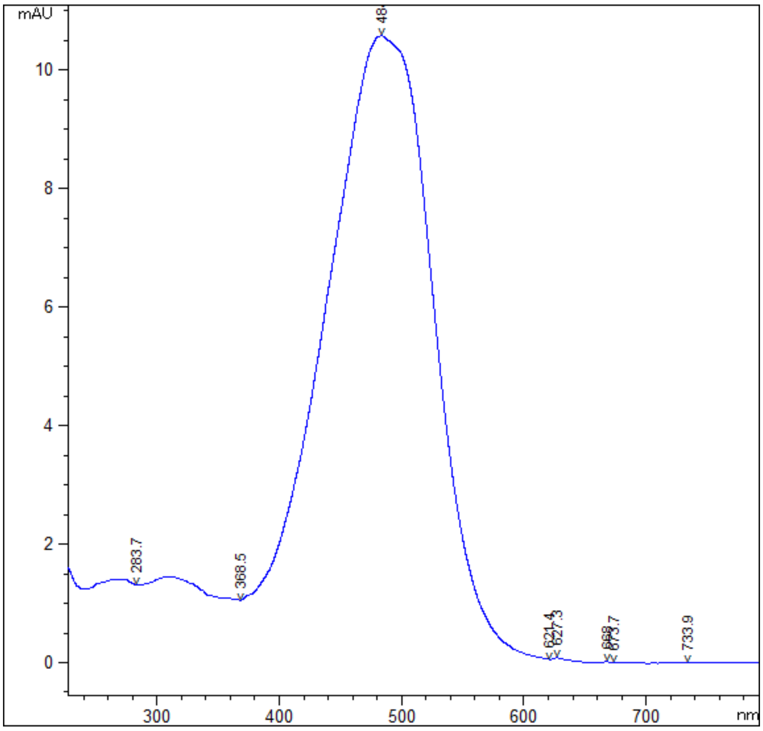
mAU
10
8
6
4
2
0
300
400
500
600
700
nm
283.7
368.5
733.9

Car14

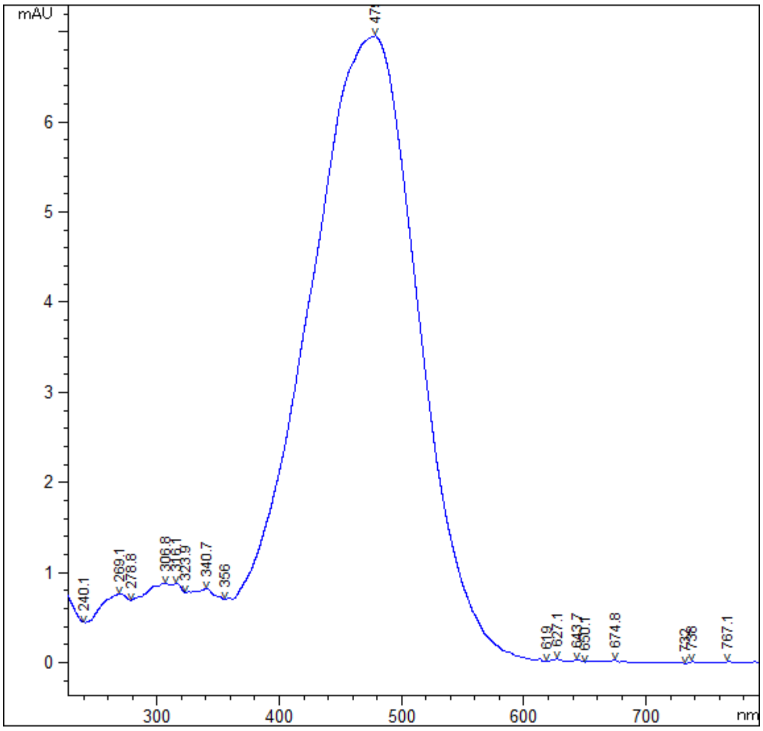
mAU
6
5
4
3
2
1
0
300
400
500
600
700
nm
240.1
269.1
278.8
306.8
340.7
356
619
627.1
674.8
767.1

Car15

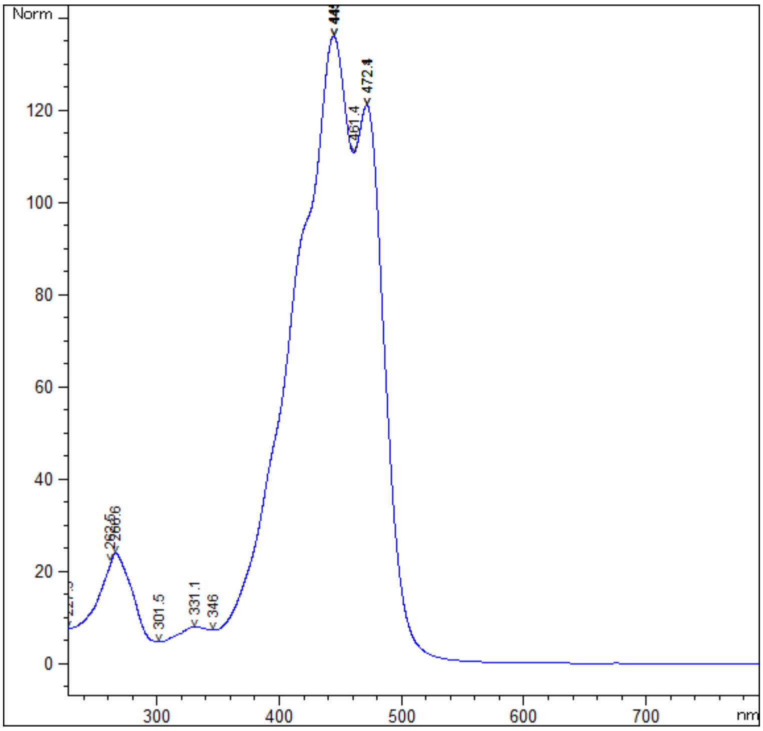
Norm
120
100
80
60
40
20
0
300
400
500
600
700
nm
472.4
461.4
301.5
331.1
346

Car16

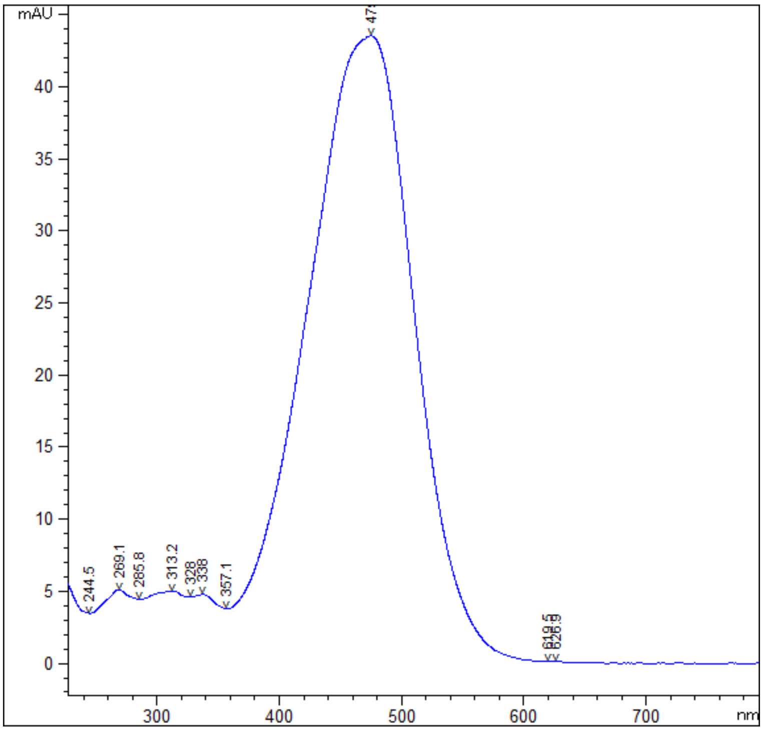
mAU
40
35
30
25
20
15
10
5
0
300
400
500
600
700
nm
244.5
269.1
285.8
313.2
328
338
357.1
619.5
626.9

Car17

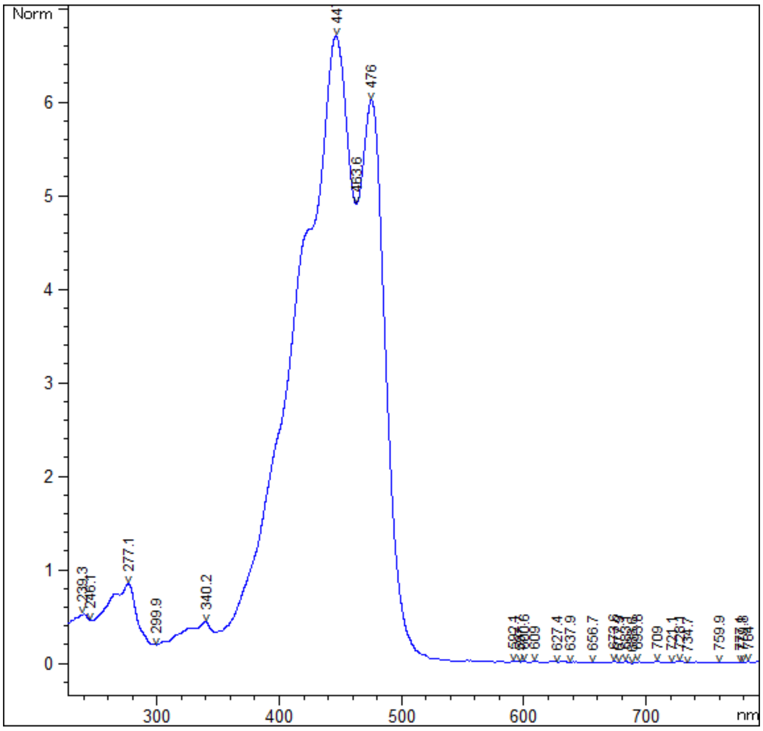
Norm
6
5
4
3
2
1
0
300
400
500
600
700
nm
239.3
246.1
277.1
299.9
340.2
463.6
476
609
627.4
637.9
656.7
709
759.9

Car18

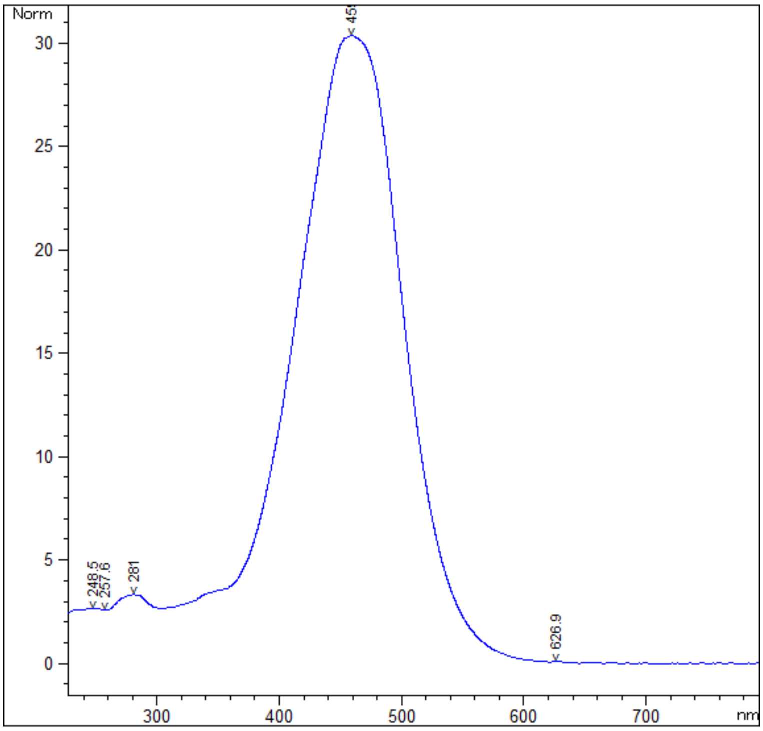
Norm
30
25
20
15
10
5
0
300
400
500
600
700
nm
248.5
257.6
281
626.9

Car19

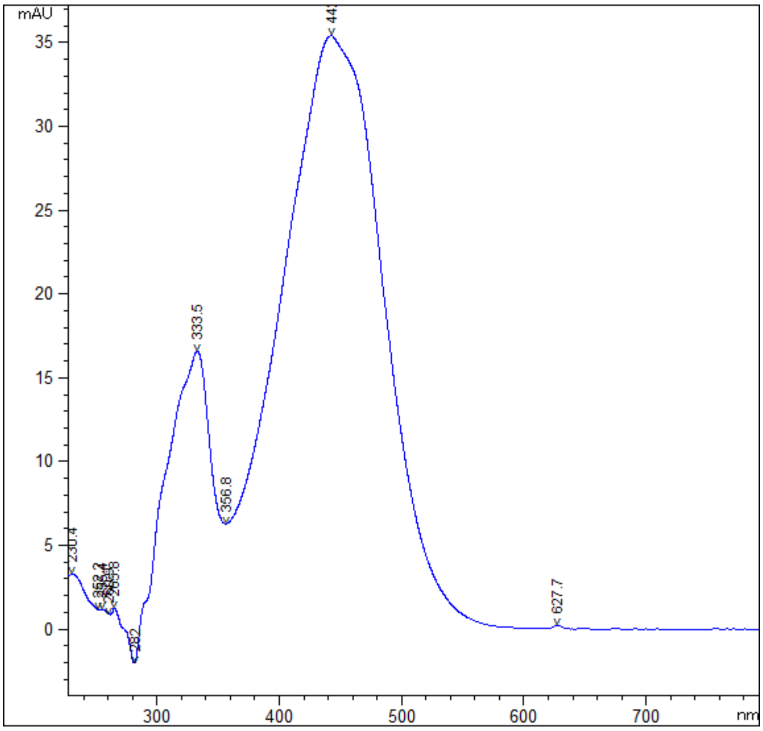

mAU
35
30
25
20
15
10
5
0
441
333.5
356.8
230.4
282
627.7
300
400
500
600
700
nm

Car20

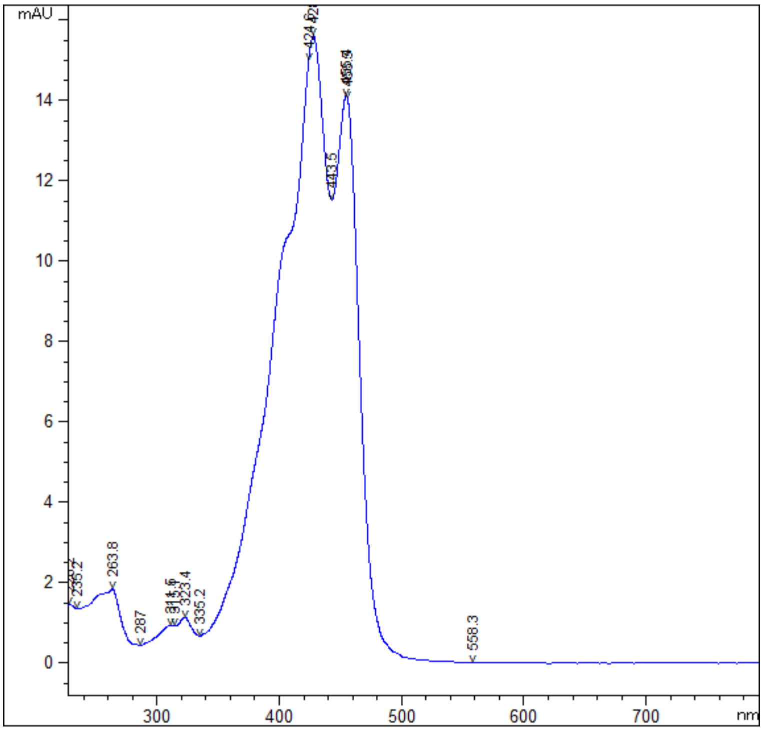

mAU
14
12
10
8
6
4
2
0
424.6
443.5
263.8
235.2
287
323.4
335.2
558.3
300
400
500
600
700
nm

Car21

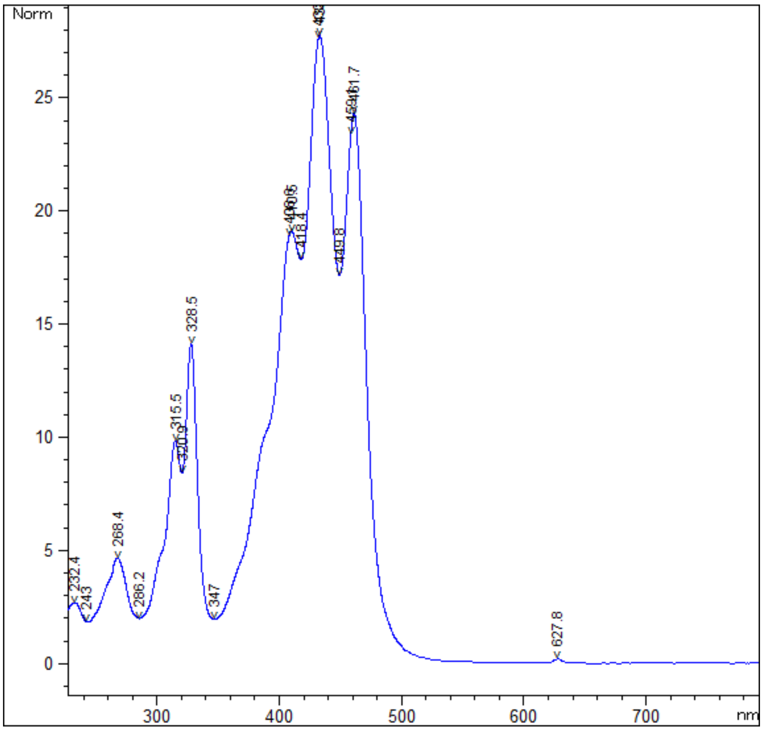

Norm
25
20
15
10
5
0
300
400
500
600
700
nm
461.7
418.4
328.5
315.5
268.4
232.4
243
286.2
347
627.8

Car22

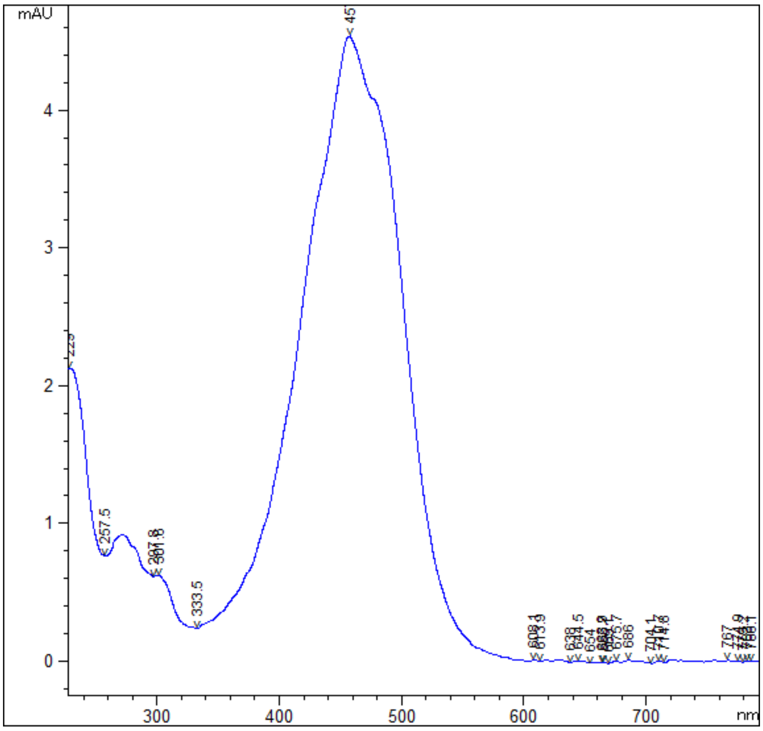

mAU
4
3
2
1
0
300
400
500
600
700
nm
257.5
333.5
686

Car23

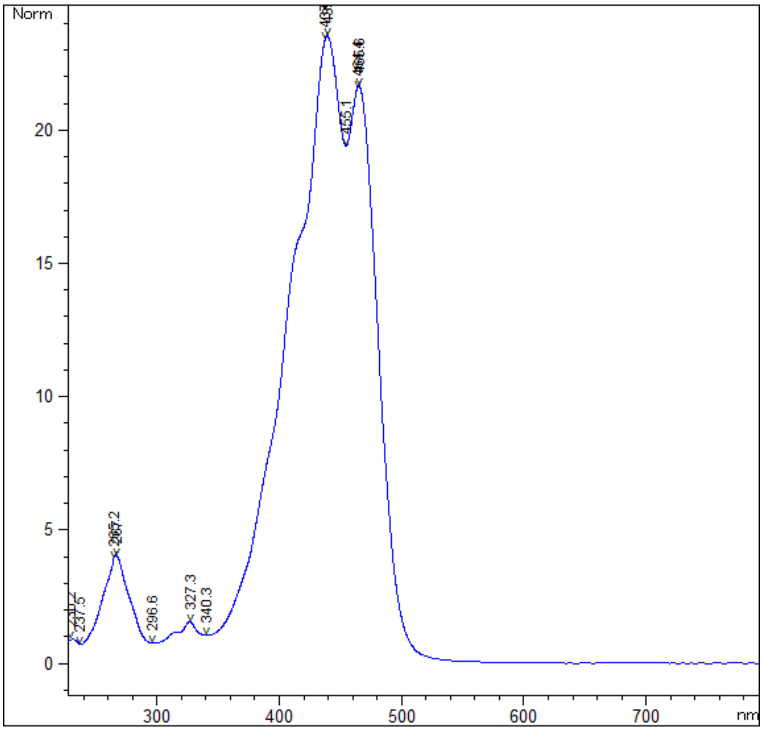
Norm
0
5
10
15
20
300
400
500
600
700
nm
267.2
296.6
327.3
340.3
237.5
455.1

Car24

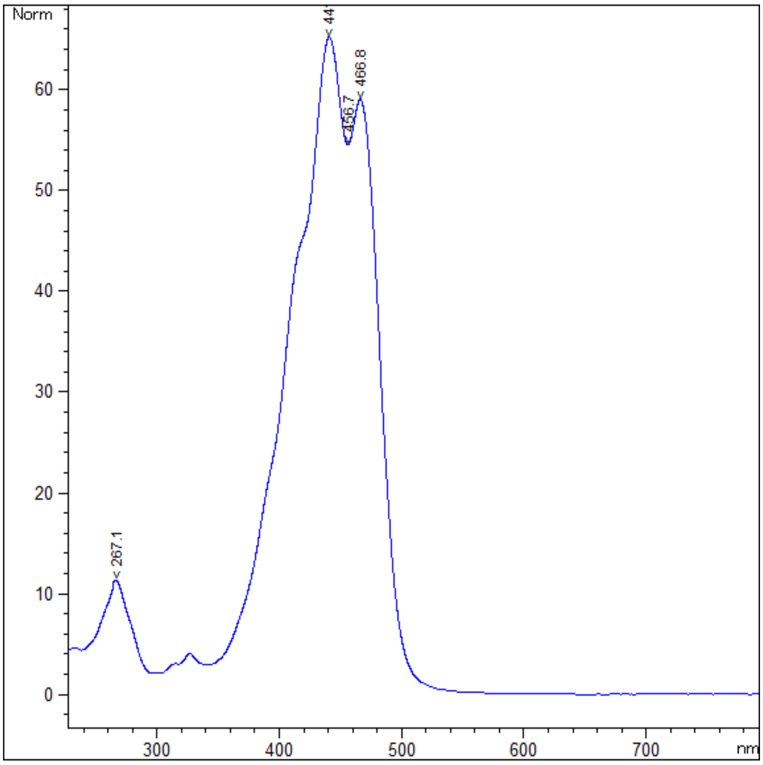
Norm
0
10
20
30
40
50
60
300
400
500
600
700
nm
267.1
456.7
466.8

Car25

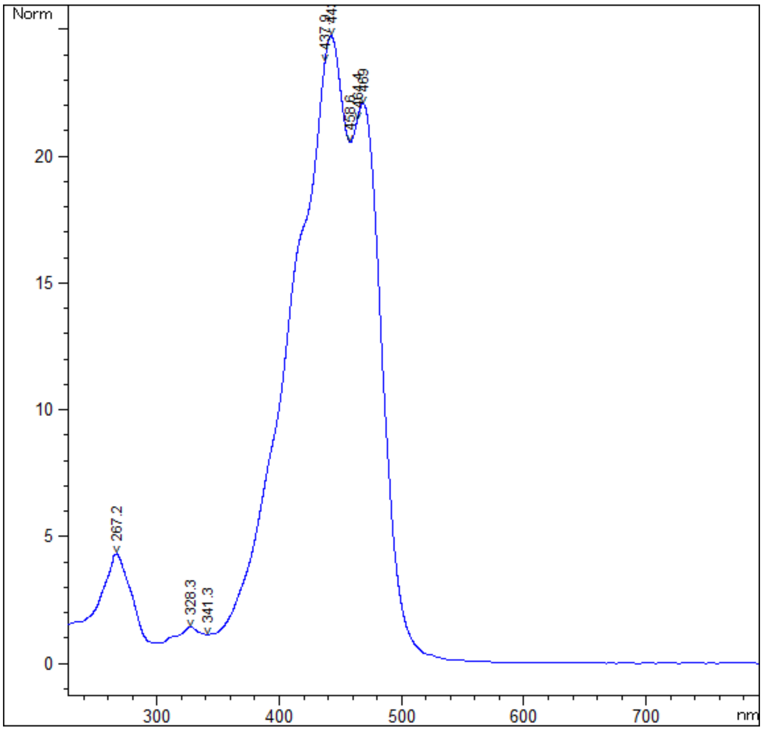
Norm
20
15
10
5
0
300
400
500
600
700
nm
267.2
328.3
341.3
437.9
458.6
469

Car 26

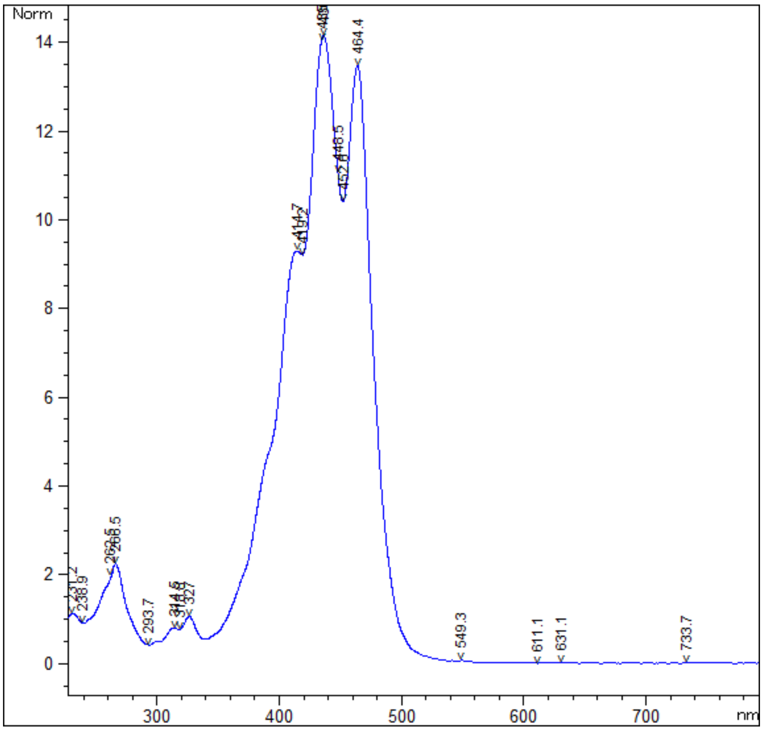
Norm
14
12
10
8
6
4
2
0
300
400
500
600
700
nm
238.9
266.5
293.7
327
414.7
446.5
464.4
549.3
611.1
631.1
733.7

Car27

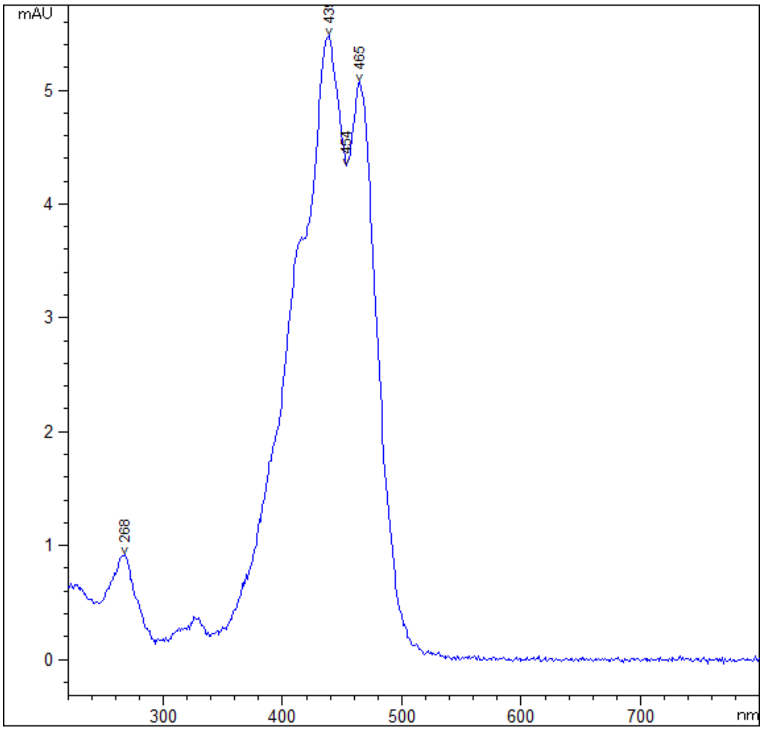
mAU
5
4
3
2
1
0
300
400
500
600
700
nm
268
465
454

Car28

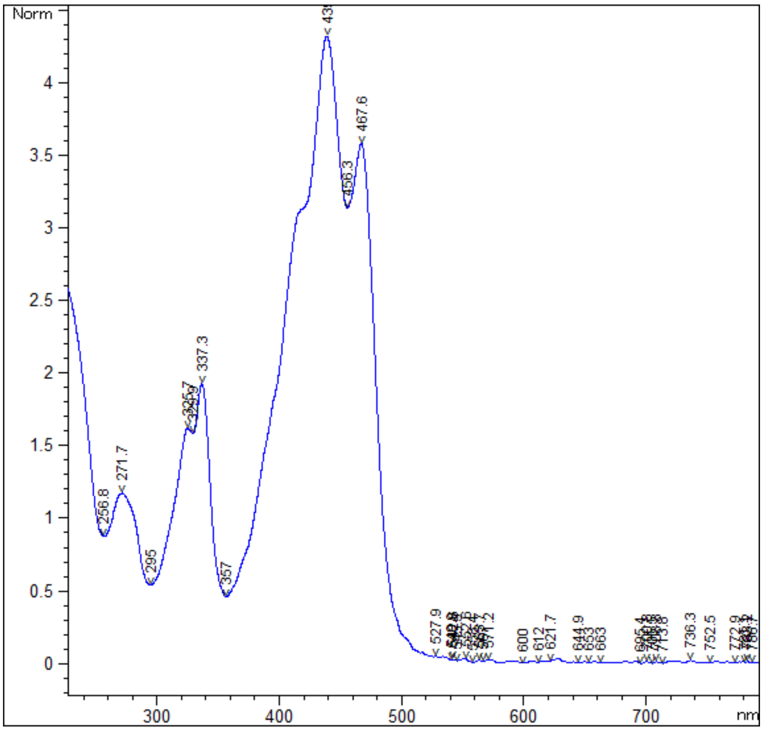
Norm
4
3.5
3
2.5
2
1.5
1
0.5
0
300
400
500
600
700
nm
467.6
456.3
337.3
271.7
256.8
295
357
527.9
571.2
600
612
621.7
644.9
653
663
736.3
752.5

Car29

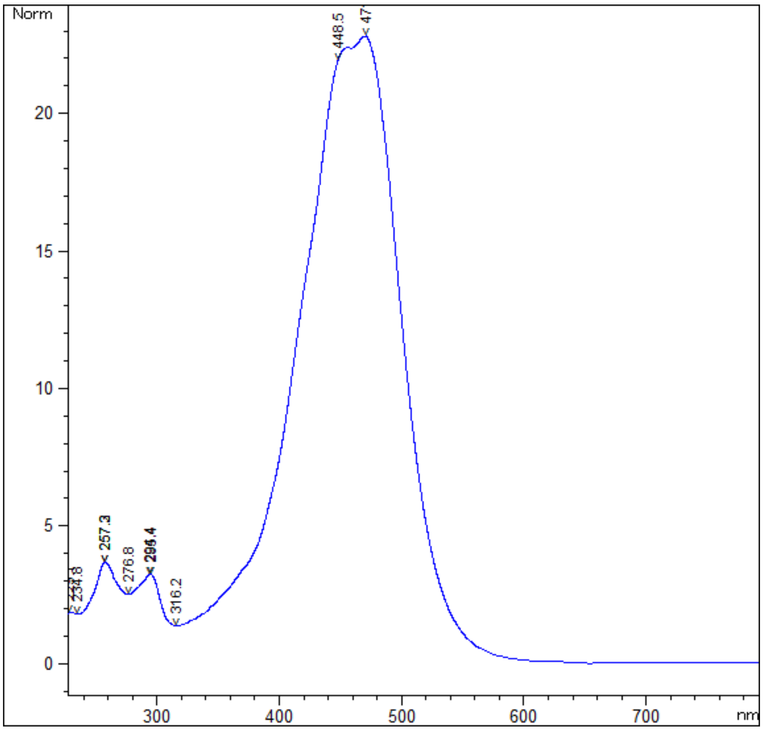
Norm
20
15
10
5
0
300
400
500
600
700
nm
448.5
257.3
276.8
316.2

Car30

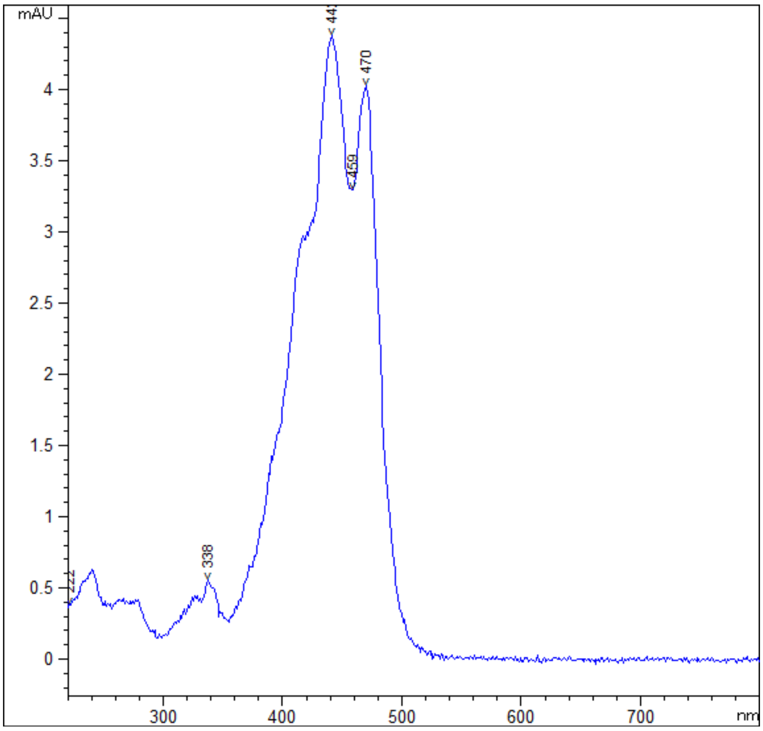
mAU
4
3.5
3
2.5
2
1.5
1
0.5
0
300
400
500
600
700
nm
470
459
338

Car31

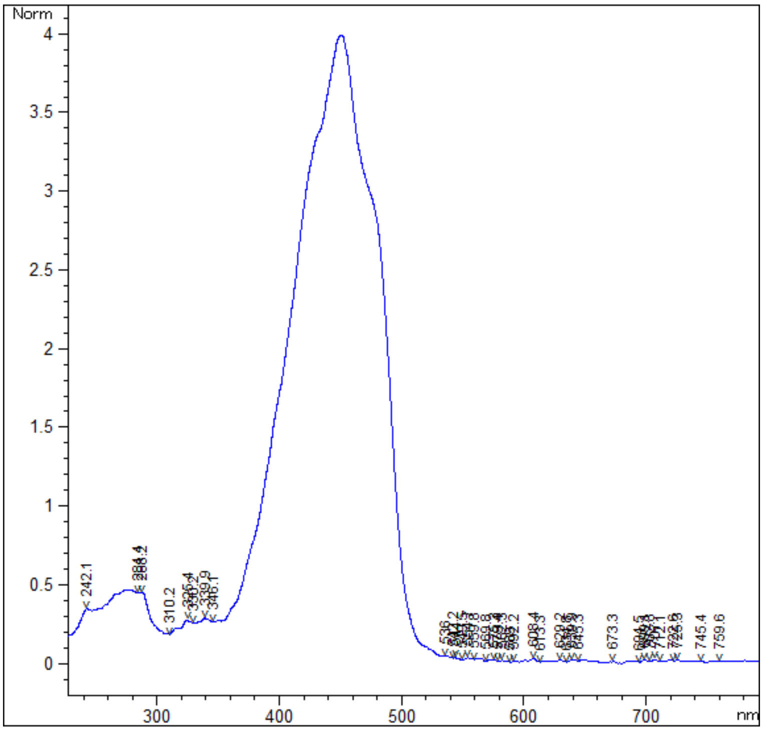

Norm
4
3.5
3
2.5
2
1.5
1
0.5
0
242.1
310.2
346.1
673.3
745.4
759.6
300
400
500
600
700
nm

Car32

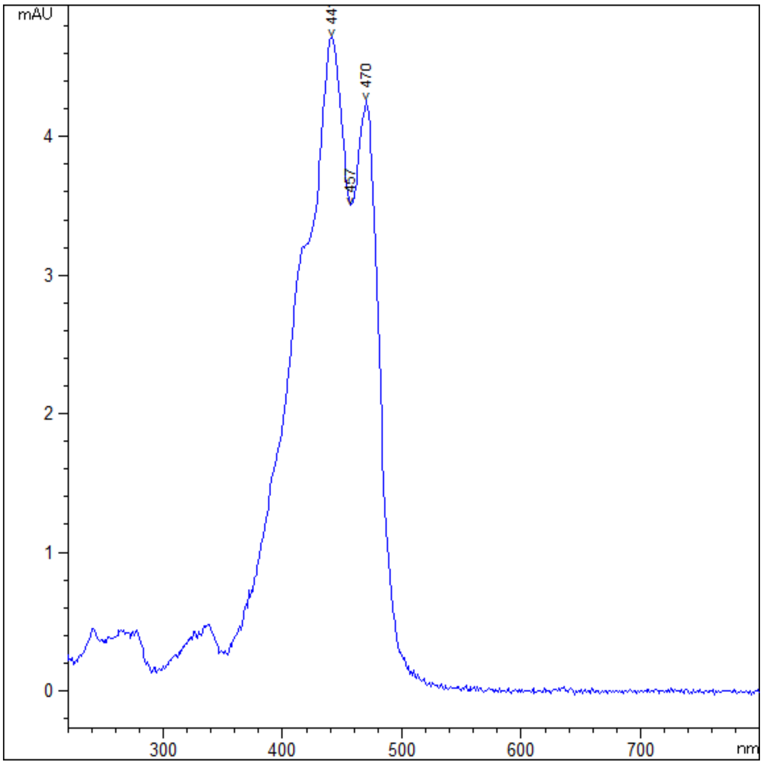

mAU
4
3
2
1
0
470
457
300
400
500
600
700
nm

Car33

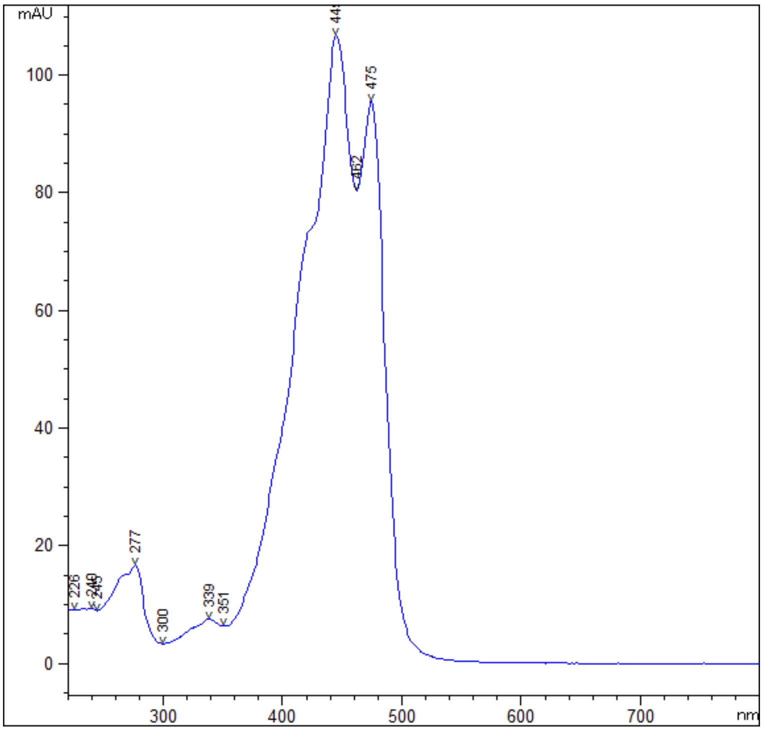
mAU
100
80
60
40
20
0
300
400
500
600
700
nm
449
475
462
277
226
240
245
300
339
351

Car34

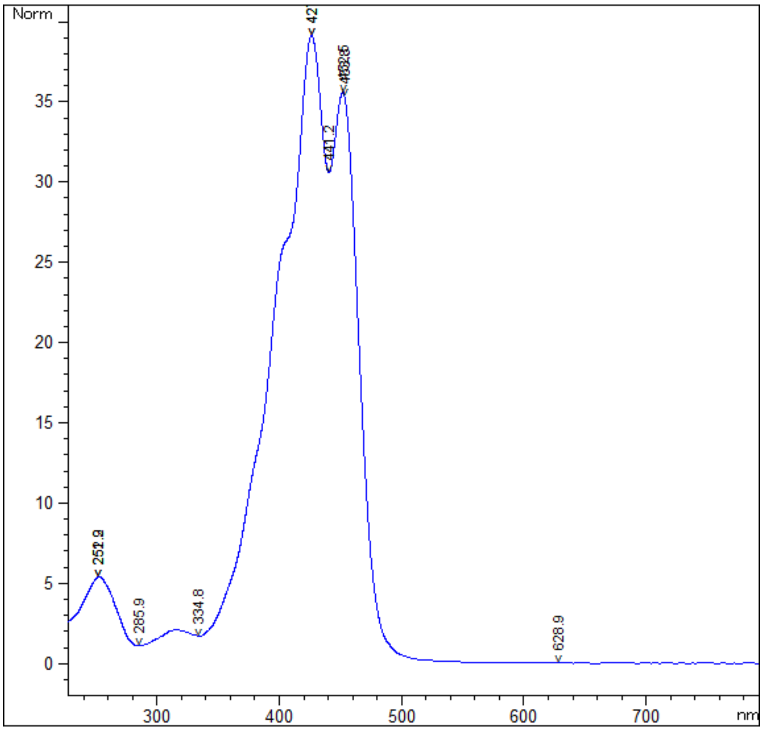
Norm
35
30
25
20
15
10
5
0
300
400
500
600
700
nm
427
441.2
252.9
285.9
334.8
628.9

Car35

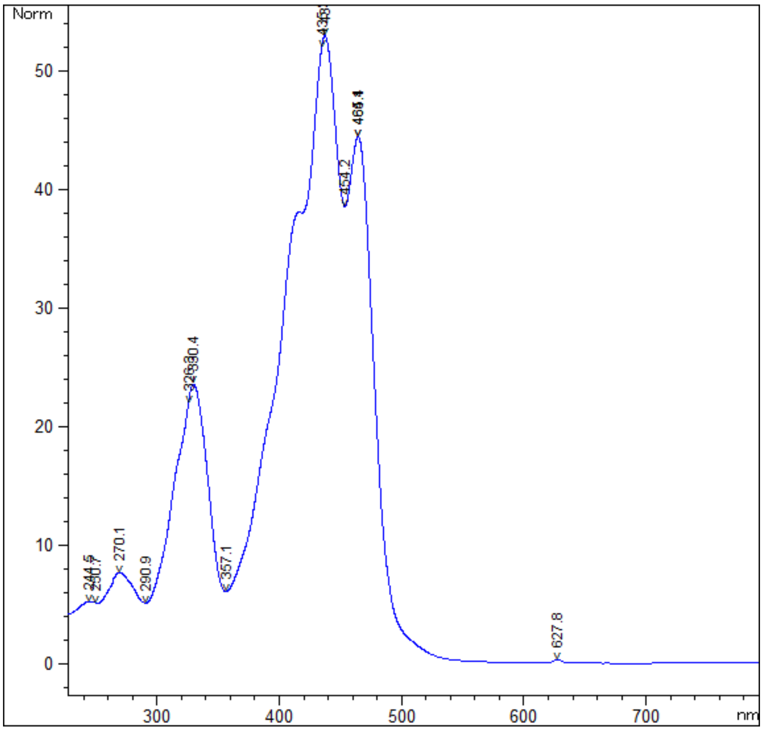
Norm
50
40
30
20
10
0
300
400
500
600
700
nm
270.1
290.9
357.1
454.2
627.8

Car36

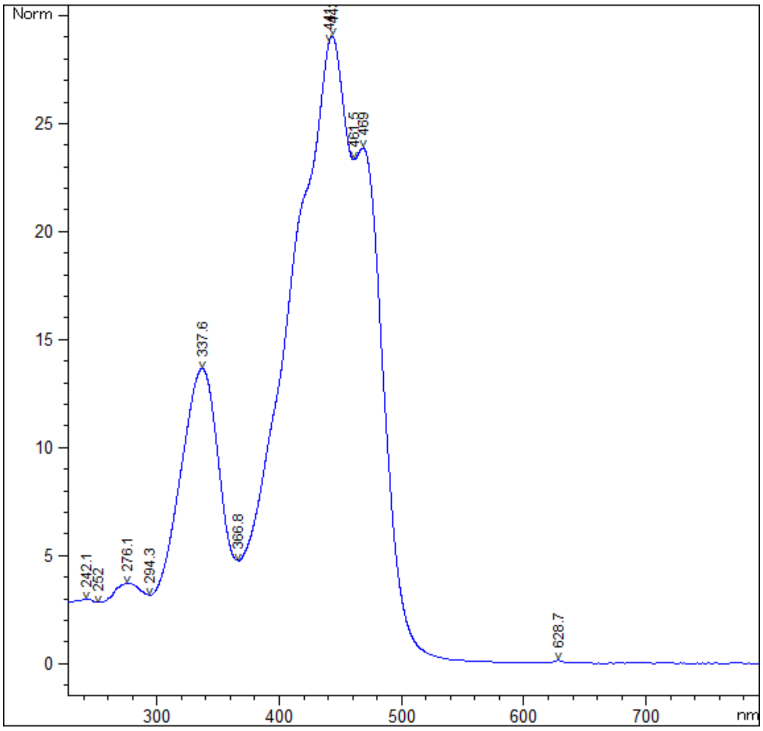
Norm
25
20
15
10
5
0
300
400
500
600
700
nm
242.1
252
276.1
294.3
337.6
366.8
461.5
469
628.7

Car37

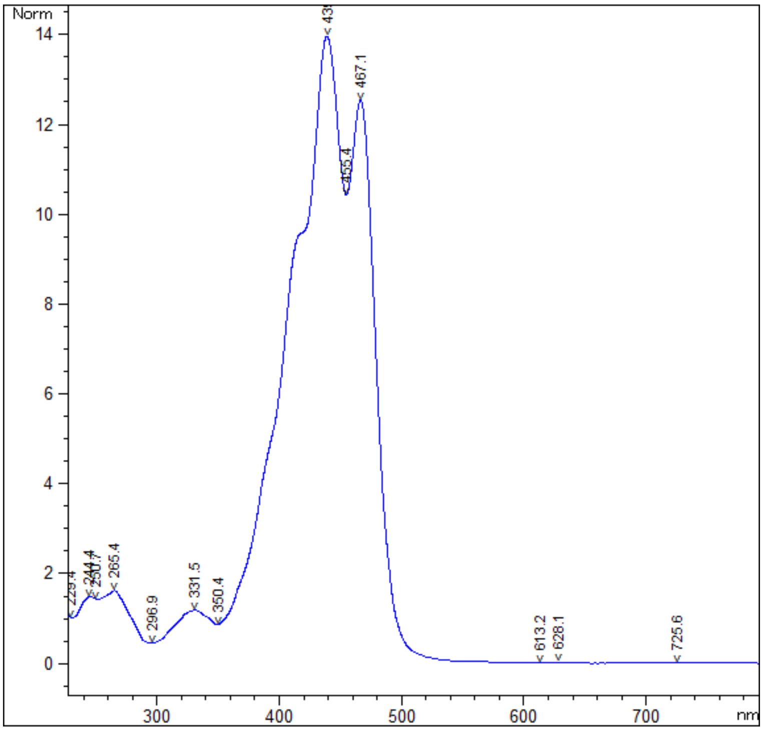
Norm
14
12
10
8
6
4
2
0
300
400
500
600
700
nm
467.1
455.4
265.4
296.9
331.5
350.4
613.2
628.1
725.6

Car38

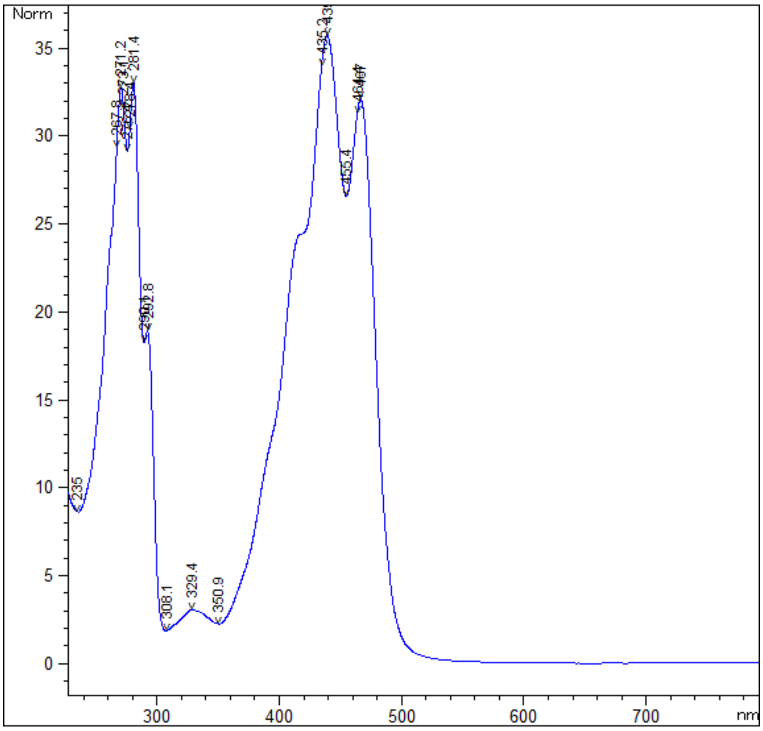
Norm
35
30
25
20
15
10
5
0
300
400
500
600
700
nm
281.4
435.3
455.4
235
308.1
329.4
350.9

Car39

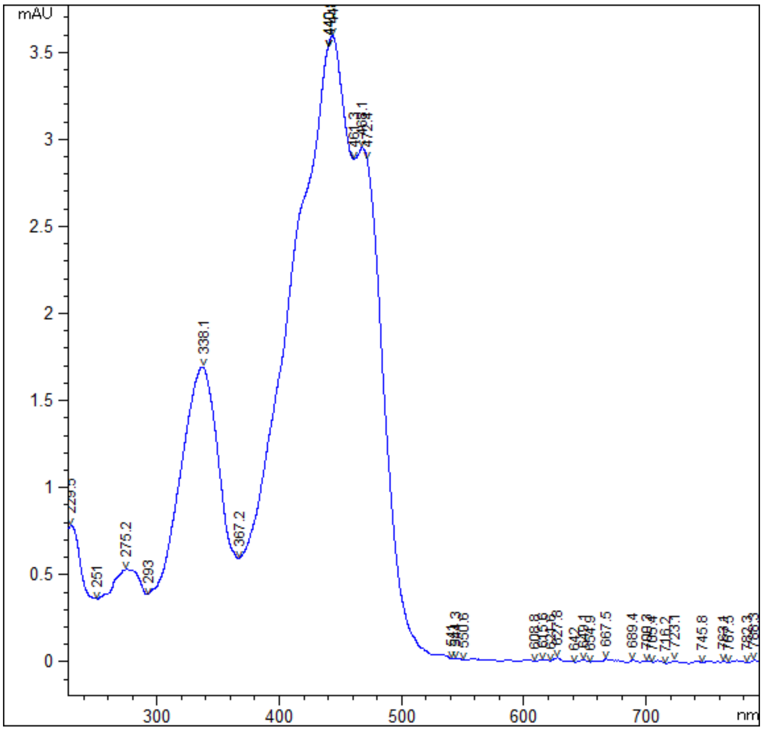
mAU
3.5
3
2.5
2
1.5
1
0.5
0
300
400
500
600
700
nm
338.1
275.2
251
293
367.2
667.5
689.4
745.8

Car40

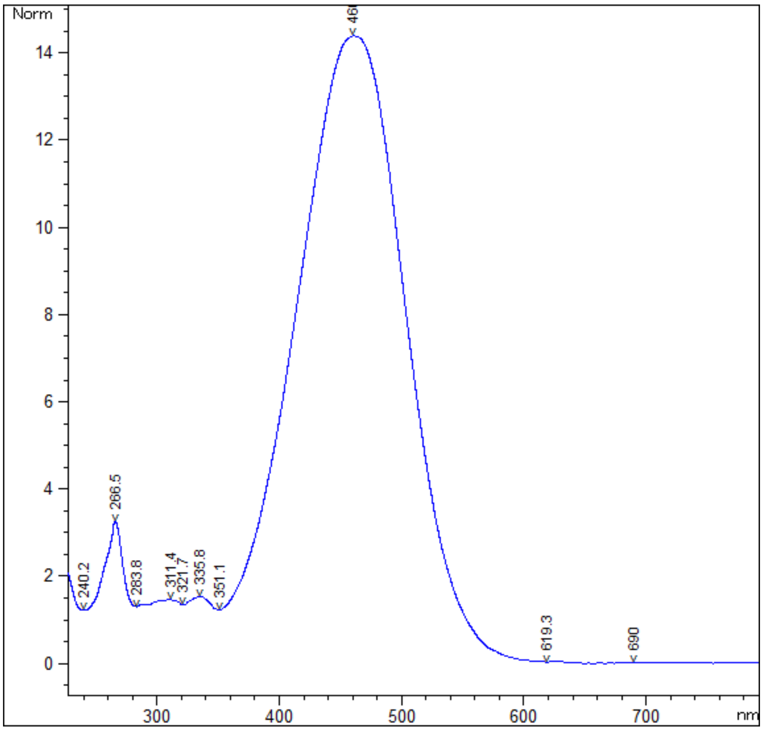
Norm
14
12
10
8
6
4
2
0
300
400
500
600
700
nm
266.5
240.2
283.8
311.4
321.7
335.8
351.1
619.3
690

Car41

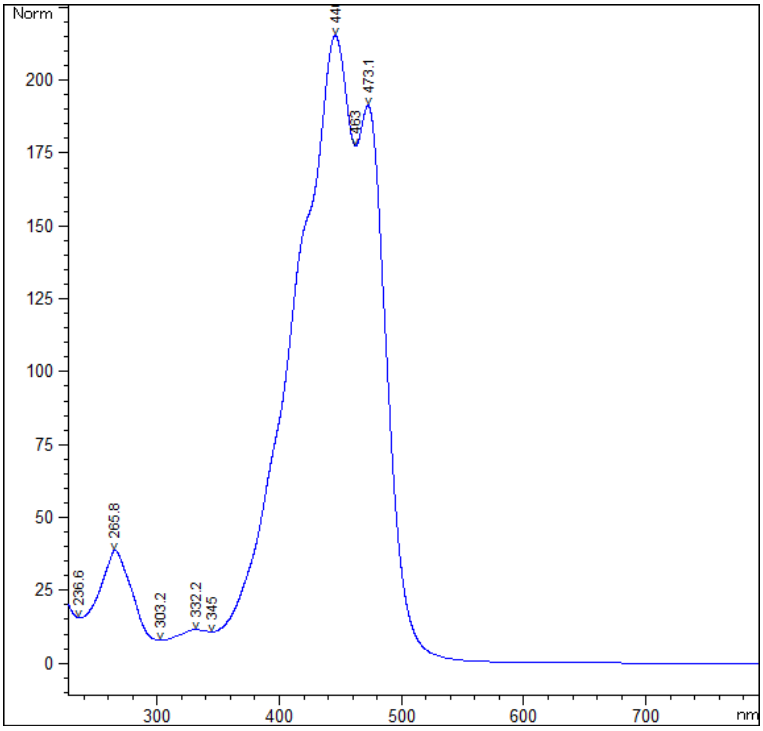
Norm
200
175
150
125
100
75
50
25
0
300
400
500
600
700
nm
265.8
236.6
303.2
332.2
345
463
473.1

Car42

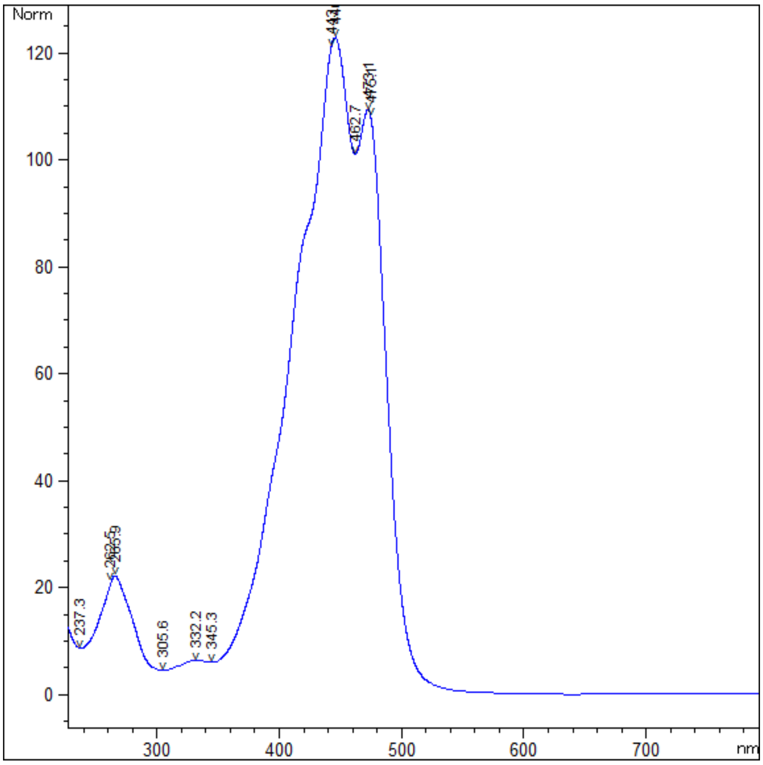
Norm
120
100
80
60
40
20
0
300
400
500
600
700
nm
237.3
305.6
332.2
345.3
462.7

Car43

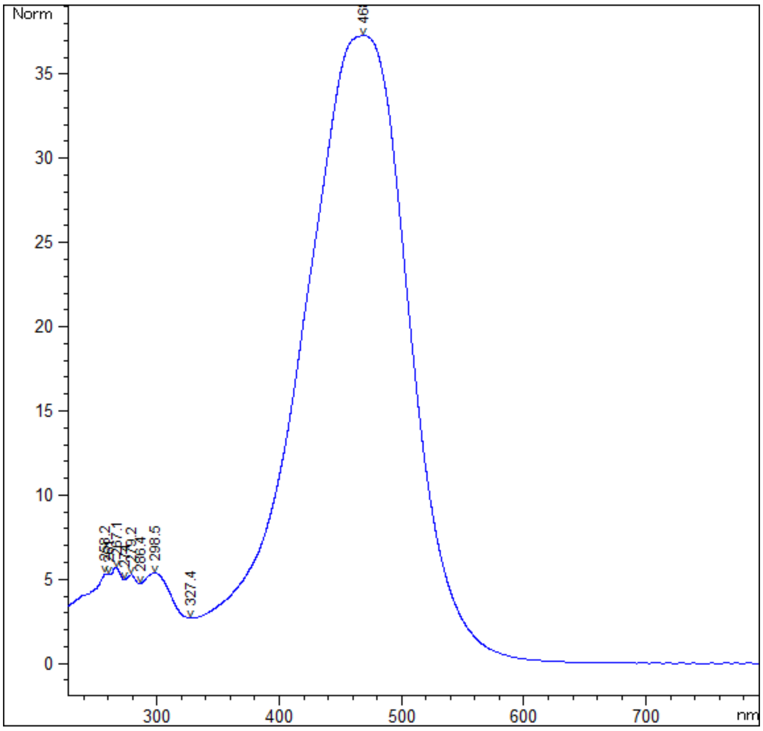
Norm
35
30
25
20
15
10
5
0
300
400
500
600
700
nm
298.5
327.4

Car44

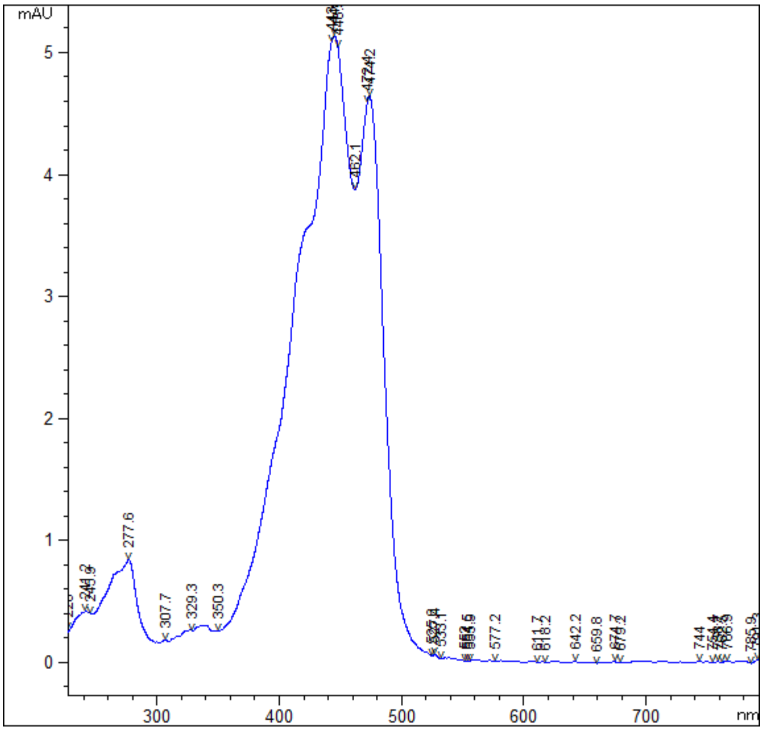
mAU
5
4
3
2
1
0
300
400
500
600
700
nm
277.6
307.7
329.3
350.3
462.1
577.2
611.7
618.2
642.2
659.8
744

Car45

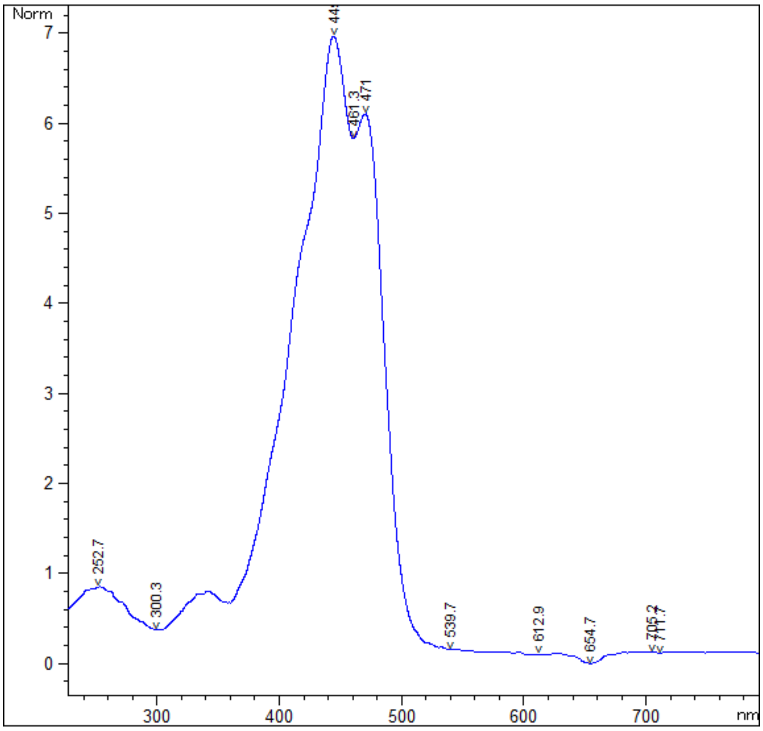
Norm
7
6
5
4
3
2
1
0
300
400
500
600
700
nm
252.7
300.3
461.3
471
539.7
612.9
654.7

Car46

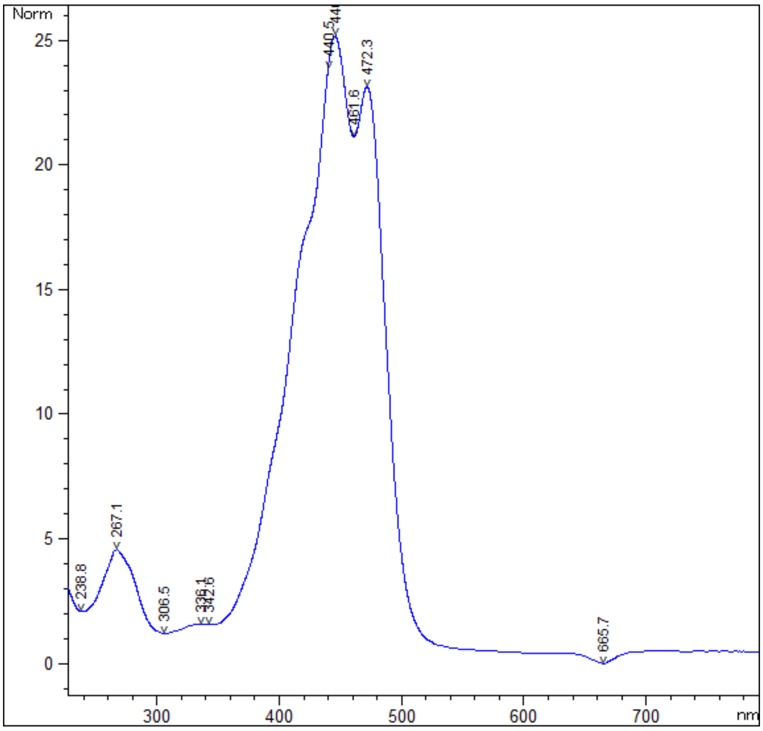
Norm
25
20
15
10
5
0
300
400
500
600
700
nm
238.8
267.1
306.5
440.5
461.6
472.3
665.7

Car47

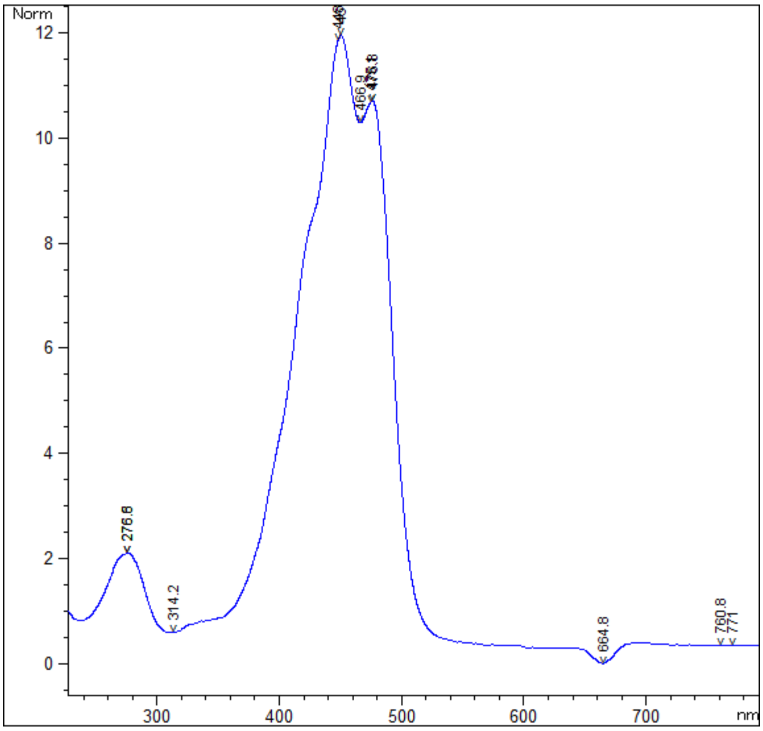
Norm
12
10
8
6
4
2
0
300
400
500
600
700
nm
276.8
314.2
466.9
664.8
760.8
771

Car48

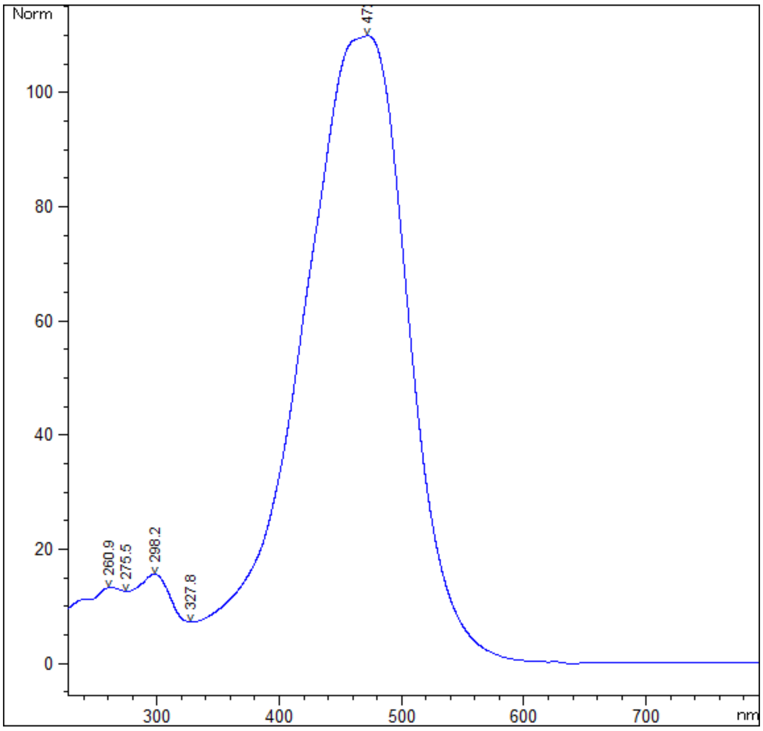
Norm
100
80
60
40
20
0
300
400
500
600
700
nm
260.9
275.5
298.2
327.8

Car49

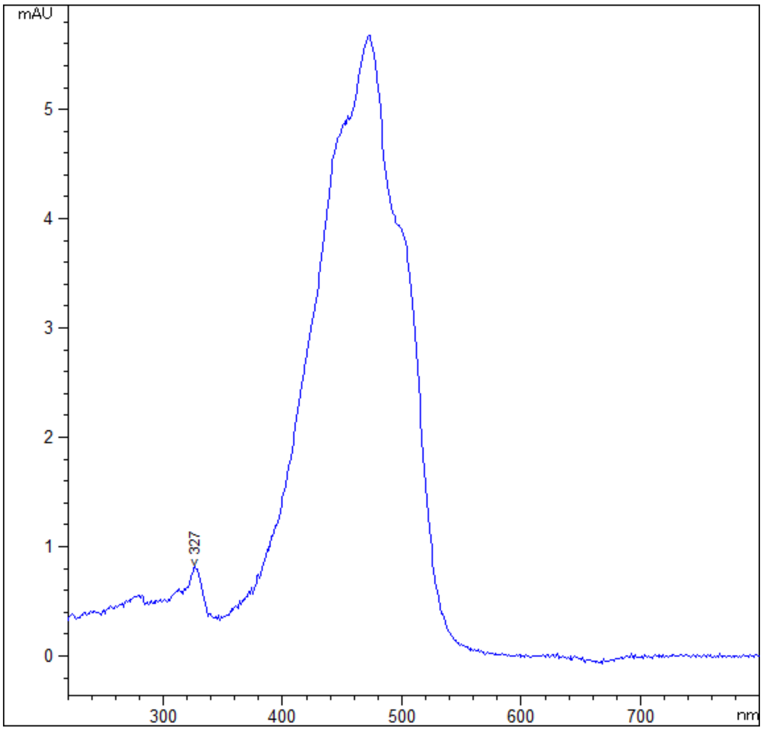
mAU
5
4
3
2
1
0
< 327
300
400
500
600
700
nm

Car50

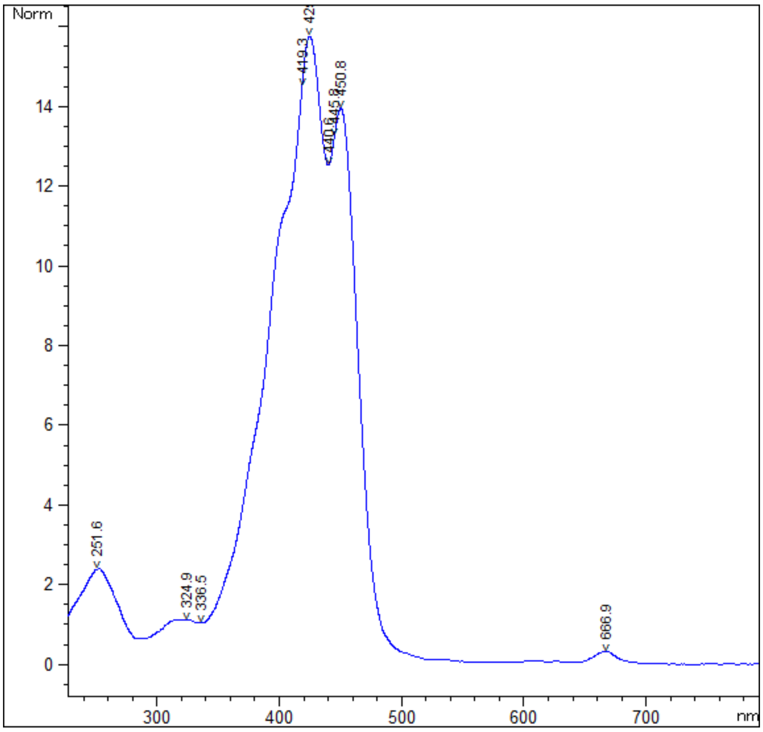
Norm
14
12
10
8
6
4
2
0
< 251.6
< 324.9
< 336.5
< 419.3
< 450.8
< 666.9
300
400
500
600
700
nm

Car51

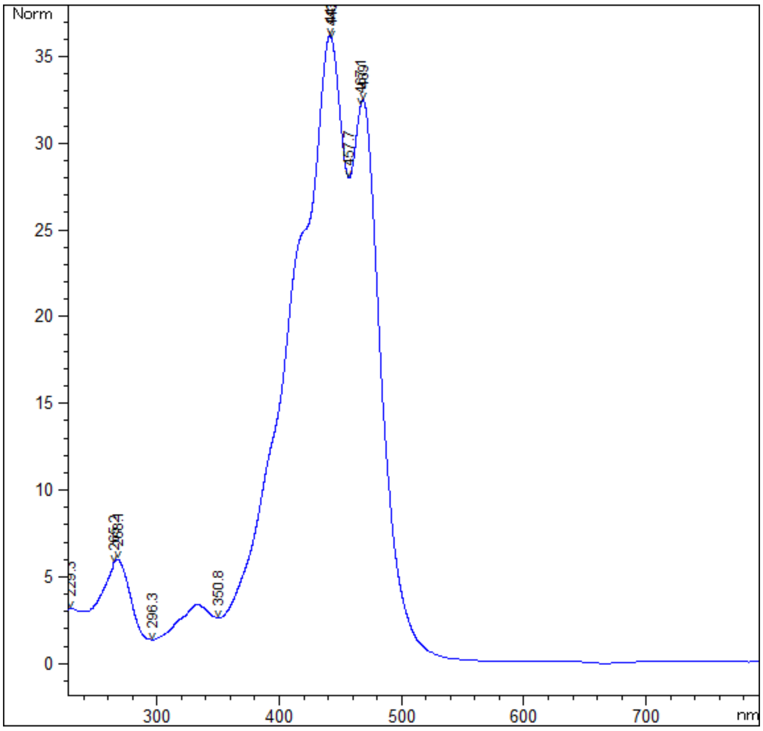
Norm
35
30
25
20
15
10
5
0
300
400
500
600
700
nm
296.3
350.8
457.7

Car52

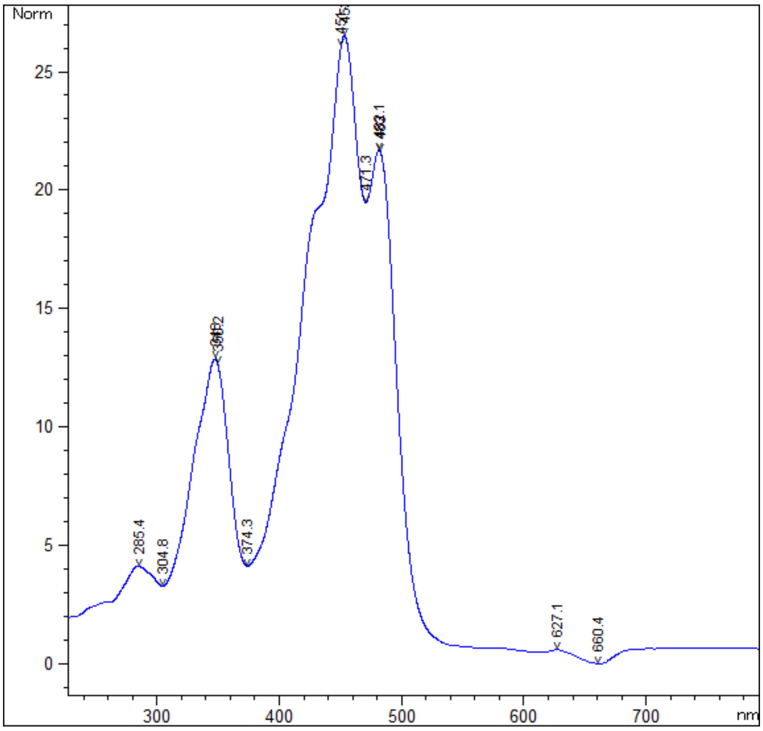
Norm
25
20
15
10
5
0
300
400
500
600
700
nm
285.4
304.8
374.3
471.3
627.1
660.4

Car53

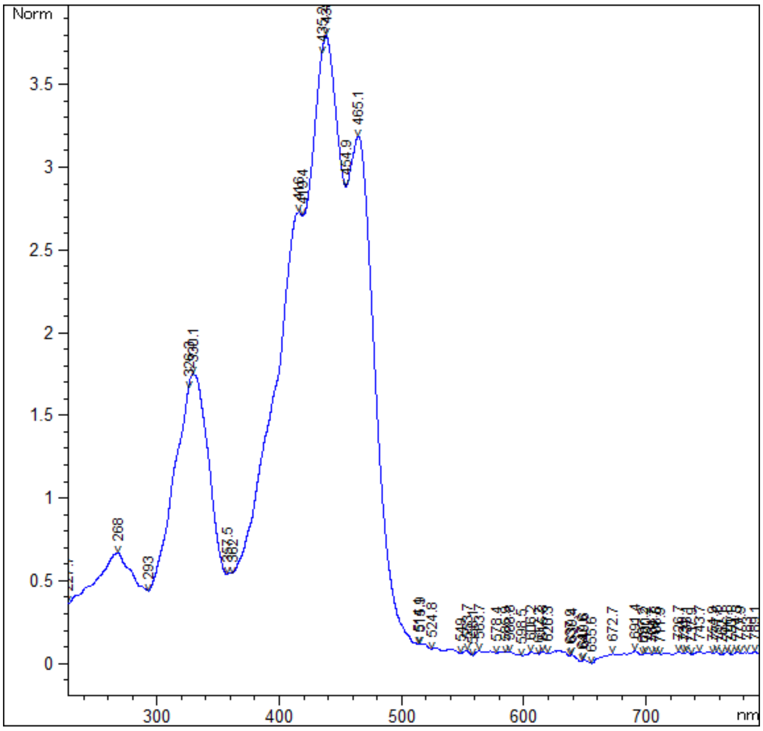
Norm
3.5
3
2.5
2
1.5
1
0.5
0
300
400
500
600
700
nm
268
293
330.1
357.5
362
454.9
465.1
524.8
578.4
672.7
691.4

Car54

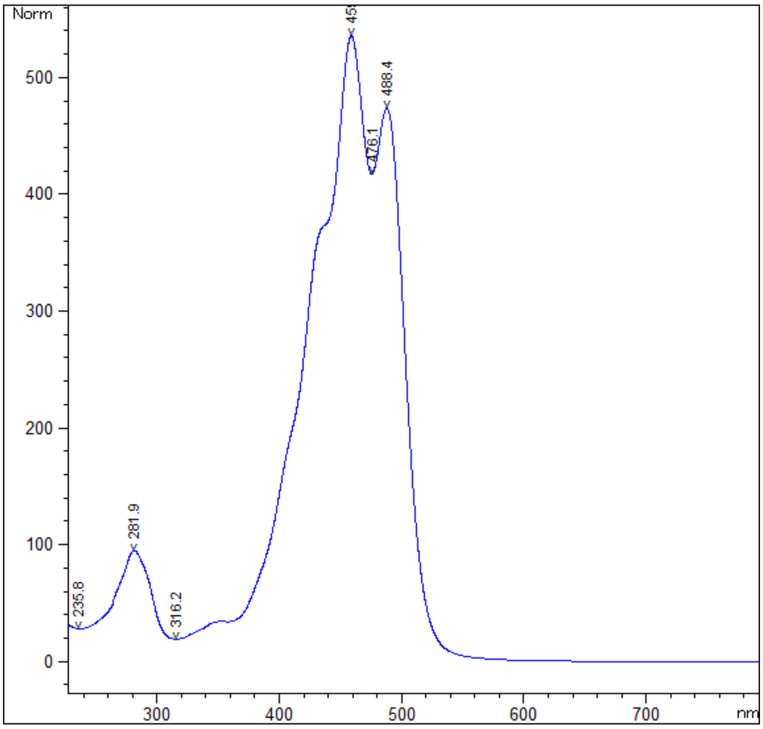
Norm
500
400
300
200
100
0
300
400
500
600
700
nm
235.8
281.9
316.2
476.1
488.4

Car55

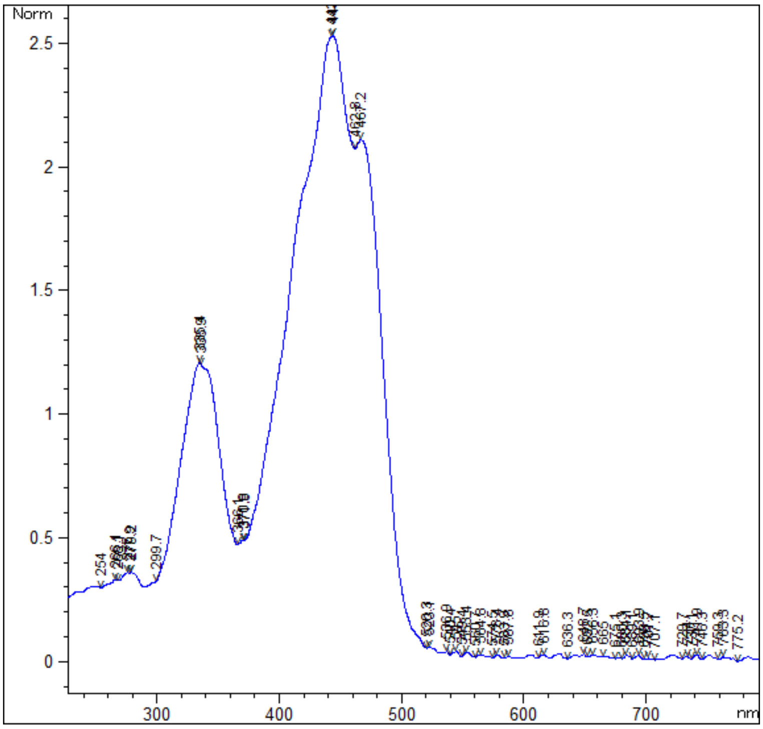
Norm
2.5
2
1.5
1
0.5
0
300
400
500
600
700
nm
254
299.7
467.2
636.3
775.2

Car56

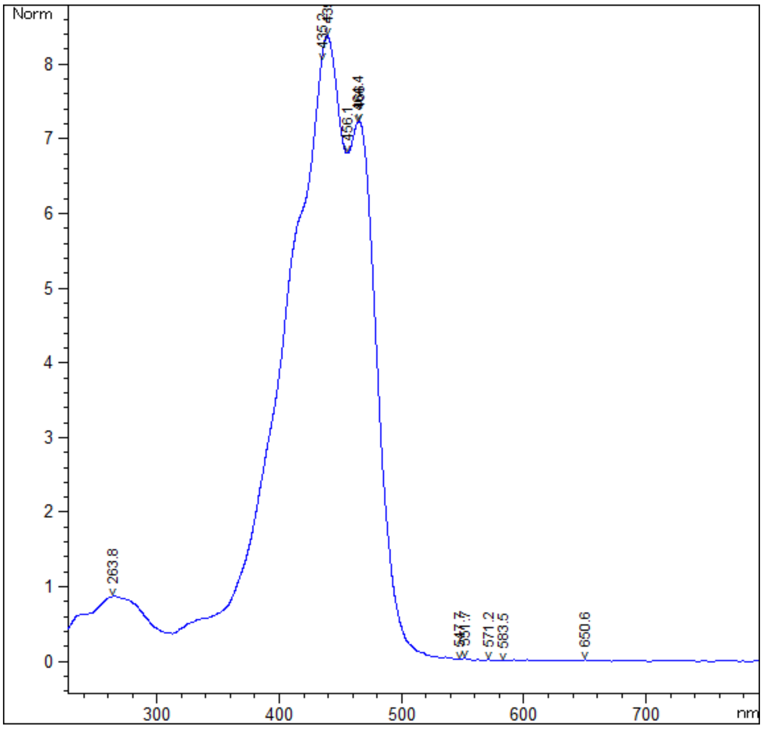
Norm
8
7
6
5
4
3
2
1
0
300
400
500
600
700
nm
263.8
435.2
456.1
547.7
571.2
583.5
650.6

Car57

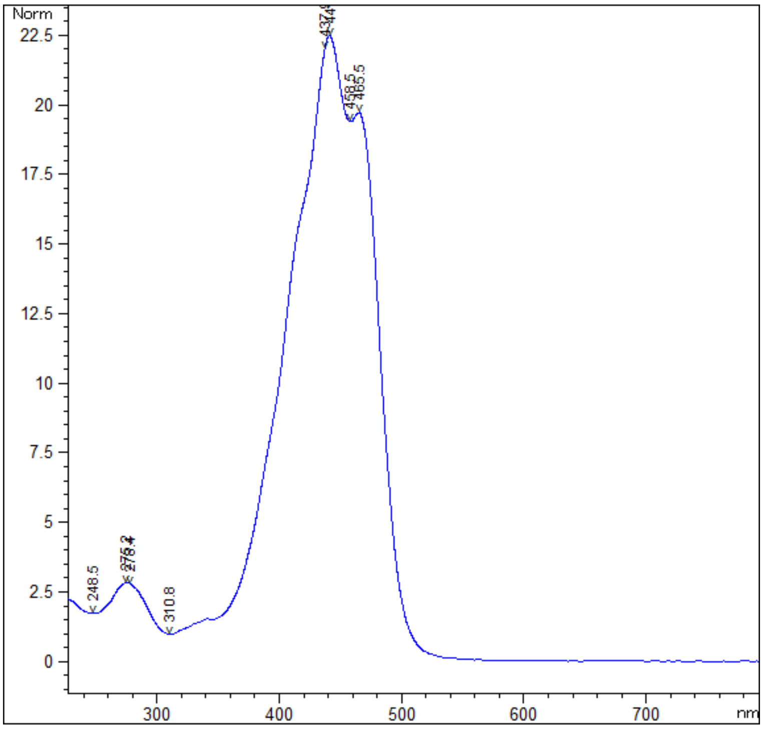
Norm
22.5
20
17.5
15
12.5
10
7.5
5
2.5
0
248.5
310.8
458.5
465.5
300
400
500
600
700
nm
