# Supplementary material for: Community analysis of pigment patterns from 37 microalgae strains reveals new carotenoids and porphyrins characteristic of distinct strains and taxonomic groups
Source: PLoS One. 2017 Feb 23;12(2):e0171872. doi: 10.1371/journal.pone.0171872 (PMC5322898; doi:10.1371/journal.pone.0171872)
Supplement: S1 Table — (PDF) [file pone.0171872.s004.pdf]

Supporting Information

S2 Table. Pigments and derivatives currently used as chemotaxonomic markers of phytoplankton strains, species and classes.

Algal lineage or pigment type	Ancillary pigments markers described (minor or variable occurrence)	Reference
Bolidophyceae	Chl c_3-like pigment	(1)
Chatonnella cf. verrucosa	Moraxanthin	(2)
CHLORO-1	Astaxanthin; Monadoxanthin (unusual rare occurrence)	(3)
CHRYSO-1	Antheraxanthin	(4)
CRYPTO-1	Crocoxanthin; Monadoxanthin; α-carotene	(5)
Dinoflagellates Chloroplaste type-1	P-457; Peridinin; Peridinol; Dinoxanthin	(6)
Dinoflagellates Chloroplaste type-2	Keto-19'-hex-fuco-like ; Gyroxanthin diester; Keto-19'-but-fuco-like; 4-keto-19'-but-fuco; β,Ɛ-carotene	(6)
Dinoflagellates Chloroplaste type-3	Gyroxanthin diester; β,Ψ-carotene	(6)
Dinoflagellates Chloroplaste type-4	γ-carotene; Canthaxanthin (significant but not always present)	(6)
Dinoflagellates Chloroplaste type-5	Alloxanthin ; β,Ɛ-carotene	(6)
Dinoflagellates Chloroplaste type 6	*trans*-neoxanthin; Unidentified Carotenoid Lc	(6)
EUGLENO-1	Eutreptiellanone	(7)
EUSTIG-1	Antheraxanthin; Canthaxanthin (significant but not always present); Vauchieraxanthin and esters	(8)
Haptophyte pigment type-5	4-k-fucoxanthin	(9)
Haptophyte pigment type-7	Chl c_2-MGDG [14/14] (significant but not always present)	(9)
MESOSTIG-1	Lycopene; *trans*-neoxanthin	(10)
Mesostigmatophyceae Streptophyta	Siphonaxanthin and esters	(11)
PELAGO-1	Ɛ,Ɛ-carotene	(12)
Pelagophytes	Gyroxanthin diester	(12)
PRASINO-3B	Dihydrolutein; Micromonal; Micromonol; Unidentified Car-*M. pusilla*; Uriolide	(13)
Silicoflagellates	Chl *a* like	(14)
TREBOUXIO-1	Astaxanthin	(4)
XANTHO-1	Heteroxanthin	(15)

References

1. Guillou L, Chrétiennot-Dinet M-J, Medlin LK, Claustre H, Goër SL, Vaulot D. Bolidomonas: A New Genus with Two Species Belonging to a New Algal Class, the Bolidophyceae (heterokonta). Journal of Phycology. 1999 avril;35(2):368–81.

2. Mangoni O, Imperatore C, Tomas CR, Costantino V, Saggiomo V, Mangoni A. The New Carotenoid Pigment Moraxanthin Is Associated with Toxic Microalgae. Marine Drugs. 2011 Feb 10;9(2):242–55.

3. Lewin RA, Krienitz L, Goericke R, Takeda H, Hepperle D. Picocystis salinarum gen. et sp. nov. (Chlorophyta) – a new picoplanktonic green alga. Phycologia. 2000 Nov 1;39(6):560–5.

4. Jeffrey SW, Wright SW, Zapata M. Microalgal class and their signature pigments. In: Phytoplankton pigments: Characterization, chemotaxonomy and applications in oceanography. Cambridge University Press; 2011. p. 3–77. (Environmental chemistry).

5. Rial P, Garrido JL, Jaén D, Rodríguez F. Pigment composition in three Dinophysis species (Dinophyceae) and the associated cultures of Mesodinium rubrum and Teleaulax amphioxeia. J Plankton Res. 2013 Mar 1;35(2):433–7.

6. Zapata M, Fraga S, Rodrguez F, Garrido JL. Pigment-based chloroplast types in dinoflagellates. Mar Ecol Prog Ser. 2012 Sep 28;465:33–52.

7. Bjørnland T. Chlorophylls and carotenoids of the marine alga Eutreptiella gymnastica. Phytochemistry. 1982;21(7):1715–9.

8. Brown JS. Functional Organization of Chlorophyll a and Carotenoids in the Alga, Nannochloropsis salina. Plant Physiol. 1987 Feb 1;83(2):434–7.

9. Zapata M, Jeffrey S, Wright S, Rodríguez F, Garrido J, Clementson L. Photosynthetic pigments in 37 species (65 strains) of Haptophyta: implications for oceanography and chemotaxonomy. Marine Ecology Progress Series. 2004;270:83–102.

10. Yoshii Y, Takaichi S, Maoka T, Inouye I. Photosynthetic Pigment Composition in the Primitive Green Alga Mesostigma Viride (prasinophyceae): Phylogenetic and Evolutionary Implications1. Journal of Phycology. 2003 juin;39(3):570–6.

11. Yoshii Y, Takaichi S, Maoka T, Suda S, Sekiguchi H, Nakayama T, et al. Variation of Siphonaxanthin Series Among the Genus Nephroselmis (prasinophyceae, Chlorophyta), Including a Novel Primary Methoxy Carotenoid1. Journal of Phycology. 2005 août;41(4):827–34.

12. Bjørnland T, Haxo FT, Liaaen-Jensen S. Carotenoids of the Florida red tide dinoflagellate Karenia brevis. Biochemical Systematics and Ecology. 2003 Oct;31(10):1147–62.

13. Brown MR, Jeffrey SW. Biochemical composition of microalgae from the green algal classes Chlorophyceae and Prasinophyceae. 1. Amino acids, sugars and pigments. Journal of Experimental Marine Biology and Ecology. 1992 Oct 12;161(1):91–113.

14. Edvardsen B, Eikrem W, Shalchian-Tabrizi K, Riisberg I, Johnsen G, Naustvoll L, et al. Verrucophora farcimen gen. et sp. nov. (Dictyochophyceae, Heterokonta)—a bloom-forming ichthyotoxic flagellate from the Skagerrak, Norway1. Journal of Phycology. 2007 Oct 1;43(5):1054–70.

15. Andersen RA, Potter D, Bidigare RR, Latasa M, Rowan K, O'Kelly CJ. Characterization and Phylogenetic position of the enigmatic golden alga Phaeothamnion confervicola: Ultrastructure, pigment composition and partial SSu rDNA sequence. Journal of Phycology. 1998 avril;34(2):286–98.
